# Unraveling Small Molecule-Mediated Sirtuin 3 Activation at a Distinct Binding Site for Cardioprotective Therapies

**DOI:** 10.1021/acscentsci.5c00023

**Published:** 2025-04-14

**Authors:** Dan Zhang, Jifa Zhang, Chengyong Wu, Yao Xiao, Liwei Ji, Jiarui Hu, Jianjun Ding, Tao Li, Yiwen Zhang, Liang Ouyang

**Affiliations:** † Department of Biotherapy, State Key Laboratory of Biotherapy and Cancer Center, Laboratory of Neuro-system and Multimorbidity, West China Hospital, 12530 Sichuan University, Chengdu 610041, Sichuan, China; ‡ Department of Anesthesiology, National Clinical Research Center for Geriatrics, West China Hospital, Sichuan University, Chengdu 610041, Sichuan, China; § Frontiers Medical Center, Tianfu Jincheng Laboratory, Chengdu 610212, Sichuan, China; ∥ School of Food Science and Technology, 66374 Jiangnan University, Wuxi 214122, Jiangsu, China

## Abstract

Sirtuin 3 (SIRT3), a pivotal mitochondrial deacetylase, plays a critical role in restoring mitochondrial function, particularly through the activation of autophagy. Despite its promise as a cardioprotective target, developing SIRT3 activators and their therapeutic applications remains challenging. Here, we report the identification of **SKLB-11A**, a SIRT3 activator with submicromolar affinity and high efficacy. Structural and mutagenesis analyses revealed a unique allosteric site for **SKLB-11A** in SIRT3, where a conformational change in Leu298 drives its potent activation. Subsequent studies demonstrated that **SKLB-11A** drives autophagy/mitophagy signaling pathways, effectively preventing mitochondrial dysfunction, and improving cardiac dysfunction in both doxorubicin (Dox)-induced cardiotoxicity and myocardial ischemia/reperfusion (I/R) models. Collectively, our data highlight the potential of pharmacological SIRT3 activation as an effective therapeutic strategy for cardioprotection. **SKLB-11A**, as a first-in-class SIRT3 allosteric activator with a distinct binding mode, not only offers a valuable tool for exploring the physiological and pathological roles of SIRT3 deacetylation but also holds promise for the development of targeted cardioprotective therapies.

## Graphical abstract

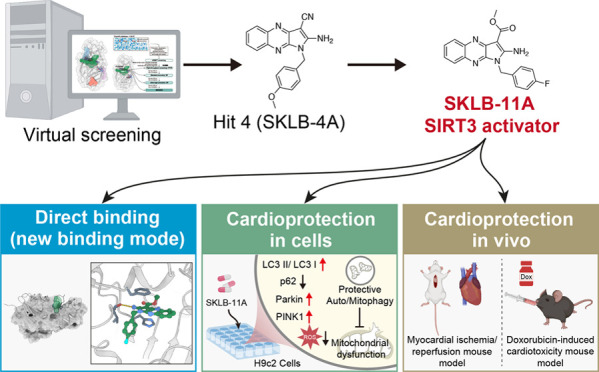

## Introduction

Sirtuins are highly conserved NAD^+^-dependent enzymes, and their dysregulation has been implicated in metabolic, cardiovascular, and neurological diseases.^1^ In mammals, the sirtuin family comprises seven members (SIRT1–7),^2,3^ with SIRT3 serving as a mitochondrial health sensor.^4^ It promotes the deacetylation of numerous proteins in the mitochondrial matrix, thereby restoring normal membrane potential.^5^ SIRT3-mediated deacetylation also activated manganese superoxide dismutase (MnSOD), which plays a protective role in mitochondrial oxidative stress.^6^ Compared to other members of the SIRT family, SIRT3′s deacetylation activity plays a more distinct role in regulating mitochondrial function.^7^ Notably, increasing evidence highlights the significant cardioprotective role of SIRT3.^8,9^ It has been demonstrated that SIRT3 expression mitigates Dox-induced dilated cardiomyopathy by preventing acetylation of mitochondrial proteins.^10^ Moreover, inhibition of SIRT3 was a key factor in cardiovascular damage caused by persistent hepatic steatosis and inflammation under high salt conditions.^11^ In contrast, restoration of cardiac SIRT3 expression via adeno-associated virus in FGF21 gene knockout mice with diabetes was sufficient to improve their exercise responsiveness, thereby alleviating mitochondrial dysfunction and dilated cardiomyopathy.^12^ These findings suggest that SIRT3 activation is a promising therapeutic strategy to restore mitochondrial function and reduce myocardial injury.^13^ Thus, pharmacological targeting of SIRT3 activation through small molecules may provide a foundation for the development of cardioprotective therapies.^14,15^

Over the years, several SIRT activators have been developed, some exhibiting broad Sirtuin activity, while others show isoform-specific selectivity. Most of these activators have served as valuable probes for exploring the biology of sirtuins, though they also hold potential as starting points for drug development.^16–18^ Recent efforts have resulted in the development of SIRT1, SIRT3, SIRT5, SIRT6, and SIRT7 activators.^14,15,19–26^ Resveratrol is the first allosteric activator of SIRT1 described in the literature,^27^ sparking the development of STACs,^28^ among which SRT2104 showed promising results in phase I/II clinical trials.^29^ The combination of molecular dynamics simulations with high-throughput docking has facilitated the identification of allosteric activators for SIRT1, laying the groundwork for the design of novel SIRT3 activators.^30^ Compound 33c, designed based on the SIRT3 structure, has been investigated for treating triple-negative breast cancer.^31^ Steegborn et al. identified and characterized 1,4-dihydropyridine (1,4-DHP)-based compounds as potent SIRT3 or SIRT5 activators. Notably, compound 31 exhibited specific SIRT3 activation. These activators have demonstrated antiproliferative effects in breast and thyroid cancer cell lines.^32^ Their findings provide a valuable scaffold for developing SIRT3-specific therapeutic agents. The SIRT6 allosteric activator MDL-800, identified using allosite tools, has shown therapeutic potential in liver cancer.^33^ Steegborn et al. synthesized and screened pyrrolo­[1,2-*a*]­quinoxaline derivatives, identifying the first synthetic SIRT6 activators, which effectively activate SIRT6-dependent deacetylation of peptide substrates and complete nucleosomes.^19^ Despite these advances, the development of SIRT3 activators remains challenging. Currently, most known SIRT3 activators are derived from natural products, such as honokiol.^34^ Other compounds, including adjudin, a well-characterized Cl^–^-channel blocker extensively studied in preclinical and clinical settings as a male contraceptive, have also been reported to protect cochlear hair cells from gentamicin-induced hearing loss in rodent models via the SIRT3-ROS pathway.^35^ Dexmedetomidine has been shown to alleviate cardiac ischemia/reperfusion injury by enhancing autophagy through AMPK/SIRT3 pathway activation.^36^ Furthermore, in an atherosclerosis mouse model, melatonin was found to activate SIRT3–FOXO3a–Parkin-mediated mitophagy, thereby suppressing inflammation and atherosclerotic progression.^37^ Collectively, these compounds have been implicated in modulating SIRT3-related pathways; however, the binding modes based on crystal structures still warrant further exploration. This highlights the limited understanding of the precise interaction patterns between these activators and the protein, as well as a deficiency in target specificity and a lack of structural basis for protein-small molecule complexes.^38^ More importantly, no SIRT3 activators with a distinct binding mode have been developed for cardioprotection to date. Therefore, continued research is essential to explore and optimize SIRT3-targeted therapies for cardioprotection.

The human SIRT3 protein contains a mitochondrial targeting sequence (MTS), an unstructured region and a conserved enzymatic core, which includes an NAD^+^ binding domain and a metal-binding motif.^39^ Sirtuins share a similar deacylation mechanism,^23^ with residue H248 being essential for their catalytic activity.^40^ In this context, we hypothesized that it might be feasible to engineer allosteric SIRT3 activators by leveraging structural design strategies. Specifically, we sought to identify novel chemical entities capable of binding to distinct allosteric sites on SIRT3. Our dual objective was to (1) achieve effective cardioprotection by modulating SIRT3 activity with these activators and (2) ensure acceptable selectivity for SIRT3 over other sirtuin family members. By doing so, we aimed to facilitate more precise and potent cardioprotective interventions that are grounded in the targeted activation of SIRT3.

Here, we employed a hybrid strategy that integrates computational simulations with experimental validation to systematically identify potential activators of SIRT3, leading to the discovery of **SKLB-11A**. **SKLB-11A** showed remarkable efficacy in mouse models of doxorubicin (Dox)-induced cardiotoxicity and myocardial ischemia/reperfusion (I/R) injury, along with favorable pharmacokinetics and safety. To our knowledge, **SKLB-11A** is the first small-molecule allosteric SIRT3 activator with a distinct binding mode, providing a solid foundation for the development of SIRT3-targeted cardioprotective therapies.

## Results

### SKLB-11A Is Identified as a Potential Activator of SIRT3

Considering the challenges of pharmacologically activating SIRT3, we designed a hybrid strategy that combines computational and experimental approaches to identify potential SIRT3 activators. Initially, the SiteMap module in Schrödinger was employed to predict potential binding sites in SIRT3, identifying four candidate sites, with site 1 exhibiting the highest score of 1.055 (Figure 1A, Supplementary Table S1). We subsequently docked over 1.6 million compounds virtually into site 1. A small subset of the ChemDiv database was then preprocessed using Schrödinger’s LigPrep, and an initial ADMET filter was applied to remove compounds that did not meet Lipinski’s rules or contained reactive fragments. Following this, high-throughput virtual screening (HTVS) was conducted, retaining the top 10% of compounds for standard precision (SP) docking. From the SP docking results, we further assessed the top 10% using high precision (XP) docking, followed by MMGBSA calculations to estimate the binding free energy (Figure 1B). Based on top-ranked SIRT3-compound binding models, five hit compounds were selected and purchased for further analysis (Supplementary Figure S1). To assess the deacetylation activity of these compounds on SIRT3, we employed a Fluor de Lys (FDL) assay. Among the five hit compounds, hit 4 (**SKLB-4A**) exhibited the highest deacetylase activity, showing an effect of 118.19 ± 0.79% at 100 μM, compared to the control group. To further evaluate its cellular activity, an *in vitro* Dox-induced cardiotoxicity model was employed. Pretreatment with **SKLB-4A** enhanced H9c2 cell viability to 65.31 ± 4.54%, compared to a decrease to 61.93 ± 0.51% following Dox treatment (Figure 1B).

**Figure 1:**
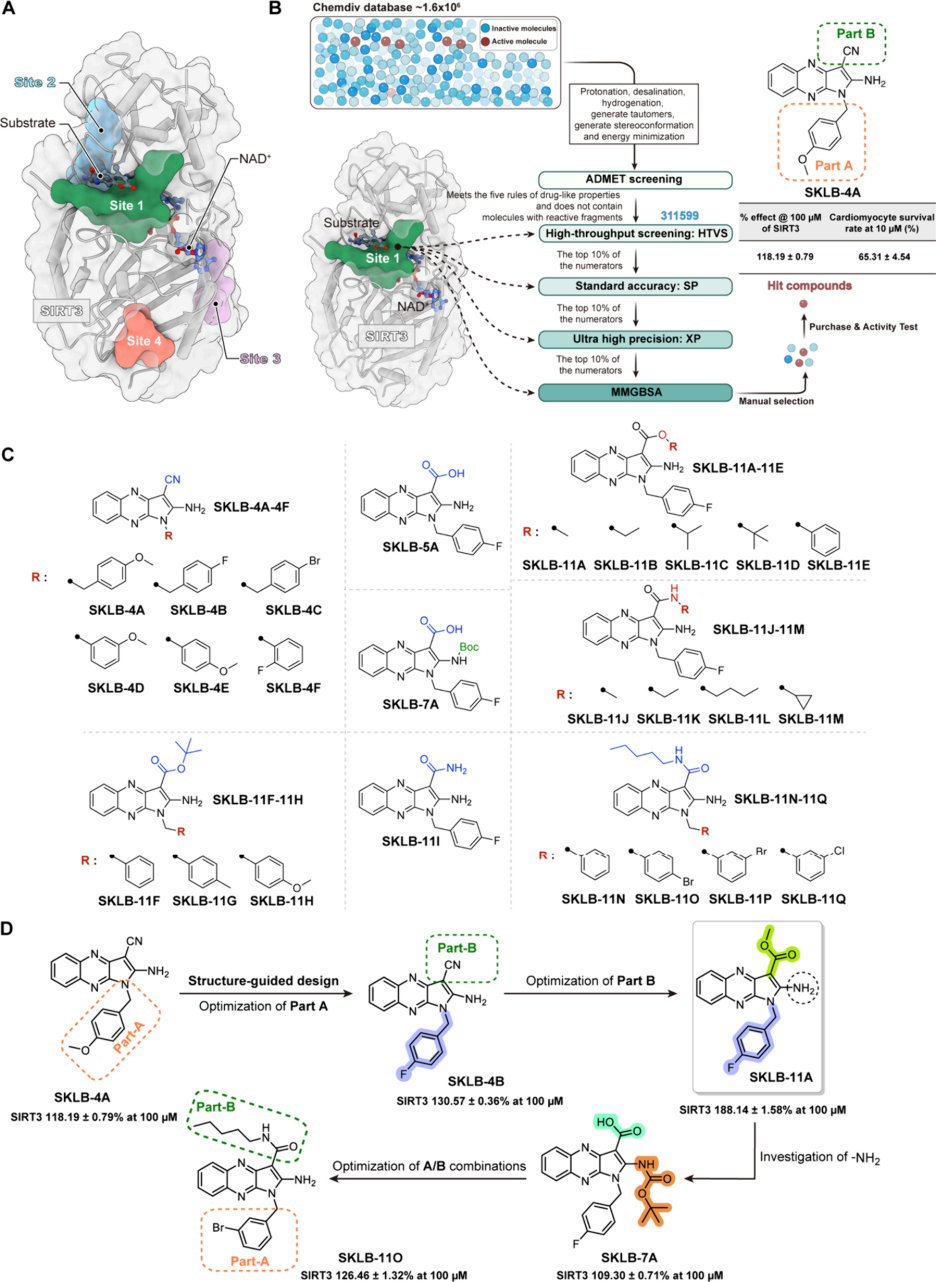
Structure-based screening of small-molecule activators of SIRT3 and identifies **SKLB-11A**. (A) Predicted molecular binding sites in SIRT3 by the SiteMap module of Schrödinger (PDB: 8 V2N). (B) Schematic describing the drug discovery campaign deployed to discover the lead pyrrolo­[2,3-*b*]­quinoxaline series. (C) Structures of pyrrolo­[2,3-*b*]­quinoxaline derivatives. (D) The optimization process of SIRT3 activators.

To further enhance the intracellular and extracellular bioactivity of **SKLB-4A**, we utilized it as a lead scaffold to optimize novel SIRT3 activators (Supplementary Scheme S1, Table S2). Docking analysis of **SKLB-4A** with SIRT3 revealed that the pyrrolo­[2,3-*b*]­quinoline structure in **SKLB-4A** forms stable π-π stacking interactions with Phe180 and His248 in the binding pocket, which are crucial for maintaining SIRT3 deacetylation activity. Additionally, significant optimization potential exists around the benzyl, cyano, and amino groups (Supplementary Figure S2). Next, based on the docking analysis of **SKLB-4A** with SIRT3, compound optimization will primarily focus on Part A and Part B, as detailed in the following parts. First, we explored different aromatic rings for the part A and obtained the first series of compounds **SKLB-4B-F**. The SIRT3 deacetylation activity and cardioprotective activity of these compounds indicated that the phenyl-substituted derivative (**SKLB-4D-F**) reduced SIRT3 deacetylation activity to varying degrees. This indicated the existence of methylene between the part A benzene ring and the parent nuclear scaffold, making the appropriate spatial distance between the two have an important effect on activity. In particular, replacing the methoxy group with a fluorine atom (**SKLB-4B**) likely enhances the interactions between the compound and Leu298/Gly295, resulting in a significant increase in SIRT3 deacetylation activity. Next, we further optimized the cyano group in part B by introducing ester or amide moieties, resulting in derivatives **SKLB-11A-E** and **SKLB-11J-M**. The results showed that the introduction of alkyl esters in part B maintained or enhanced the activity of compounds (**SKLB-11A**, **SKLB-11B**, and **SKLB-11D**). Among them, **SKLB-11A** had the strongest SIRT3 deacetylation activity and cardioprotective activity (deacetylation rate of 188.14 ± 1.58% and cell survival rate of 78.88 ± 0.65%). The introduction of a boc group at the amino significantly reduced SIRT3 deacetylation activity (**SKLB-7A**), indicating that the hydrogen bond interaction between the compound and Glu325 plays a crucial role in maintaining the fit within the SIRT3 binding pocket. Subsequently, further investigation of different substituent combinations in parts A/B resulted in varying degrees of reduced activity (**SKLB-11F-H and SKLB-11N-Q**).

In summary, the introduction of electrophilic groups on the benzyl moiety in Part A significantly enhances SIRT3 deacetylation activity, with the fluorinated benzyl derivative demonstrating the most potent effect. In Part B, the incorporation of ester or amide groups can improve activity, while the presence of a free amine is critical for maintaining SIRT3 deacetylation activity. Conversely, Boc-protected amines substantially reduce activity. These findings highlight the importance of specific structural modifications in optimizing SIRT3 activation potency. Ultimately, compound **SKLB-11A** was selected as the lead compound for subsequent biological evaluation due to its highest activation potency of SIRT3 deacetylation, and cardioprotective activity (Figure 1C, D).

### SKLB-11A Directly Targets and Enhances the Deacetylase Activity of SIRT3

The FDL assay showed that **SKLB-11A** enhanced SIRT3 deacetylation activity, with an EC_50_ of 21.95 ± 1.57 μM (Figure 2A). To further confirm the reproducibility of this effect, we utilized liquid chromatograph mass spectrometer with the substrate GELLEAIK-Ac (Figure 2B). Both assays corroborated that **SKLB-11A** significantly increased the catalytic efficiency of SIRT3 deacetylation. Importantly, the addition of the SIRT3 inhibitor 3-TYP yielded the opposite effect, further validating **SKLB-11A** as a SIRT3 activator. To further evaluate the SIRT3 activation potency of **SKLB-11A**, we compared its activity with other SIRT3 activators. In the FDL assay, **SKLB-11A** exhibited better SIRT3 activation compared to honokiol, resveratrol, and adjudin (Figure 2C). To further substantiate these observations, we performed coupled enzymatic assays utilizing fluorophore-free peptide substrates,^41^ which produced comparable results, supporting the robust and reproducible SIRT3 activation conferred by **SKLB-11A** (Supplementary Figure S3). Previous studies have shown that SIRT3 is closely associated with oxidative stress and plays a key role in cardiovascular diseases.^42^ To assess the activation of SIRT3 deacetylation by **SKLB-11A** in H9c2 cells, we examined the acetylation status of mitochondrial proteins following **SKLB-11A** treatment. We found that **SKLB-11A** induced deacetylation at K122 and K68, resulting in enhanced MnSOD enzymatic activity (Figure 2D). Notably, while the positive control compounds exhibited limited SIRT3 activation in enzymatic assays, they showed pronounced deacetylation effects on MnSOD-K122 and K68 in cellular contexts, potentially due to complex regulatory mechanisms within the cell (Supplementary Figure S4). Next, we investigated whether this activation of SIRT3 was due to an increase in cellular SIRT3 levels after **SKLB-11A** treatment. The results indicated that **SKLB-11A** did not alter SIRT3 protein levels but significantly enhanced SIRT3 activity (Figure 2D). Thus, **SKLB-11A** activates endogenous SIRT3 deacetylation in H9c2 cells.

**Figure 2:**
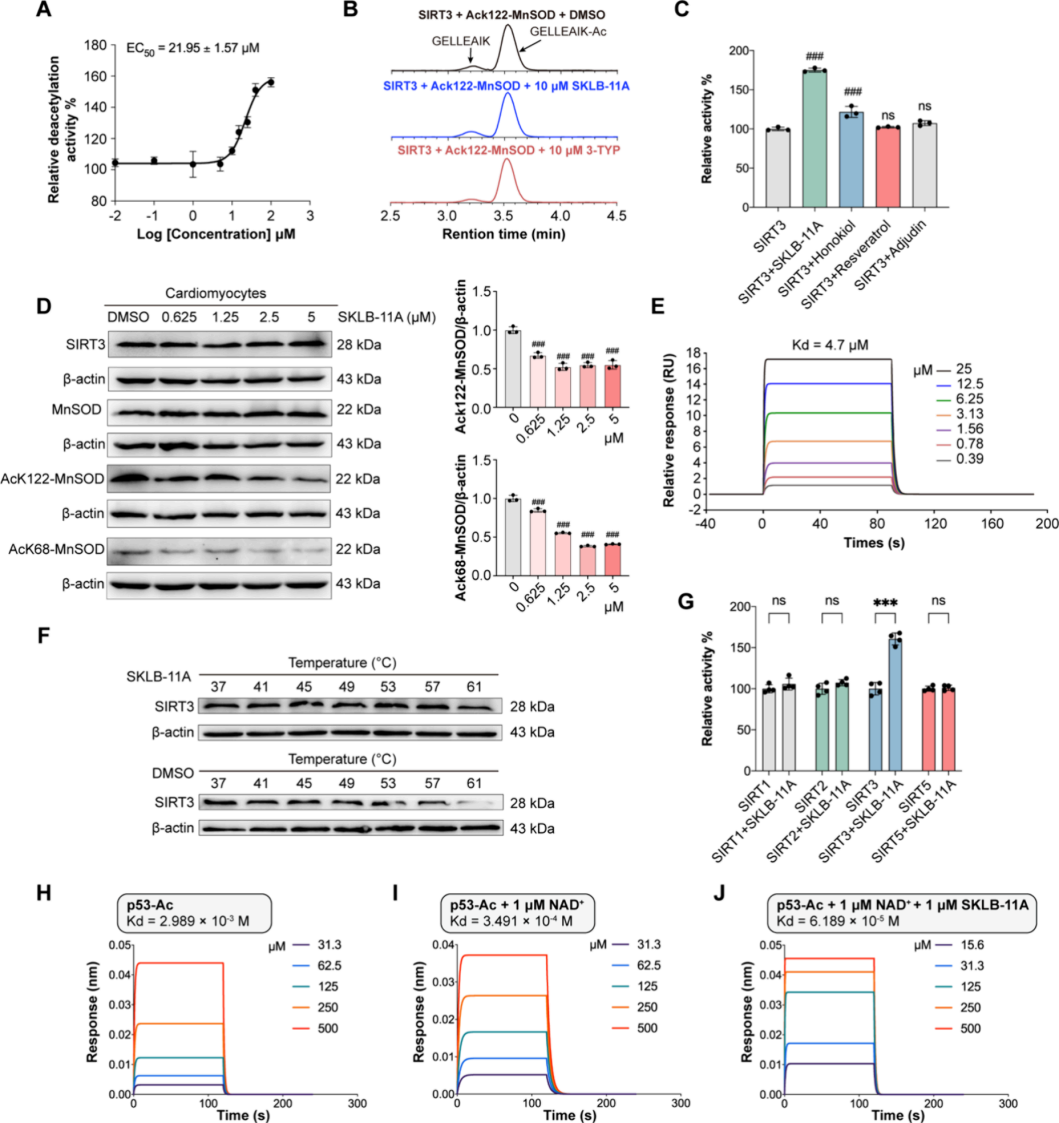
**SKLB-11A** directly targets and activates SIRT3. (A) Dose-dependent effects of **SKLB-11A** on SIRT3, determined by fluorimetric activity assays. (B) Mass spectrometry (MS)-based deacetylation assay in the absence and presence of **SKLB-11A** (100 μM) or the SIRT3 inhibitor 3-TYP, which confirms direct activation or inhibition of SIRT3. (C) Effects of **SKLB-11A**, honokiol, resveratrol, and adjudin on SIRT3-dependent peptide deacetylation by fluorimetric activity assays. (D) Western blot analysis of MnSOD, SIRT3, and acetylated MnSOD (at K68 and K122) in H9c2 cells treated with **SKLB-11A** for 24 h. β-actin, loading control. Data are presented as mean ± SD, *n* = 3. ^#^ *p* < 0.05, ^##^ *p* < 0.01, ^###^ *p* < 0.001 vs control group. (E) SPR analysis of the binding interaction between **SKLB-11A** and SIRT3. (F) CETSA assay assessed the thermal stability of SIRT3 in H9c2 cells treated with **SKLB-11A**. (G) Selectivity of **SKLB-11A** (100 μM) among human sirtuin isoforms SIRT1, 2, 3, and 5. SIRT5 was tested with succinylated substrate, all other isoforms with acetylated peptides. (H) The binding affinity of p53-Ac to SIRT3 was determined by biolayer interferometry (BLI) assays. (I) The binding affinity of p53-Ac to SIRT3 in the presence of 1 μM NAD^+^. (J) The binding affinity of p53-Ac to SIRT3 in the presence of 1 μM NAD^+^ and 1 μM **SKLB-11A**.

Additionally, we used surface plasmon resonance (SPR) to measure the binding of **SKLB-11A** to immobilized SIRT3. **SKLB-11A** exhibited concentration-dependent binding to SIRT3, with a *K*_d_ value of 4.7 μM (Figure 2E). Cellular thermal shift assays (CETSA) further validated that **SKLB-11A** effectively binds to SIRT3 (Figure 2F). We next evaluated the selectivity of **SKLB-11A** for different SIRT family members. We found that **SKLB-11A** showed no binding to SIRT5 or SIRT6. Although **SKLB-11A** displayed some binding affinity for SIRT1 and SIRT2, the *K*_d_ values exceeded 200 μM, approximately 40 times lower than its affinity for SIRT3 (Supplementary Figure S5). Furthermore, **SKLB-11A** had little effect on the thermal stability of SIRT1, SIRT2 and SIRT5 (Supplementary Figure S6). We next examined the functional specificity of **SKLB-11A** using coupled enzymatic assays with isoform-specific, physiologically relevant substrates: acetylated p53-K381 for SIRT1, acetylated α-tubulin-K25 for SIRT2, acetylated MnSOD-K122 for SIRT3, and succinylated CPS1-K537 for SIRT5 (Supplementary Table S3).^32^ **SKLB-11A** showed no statistically significant effects on basal SIRT1, SIRT2, and SIRT5 deacetylation or desuccinylation activities. In contrast, **SKLB-11A** exhibited pronounced activation of SIRT3 (Figure 2G). Using Schrödinger molecular docking simulations, we assessed the binding interactions of **SKLB-11A** with SIRT isoforms. The results revealed that **SKLB-11A** exhibited different binding modes across these isoforms. In its predominant conformation, **SKLB-11A** exhibited an intermolecular binding energy of – 6.042 kcal/mol with SIRT3, slightly stronger than with other SIRT isoforms (Supplementary Figure S7). Collectively, these results indicated that **SKLB-11A** is a selective activator of SIRT3.

To elucidate the underlying mechanism, biolayer interferometry was employed to examine the effect of **SKLB-11A** on the real-time interaction between SIRT3 and its substrate.^43^ When SIRT3 bound to acetylated p53 substrate (p53-Ac), the *K*_d_ was 2.989 mM (Figure 2H). Upon the addition of the coenzyme NAD^+^ (1 μM), the binding affinity of p53-Ac to SIRT3 increased, with a *K*_d_ of 349.1 μM (Figure 2I). Moreover, the addition of **SKLB-11A** (1 μM) to the SIRT3 reaction system further enhanced the affinity of p53-Ac for SIRT3, resulting in a *K*_d_ of 61.89 μM (Figure 2J). Taken together, these results demonstrated that **SKLB-11A** binds to SIRT3 with high affinity and enhances its interaction with the p53-Ac substrate.

### SKLB-11A Induces a Conformational Flip in Leucine 298 of SIRT3

In an effort to uncover the molecular interplay between **SKLB-11A** and SIRT3, we analyzed the structure of the SIRT3-**SKLB-11A** complex using X-ray crystallography at a resolution of 2.49 Å (Supplementary Table S4). The structural data revealed that the pyrrole ring of **SKLB-11A** inserts deeply into the cleft between two structural domains (Figure 3A, B), where it forms a π-π stacking interaction with His248, a critical residue essential for deacetylase activity and conserved across the SIRT family (SIRT1–7).^44^ Furthermore, **SKLB-11A** formed a hydrogen bond with Val292 and tightly positioned between Phe180 and His248 (Figure 3C). Intriguingly, a significant conformational shift was observed in the side chain of Leu298, which deviated substantially from its orientation in previously reported SIRT3 structures (Figure 3D). These findings suggest that **SKLB-11A** may modulate SIRT3 allosterically through an entirely novel mechanism.

**Figure 3:**
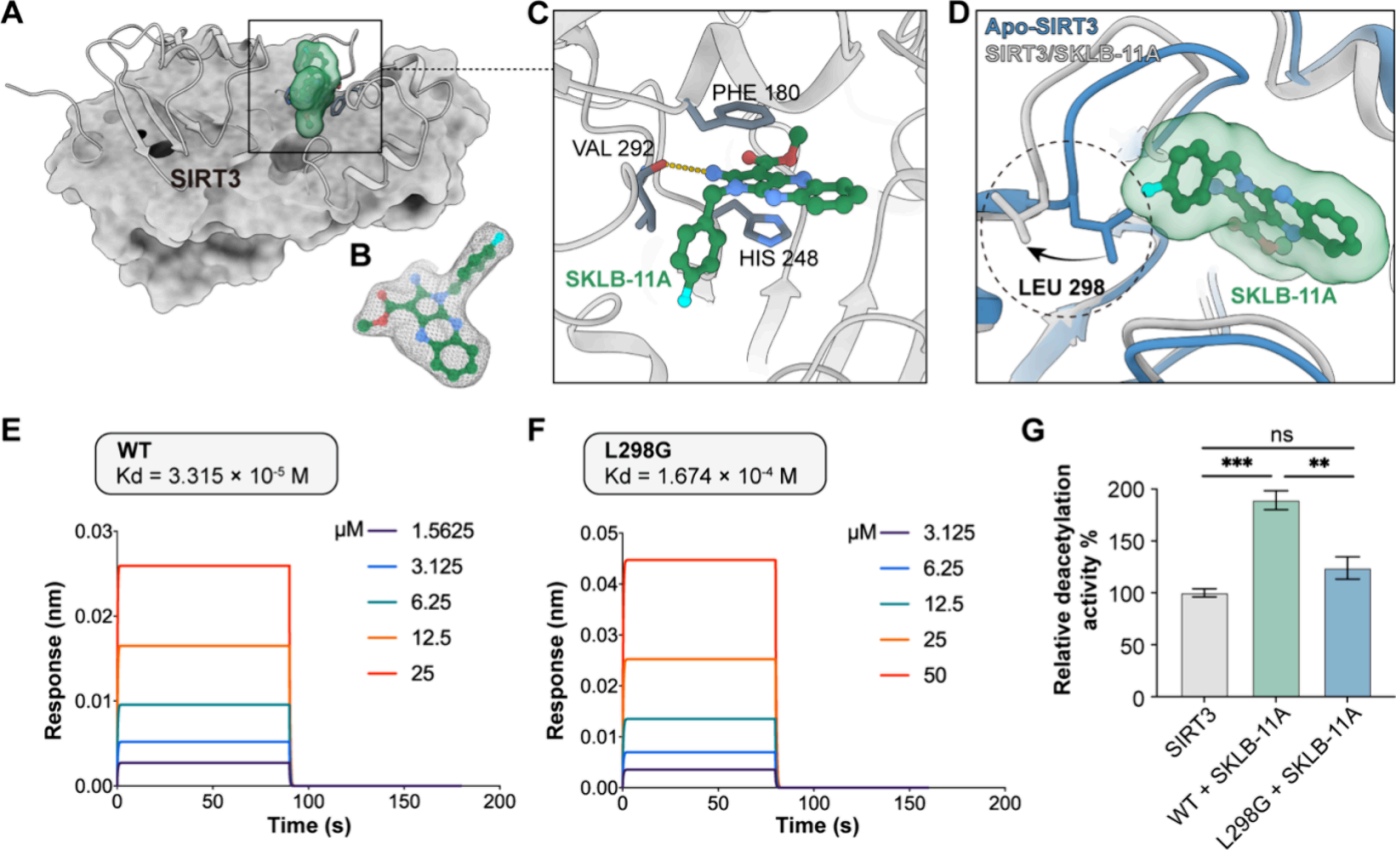
**SKLB-11A** induces conformational flipping of Leu298. (A–B) The cocrystal structure of the **SKLB-11A** and SIRT3 complex. (C) Key amino acids involved in the binding of **SKLB-11A** to SIRT3. (D) Overlay of the reported SIRT3 structures (in blue, PDB ID: 3GLS), with the SIRT3–**SKLB-11A** complex structure (in gray) complex, highlighting the amino acids Leu 298. (E) Biolayer interferometry (BLI) analysis of interactions between **SKLB-11A** and wild-type SIRT3. (F) BLI analysis of the interaction between **SKLB-11A** and mutant SIRT3. (G) Effects of **SKLB-11A** on the deacetylation activity of wild-type and mutant SIRT3 proteins, assessed using fluorimetric activity assays. Data are represented as mean ± SD in triplicate. *p < 0.05, **p < 0.01, and ***p < 0.001.

To further evaluate the functional consequences of side-chain reorientation, we substituted Leu298 with glycine. This mutation led to a marked reduction in enzymatic activity, as revealed by activity assays (Figure 3G). Biochemical analysis further revealed a diminished binding affinity between **SKLB-11A** and the L298G mutant, with the dissociation constant increasing from 33.15 μM for the wild-type protein to 167.4 μM for the mutant (Figure 3E, F). Collectively, these findings highlight the essential role of the Leu298 side chain in stabilizing the interaction between **SKLB-11A** and SIRT3 and preserving the enzyme’s deacetylase activity.

### SKLB-11A Ameliorates Dox-Induced Mitochondrial Damage and Promotes SIRT3-Mediated Autophagy in Cells

And then, we examined the effects of **SKLB-11A** on SIRT3-dependent cardioprotection by generating stable SIRT3-knockdown cell lines using shRNA (Figure 4A). Cell viability assays revealed that the cardioprotective effects of **SKLB-11A** were significantly diminished in H9c2 cells with reduced SIRT3 expression (Figure 4B). Similarly, treatment with the SIRT3 inhibitor 3-TYP also attenuated the efficacy of **SKLB-11A** (Figure 4C). Furthermore, we found that **SKLB-11A** exhibited slightly better protective effects on H9c2 cells compared to the positive control compounds (Supplementary Figure S8). In short, these results underscore the reliance of **SKLB-11A** on SIRT3 for its cardioprotective function.

**Figure 4:**
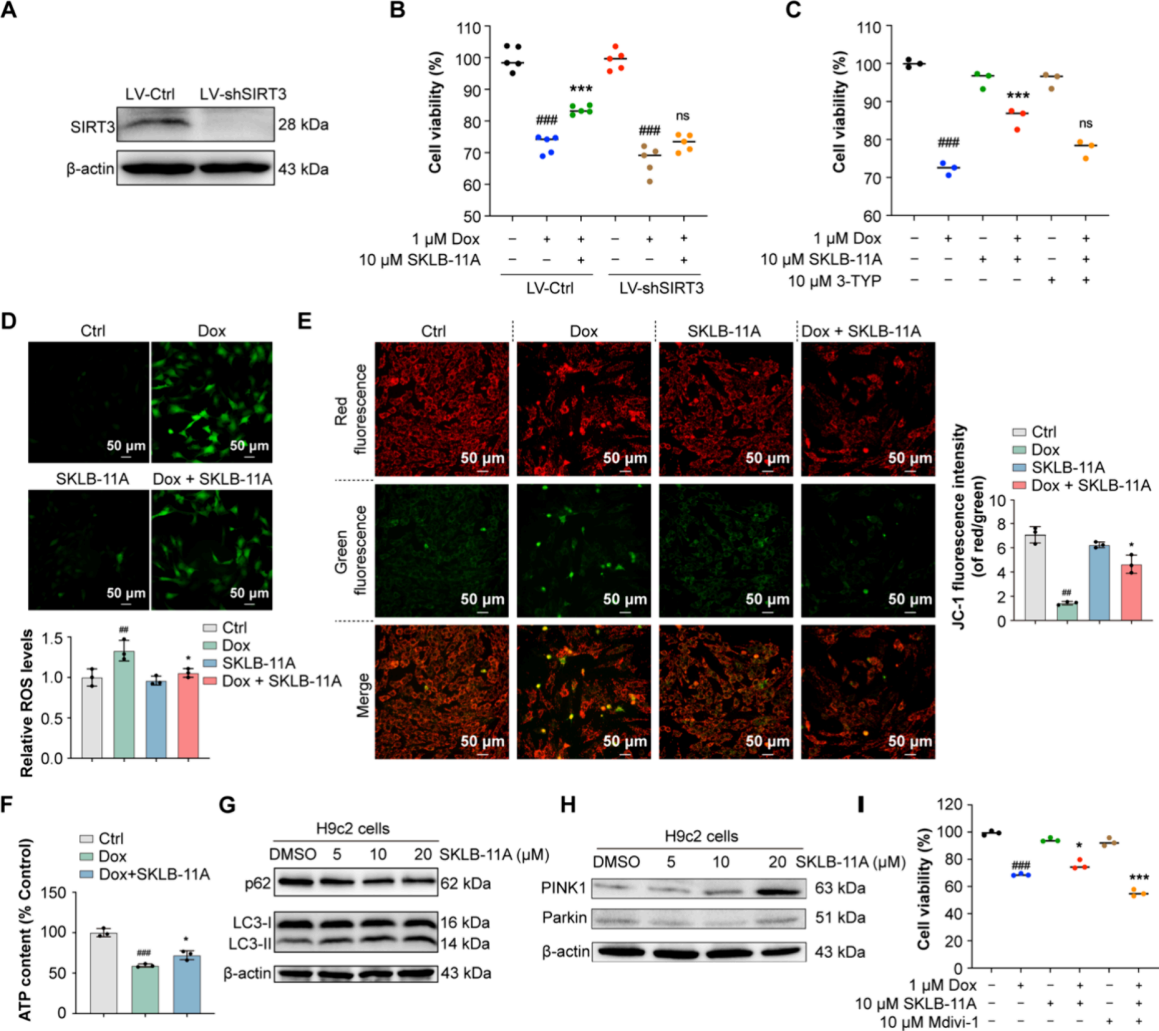
**SKLB-11A** improves mitochondrial function and induces autophagy/mitophagy in H9c2 cells. (A–B) Effects of SIRT3 knockdown on the therapeutic efficacy of **SKLB-11A** in H9c2 cells. (C) Effects of the SIRT3 inhibitor 3-TYP on the therapeutic effect of **SKLB-11A** in Dox-treated H9c2 cells. Data are presented as mean ± SD, *n* = 3. (D) Detection of intracellular ROS in cells stained with DCFH-DA, observed using fluorescence microscopy. (E) Effects of **SKLB-11A** on mitochondrial membrane potential reduction induced by Dox. (F) Changes in ATP content in Dox-treated H9c2 cells following treatment with **SKLB-11A**. (G) Western blot analysis of p62 and LC3 protein expression in H9c2 cells treated with **SKLB-11A** for 24 h. β-actin was used as a loading control. (H) Western blot analysis of PINK1 and Parkin protein expression in H9c2 cells treated with **SKLB-11A** for 24 h. β-actin was used as a loading control. (I) The addition of mdivi-1 further confirms the role of mitophagy in the therapeutic effects of **SKLB-11A**. Data are presented as mean ± SD, *n* = 3. *p < 0.05, **p < 0.01, and ***p < 0.001, vs Dox treatment group, ^#^ *p* < 0.05, ^##^ *p* < 0.01, ^###^ *p* < 0.001 vs control group.

Given the critical role of reactive oxygen species (ROS) in cardiac oxidative stress and myocardial injury, we next investigated whether **SKLB-11A** could mitigate ROS production in H9c2 cells.^45^ The results showed that Dox significantly increased ROS levels, while **SKLB-11A** pretreatment effectively suppressed this increase, with a slightly stronger effect than the positive control compounds (Figure 4D, Supplementary Figure S9). As mitochondrial dysfunction is closely linked to ROS formation and the collapse of mitochondrial membrane potential (MMP), we further assessed MMP.^46^ Dox treatment caused substantial MMP dissipation, which was notably preserved by **SKLB-11A** (Figure 4E). As ATP is the primary energy source for cardiomyocytes, we next evaluated cellular ATP levels to assess the energy state.^47^ Consistent with the cytotoxic effects of Dox, a significant reduction in ATP content was observed in H9c2 cells, indicating impaired energy metabolism. In contrast, we found that both the positive control compounds and **SKLB-11A** were capable of increasing ATP levels (Figure 4F, Supplementary Figure S10). Collectively, these findings emphasize that **SKLB-11A** can alleviate Dox-induced mitochondrial damage, demonstrating its ability to restore mitochondrial function.

To investigate the mechanisms by which **SKLB-11A** alleviates mitochondrial dysfunction, we focused on its potential role in modulating mitophagy, a critical process in mitochondrial quality control (QMC) that maintains mitochondrial function following injury.^48,49^ Our group previously developed compound 33i, which enhances protective autophagy by targeting ULK1, thereby ameliorating MPTP-induced motor dysfunction and dopaminergic neuronal loss.^50^ Thus, we sought to determine whether **SKLB-11A** could also trigger this protective cellular mechanism. The results showed that **SKLB-11A** treatment increased LC3-II expression and reduced p62 levels, indicating enhanced autophagic flux (Figure 4G, Supplementary Figure S11). In addition, **SKLB-11A** also increased the expression of PINK1 and Parkin, key initiators of mitophagy (Figure 4H, Supplementary Figure S11),^51^ and promoted colocalization of mitochondria with Parkin and LC3-II (Supplementary Figure S12). In contrast, 3-TYP treatment reduced LC3-II localization, suggesting an inhibitory effect on autophagy (Supplementary Figure S12). This result was further supported by the accumulation of MDC fluorescence signal, which labeled the cellular autophagic vacuoles (Supplementary Figure S13). To confirm that the protective effects of **SKLB-11A** are mediated through mitophagy, we evaluated cell viability in the presence of Mdivi-1, a mitophagy inhibitor.^52^ The results indicated that Mdivi-1 attenuated the cardioprotective effects of **SKLB-11A** (Figure 4I). These findings suggest that **SKLB-11A** induces protective autophagy/mitophagy, potentially facilitating the removal of damaged mitochondria.

### SKLB-11A Repressed Dox-Induced Cardiac Dysfunction in Vivo

Encouraged by these positive results, we initially established a Dox-induced cardiotoxicity model to assess the cardioprotective effects of **SKLB-11A** (Figure 5A). Pretreatment with **SKLB-11A** (10 or 20 mg/kg, ig) significantly restored left ventricular ejection fraction (EF) and fractional shortening (FS) in a dose-dependent manner, mitigating the cardiac dysfunction induced by Dox (Figure 5B). Moreover, **SKLB-11A** partially reversed Dox-induced weight loss (Supplementary Figure S14A) and reduced serum markers of myocardial injury, including LDH, CK-MB, and BNP (Figure 5C–E). Histological analysis revealed that **SKLB-11A** preserved myocardial structure and alleviated pathological damage, such as disrupted myocardial architecture and myofibril necrosis, which were prominent in the Dox group (Supplementary Figure S14B). Importantly, **SKLB-11A** demonstrated no apparent toxicity at effective doses, as evidenced by preserved organ histology in the heart, liver, spleen, lungs, and kidneys (Supplementary Figure S15).

**Figure 5:**
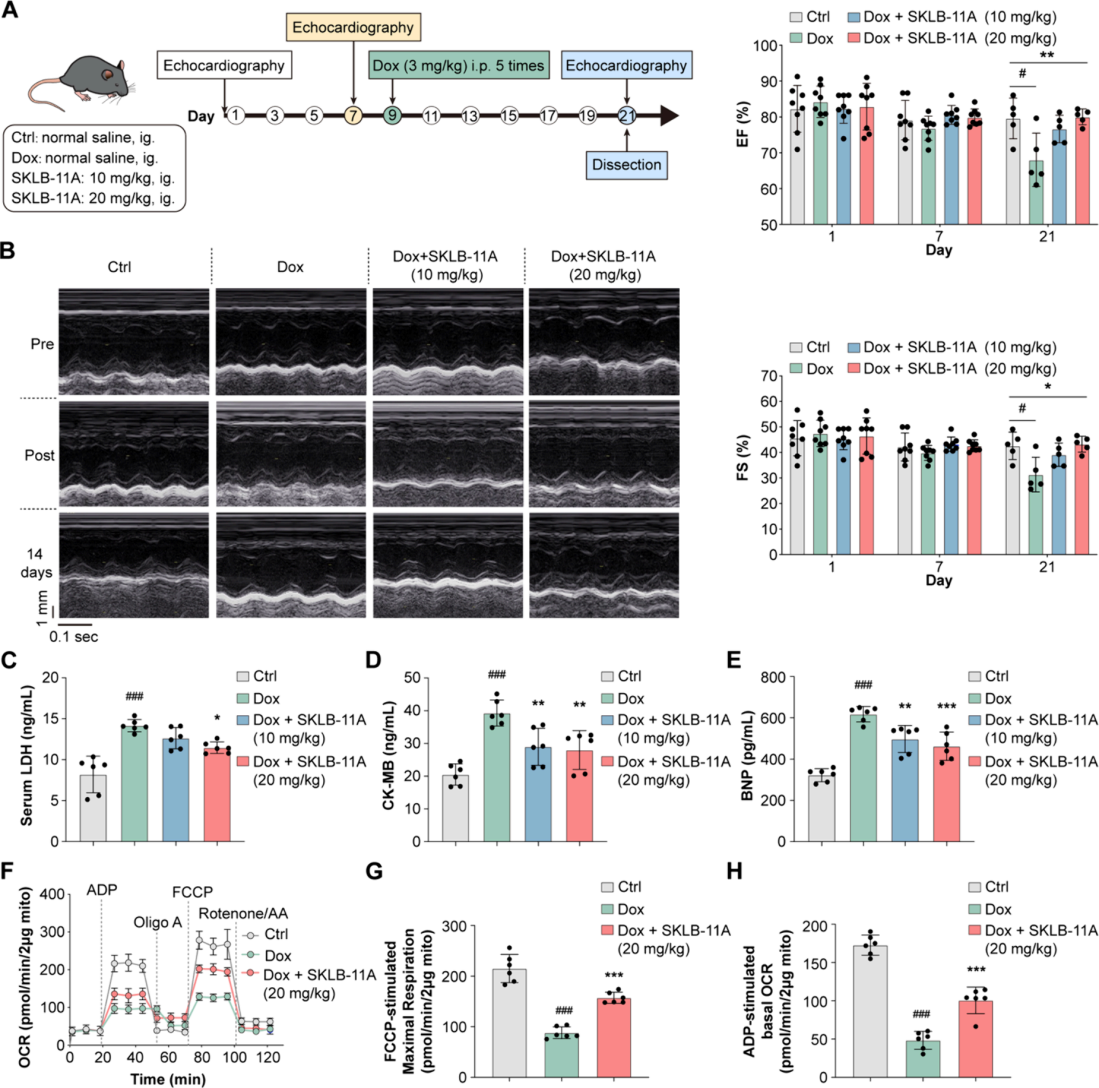
The cardioprotective effects of **SKLB-11A** in Dox-induced cardiotoxicity models *in vivo*. (A) Schematic illustration of the **SKLB-11A**’s protective activity evaluation *in vivo*. (B) The cardiac health status of mice in each group was evaluated by echocardiography, Data was presented as mean ± SD, *n* = 5–8. (C–E) Serum concentration of LDH, CK-MB, and BNP. Data are presented as mean ± SD, *n* = 6. (F–H) Effects of **SKLB-11A** on mitochondrial respiration in the hearts of mice treated with Dox, including oxygen consumption rate profile plot, maximal respiration and basal respiration. Data are presented as mean ± SD, *n* = 6, *p < 0.05, **p < 0.01, and ***p < 0.001, vs Dox treatment group, ^#^ *p* < 0.05, ^##^ *p* < 0.01, ^###^ *p* < 0.001 vs control group.

Then, we investigated the mechanisms underlying these effects by examining autophagy-related protein expression and mitochondrial function. Western blot analysis revealed that **SKLB-11A** ameliorated Dox-induced autophagy dysregulation, particularly by normalizing p62, LC3-II, and Parkin levels in the high-dose group (Supplementary Figure S16A). Immunohistochemistry analysis further confirmed consistent alterations in the expression of p62, Parkin, and SIRT3 expression (Supplementary Figure S16B). Additionally, **SKLB-11A** restored mitochondrial respiration, as indicated by increased basal respiration andmaximal respiration in heart tissues from treated mice (Figure 5F–H). Overall, these results suggest that **SKLB-11A** protects against Dox-induced cardiotoxicity by modulating autophagy and restoring mitochondrial function, providing effective cardioprotection with no observable toxicity.

### SKLB-11A Treatment Alleviated I/R-Induced Myocardial Injury in Vitro and in Vivo

SIRT3 plays a crucial role in the protective response during myocardial ischemia/reperfusion (I/R).^53^ In this context, we investigated the therapeutic potential of **SKLB-11A**. To evaluate its protective effects against myocardial damage following I/R injury, we first employed an oxygen-glucose deprivation/reperfusion (OGD/R) model to simulate I/R injury *in vitro*.^54^ Seahorse analysis revealed that OGD/R treatment significantly reduced mitochondrial oxygen consumption capacity (Supplementary Figure S17A–D), while Mito-SOX Red staining detected a marked increase in ROS levels, indicative of mitochondrial dysfunction (Supplementary Figure S17E–F). Notably, pretreatment with **SKLB-11A** for 24 h significantly ameliorated OGD/R-induced mitochondrial dysfunction, confirming its protective role in myocardial cells through mitochondrial regulation.

We subsequently tested **SKLB-11A** in a mouse myocardial I/R injury model, administering the compound (20 mg/kg/day, ig) for 1 week. Echocardiography showed that **SKLB-11A** treatment restored cardiac function impaired by I/R injury, as indicated by improvements in EF and FS (Figure 6A). Additionally, **SKLB-11A** partially reversed I/R-induced elevations in serum myocardial injury markers, including LDH, CK-MB, and BNP (Figure 6B-D). Infarct size analysis further demonstrated a significant reduction in infarcted areas in **SKLB-11A**-treated mice compared to the I/R group (Figure 6E). Masson’s trichrome staining revealed that **SKLB-11A** markedly reduced I/R-induced cardiac fibrosis (Figure 6F). Collectively, these findings demonstrate that the potential efficacy of **SKLB-11A** in protecting against myocardial ischemia/reperfusion injury in mice.

**Figure 6:**
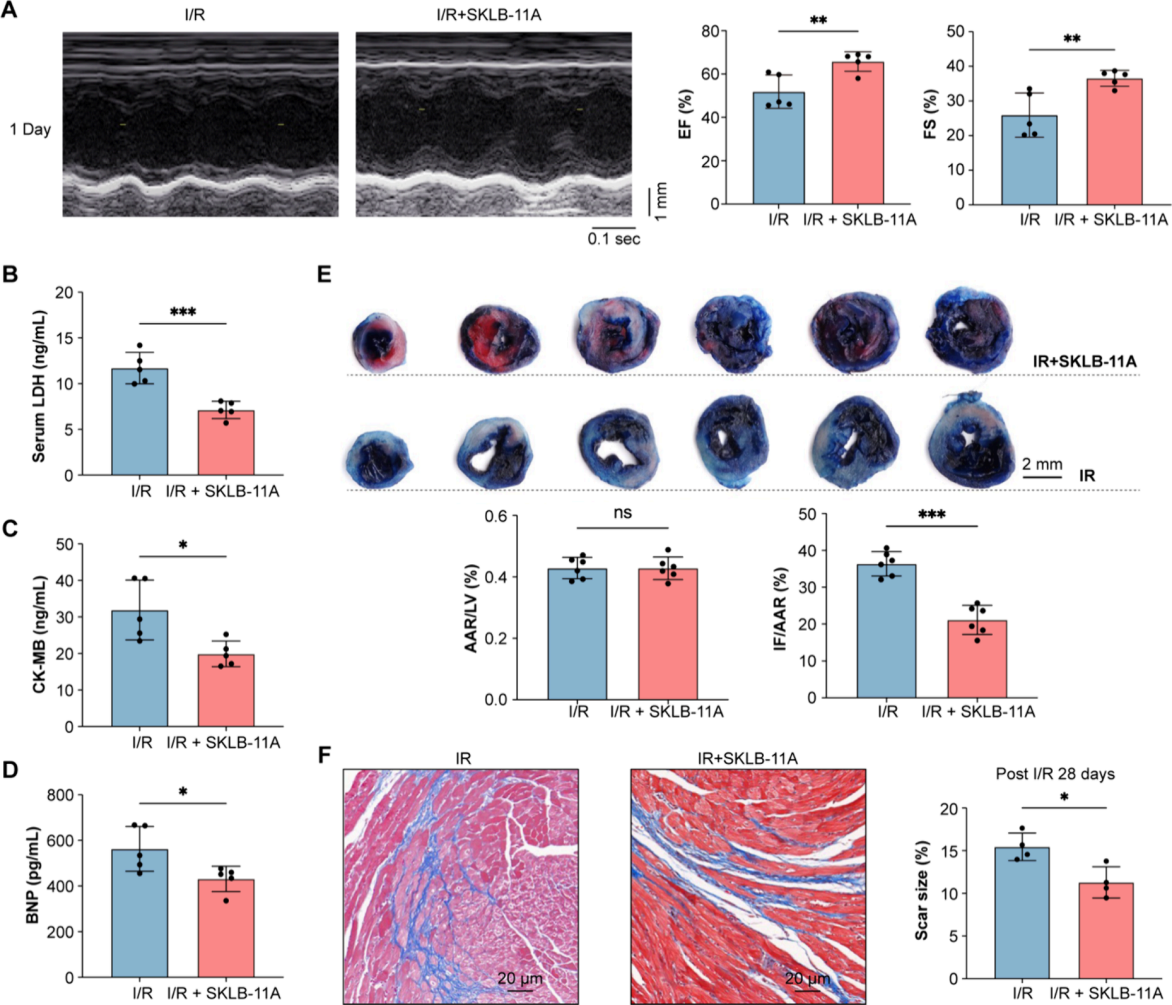
**SKLB-11A** ameliorates myocardial ischemia/reperfusion (I/R) injury *in vivo*. (A) Representative echocardiographic images from mice 24 h after I/R surgery, treated with or without **SKLB-11A** (20 mg kg^–1^ i.g.). (B–D) Serum LDH, CK-MB and BNP levels were measured in mice 24 h after I/R surgery, treated with or without **SKLB-11A** (20 mg kg^–1^ i.g.), *n* = 5 per group. (E) Evans blue/triphenyltetrazolium chloride (TTC) staining of heart sections in mice 24 h after I/R surgery, treated with or without **SKLB-11A** (20 mg kg^–1^ i.g.), scale bars:2 mm. (F) Representative images of Masson’s trichrome staining in mice 28 d after I/R surgery, treated with or without **SKLB-11A** (20 mg kg^–1^ i.g.), scale bars = 20 μm, *n* = 4 per group. Data are presented as mean ± SD, *p < 0.05, **p < 0.01, and ***p < 0.001, vs I/R group.

### Pharmacokinetic Properties of SKLB-11A

Given the potential of **SKLB-11A** for treating myocardial injury, we further evaluated its pharmacokinetic profile in Sprague–Dawley (SD) rats (Supplementary Table S5). The pharmacokinetics were assessed following both intravenous injection (1 mg/kg) and intragastric administration (10 mg/kg) of **SKLB-11A**. Following intravenous injection at a dose of 1 mg/kg, the area under the curve (AUC_0–∞_), peak concentration (*C*_max_), and half-life (*T*_1/2z_) of **SKLB-11A** were determined to be 185.40 ± 19.96 μg·h/L, 254.96 ± 3.42 μg/L, and 0.60 ± 0.06 h, respectively. The results indicate that **SKLB-11A** possessed acceptable pharmacokinetic properties.

## Discussion

SIRT3, which is widely expressed in mitochondria-rich tissues, plays a critical role in maintaining mitochondrial function and homeostasis, and is involved in the pathogenesis of various diseases.^55^ SIRT3 regulates mitochondrial function by deacetylating,^56^ and thereby activating, numerous mitochondrial proteins involved in energy metabolism,^57^ oxidative stress responses, and cellular respiration.^58^ To date, most SIRT3 modulators identified are nonspecific natural compounds or repurposed drugs, limiting their potential for clinical use.^59^ Despite extensive efforts, there remains a lack of SIRT3 activators with distinct binding modes for cardioprotection, making the development of targeted activators a critical research challenge.

To the best of our knowledge, most SIRT3 activators have been investigated using molecular docking to explore their binding modes with the SIRT3. For example, compound 33c interacted with key amino acids of SIRT3, including Glu177, Glu323, Phe294, Phe157, Arg158, and Phe180. The tetrahydropyrrole group inserted into the hydrophobic allosteric pocket formed by Phe157, Ile179, Pro176, Phe180, and Phe294, thereby enhancing the allosteric effect required for SIRT3 activation.^31^ Predictions suggested that SZC-6 interacts with the allosteric pocket surrounding Phe180 and Phe294 on the SIRT3 surface.^20^ Compound 4q adopts an induced-fit mechanism, fitting into a spacious cavity distinct from the NAD^+^ binding pocket, and engages in hydrogen bonding and hydrophobic interactions with R235, S253, D290, and Q300.^21^ In our ongoing efforts, we have determined the cocrystal structure of a small-molecule SIRT3 activator in complex with SIRT3, revealing that **SKLB-11A** forms a hydrogen bond with Val292. The pyrrole ring of **SKLB-11A** deeply inserts into a pocket formed by Phe180 and His248, and for the first time, we observe a conformational flip of the key amino acid Leu298, which may suggest a novel activation mechanism.

Autophagy, and particularly mitophagy, plays a central role in cardiovascular diseases, often triggered by stress conditions such as hypoxia and ischemia.^60^ Autophagy maintains a bidirectional regulatory relationship with cellular homeostasis: moderate levels of autophagy promote cell survival, whereas excessive autophagy leads to cell death.^61^ Similarly, SIRT3 exerts dual regulatory effects on autophagy, both promoting and inhibiting this process.^62,63^ Studies have revealed that melatonin inhibits autophagy while modulating the SIRT3-SOD2 signaling pathway, which helps protect against oxidative stress.^64^ SIRT3 promotes lipophagy and chaperon-mediated autophagy to protect hepatocytes against lipotoxicity.^65^ SIRT3 regulates mitophagy through multiple mechanisms. Among them, the PINK1/Parkin pathway is the most extensively studied ubiquitin-dependent autophagy pathway, with SIRT3 acting as an upstream regulator of this pathway.^66^ The protective role of autophagy has been validated across various disease models. For instance, studies have shown that SHISA9 protects mice from viral encephalitis-induced inflammatory damage via autophagy.^67^ Activation of autophagy has also been shown to reverse cardiac remodeling following myocardial infarction, significantly alleviating cardiac dysfunction and providing myocardial protection. Furthermore, promoting mitophagy reduces the accumulation of damaged organelles, substantially improving mitochondrial quality control.^68^ Based on these findings, we investigated whether **SKLB-11A** targets SIRT3 to regulate autophagy and its potential therapeutic effects. Remarkably, **SKLB-11A** was found to induce protective autophagy both *in vitro* and *in vivo* models. This is the first time that a SIRT3 activator with a distinct binding mode has been identified, and that it can induce protective autophagy to confer myocardial protection.

Taken together, we reported the structure-based discovery of **SKLB-11A**, a novel and selective SIRT3 activator with significant cardioprotective efficacy *in vitro* and *in vivo*. Additionally, **SKLB-11A** did not exhibit significant toxicity, highlighting its potential clinical value. **SKLB-11A** binds directly to SIRT3, activating its deacetylation function with higher selectivity compared to other sirtuin family members. Given the limited understanding of the precise interaction patterns between current SIRT3 activators and the protein, which have mostly been studied through molecular docking, our structural analysis reveals a novel allosteric activation mechanism involving amino acid flipping. Unlike previously reported activation modes, this mechanism provides key insights into SIRT3 activation by revealing a distinct binding site. In summary, **SKLB-11A** represents a promising therapeutic candidate for alleviating myocardial injury by modulating autophagy and mitochondrial function, offering potential for SIRT3-targeted activation in cardiac protection therapies.

## Materials and Methods

### Determination of Cell Viability

H9c2 cardiomyocytes were cultured in high-glucose DMEM supplemented with 10% FBS and 1% penicillin/streptomycin under a 5% CO_2_ atmosphere at 37 °C. Cells were seeded at 5 × 10^4^ cells/mL in 96-well plates and treated with 1 μM Dox for 24 h. After Dox treatment, cell viability was quantified via MTT assay; 3-(4,5-dimethythiazol-2-yl)-2,5-diphenyltetrazolium bromide (MTT, 5 mg/mL) was applied for 4 h, followed by DMSO dissolution of formazan crystals. Additionally, cells were pretreated with 10 μM **SKLB-11A** for 24 h prior to 1 μM Dox exposure, and viability was similarly assessed using MTT.

### Western Blotting

Proteomic analysis was conducted on H9c2 cells and murine cardiac tissues. Protein lysates were resolved via SDS-PAGE and transferred onto PVDF membranes. Blocking was achieved with 5% nonfat milk at ambient temperature for 1 h, followed by overnight incubation at 4 °C with specific primary antibodies. After washing, membranes were probed with secondary antibodies for 1 h at room temperature. Detection was facilitated by an ECL kit, with densitometry performed using ImageJ software to quantify protein expression, normalized against controls. The antibodies employed included: anti-SIRT3 (2627, Cell Signaling Technology), anti-acetylated lysine (9441, CST), anti-Ac.K68-MnSOD2 (ab137037, Abcam), anti-Ac.K122-MnSOD2 (ab214675, Abcam), anti-MnSOD2 (13141, CST), anti-LC3B (3868, CST), anti-p62 (8025, CST), anti-PINK1 (6946, CST), anti-Parkin (4211, CST), and anti-β-actin (ab8226, Abcam).

### Crystallization and Crystal Soaking

SIRT3 protein at a concentration of 10 mg/mL was incubated with **SKLB-11A** (200 mM) in a mole ratio of 1:5 on ice for 1–3 h. The mixture was then centrifuged at 4 °C for 10 min, and the supernatant was used for crystallization. The precipitant solution consisted of 0.2 M Li_2_SO_4_, 1.0 M (NH_4_)_2_SO_4_, and 0.1 M Tris, at pH 7.5. Crystals emerged after approximately 3 days at 16 °C on a sitting-drop plate. The crystals were subsequently transferred to a cryoprotectant solution containing 20% glycerol and were flash-cooled in a nitrogen gas stream at 100 K for synchrotron radiation X-ray diffraction data collection. **Site-directed mutagenesis.** L298G Primer forward: 5′-TCTTCGGT­GAACCGggc­CCGCAGCG­TTTCCTGCTG-3′; reverse: 5′-gccCGGTT­CACCGAAG­AAAACGA-3′; Site-directed mutagenesis was conducted using 2 x Phanta Flash Master Mix (Vazyme, P510). The mutated plasmid was sequenced by Sangon Biotech (Shanghai, China).

### X-ray Data Collection and Structure Determination

The SIRT3-**SKLB-11A**-ligand complex crystals were carefully positioned within nylon loops and subsequently vitrified in a gaseous nitrogen flow at 100 K. The collection of diffraction data ensued at Shanghai Synchrotron Radiation Facility’s (SSRF) beamlines BL17U1 and BL19U1, located in Shanghai. Data processing, encompassing indexing, integration, and scaling, was conducted via the HKL2000 software suite.^69^ The initial phases were ascertained by employing molecular replacement techniques, utilizing the extant SIRT3 structure as a search model. Manual model construction was undertaken using the program Coot, followed by iterative refinement with the phenix.refine module within the Phenix software framework.^70,71^ The final model’s structural integrity was rigorously evaluated and validated using the Protein Data Bank’s validation service.

### Virtual Screening

The structure of SIRT3 (PDB: 8V2N) was prepared using Schrödinger’s Protein Preparation module.^72^ The SiteMap module was utilized to predict the potential binding sites on SIRT3.^73^ Subsequently, the Glide Grid module generated a docking grid file for further docking investigations. The screening database was obtained from ChemDiv (https://www.chemdiv.com/) and underwent preprocessing with Schrödinger’s LigPrep module. This preprocessing included protonation, desalting, hydrogenation, tautomer and stereoisomer generation, and energy minimization. An initial ADMET filter was applied using the QuickProp module in Schrödinger, ensuring that only compounds adhering to Lipinski’s five rules and lacking reactive fragments progressed to the docking screening phase. High-throughput virtual screening (HTVS) was subsequently conducted using the Glide module in HTVS mode, retaining the top 10% of molecules for standard precision (SP) screening. From the SP screen, the top 10% of compounds were selected for a final high precision (XP) assessment. The leading 10% of molecules from the XP screen were then analyzed using MMGBSA to calculate binding free energy, with those exhibiting the highest MMGBSA scores chosen for further evaluation.^74^

### In Vitro SIRT3 Deacetylase Activity Assay

The SIRT3 activity was assessed using the Enzo Life Sciences kit (BML-AK557-0001) per the protocol provided.^64^ A 10 μM fluoro-acetylated p53 peptide and 500 μM NAD^+^ were added to SIRT3 assay buffer, incubated with test compounds and 0.5 μg recombinant SIRT3 at 37 °C for 30 min. In the mass spectrometry (MS) assay, 5 μM hSirt3 was reacted with 0.25 mM peptide and 1.25 mM NAD^+^, with or without 10 μM **SKLB-11A** in 2% DMSO, at 37 °C. Controls included 2% DMSO without **SKLB-11A**. The reaction was halted at defined intervals using 5 mM nicotinamide, and the mixture then underwent filtration and subjected to nano LC-MS/MS analysis. Acetylated Peptide: MnSOD-K122 (GELLEAIK-Ac).

### Surface Plasmon Resonance (SPR) Assay

The affinity of **SKLB-11A** for SIRT1, SIRT2, SIRT3, SIRT5, and SIRT6 was determined using a Reichert 4SPR system (Reichert Technologies, Depew, NY, USA). Proteins at 200 μg/mL were immobilized on a dextran-coated sensor chip (13206066) until reaching ≥12,000 response units (RUs). A dual-flow channel setup was used, reserving one channel for blank controls. **SKLB-11A** was introduced at varying concentrations for binding and subsequent dissociation kinetic analysis. Data acquisition was at 25 °C utilizing a PBST running buffer (pH 7.4). The equilibrium dissociation constant (*K*_d_) was computed from steady-state affinity measurements using TraceDrawer software.

### Biolayer Interferometry (BLI)

Binding kinetics of SIRT3 with various compounds were quantified using SSA biosensors on an Octet Red96 system (ForteBio).^75^ The assays occurred in 96-well plates, recording wavelength shifts in real-time. Ambient temperature was maintained at 25 °C, with plate agitation at 1000 rpm, and a reaction volume of 200 μL per well. SIRT3 protein and test compounds were prepared in phosphate-buffered saline (PBS, pH 7.4). Biosensors were equilibrated in PBS for 10 min before immobilizing SIRT3 (0.9 μg/mL) for 180 s, followed by a 120-s PBS wash. Binding interactions were monitored in wells with varying compound concentrations, and subsequent dissociation was observed in kinetic buffer. Data analysis was performed using ForteBio’s Octet RED software.

### Measurement of Intracellular Mitochondrial Membrane Potential and ROS

Mitochondrial membrane potential and reactive oxygen species (ROS) levels within cells were assessed using the JC-1 Mitochondrial Membrane Potential Assay Kit (Beyotime, Beijing, China) and the DCFH-DA probe (Beyotime, Shanghai, China), respectively, following the manufacturer’s guidelines.^76,77^ For mitochondrial membrane potential quantification, cells were treated with JC-1 solution and incubated for 20 min at 37 °C in the dark. ROS quantification involved a 25 min incubation at 37 °C in the dark with DCFH-DA solution. Postincubation, cells underwent three gentle washes with prewarmed buffer. Visualization was achieved using a Nikon (Ni-E) fluorescence microscope and quantified by flow cytometry (BD FACS Calibur).

### Quantification of ATP Content

ATP concentrations were detected by a firefly luciferin/luciferase-based ATP bioluminescence assay kit (Beyotime, China). The ATP contents were determined as nmol/mg protein by using SYNERGY 4 Microplate Reader (BioTek Instruments).

### The Cellular Thermal Shift Assay (CETSA)

The Cellular Thermal Shift Assay (CETSA) leverages the principle of thermal stabilization of proteins upon ligand binding. H9c2 cells were seeded on 100 mm culture dishes and allowed to adhere for 12 h. Following attachment, cells were treated with **SKLB-11A** (10 μM) for 6 h, after which they were enzymatically detached using trypsin and harvested. The cell suspension was partitioned into seven equal aliquots, which were then exposed to a range of thermal conditions (37, 41, 45, 49, 53, 57, and 61 °C) for 3 min each. Post-thermal treatment, rapid freezing in liquid nitrogen was performed thrice to ensure cell lysis. The lysates were then subjected to centrifugation at 12,000 rpm for 20 min, and the levels of SIRT3 were analyzed via Western blotting.

### OGD/R Injury Model in Vitro

An *in vitro* oxygen-glucose deprivation (OGD) injury model was established as described in a previous study. OGD injury was induced by incubating cells in glucose-free DMEM under hypoxic conditions (94% N_2_, 1% O_2_, and 5% CO_2_) for 6 h. Before the hypoxic phase, cells were treated with **SKLB-11A** (10 μM). The cells were then incubated in normoxic conditions with normal medium at 37 °C for the indicated time of 24 h as reperfusion. Untreated cells were used as controls.

### Mouse Model of Doxorubicin-Induced Cardiomyopathy and Drug Administration

For the establishment of the experimental model, male C57BL/6J mice aged 8 weeks, with an average weight of 23 g, were utilized. The mice were administered varying dosages of **SKLB-11A** (10 and 20 mg/kg/day) or a corresponding volume of sterile saline via oral gavage over a period of 7 days. This was followed by intraperitoneal injections of doxorubicin (Dox), distributed into five equal doses totaling 15 mg/kg across 2 weeks, or a comparable volume of sterile saline. The sample size for each group consisted of eight specimens. Echocardiographic assessments were conducted at three distinct time points: baseline (day 0), post-treatment (day 7), and at the conclusion of the study (day 21). The experimental timeline was terminated on day 21.

### Mouse Model of Myocardial I/R Injury and Drug Administration

All animal experiments were conducted in compliance with the guidelines established by the Animal Care and Use Committee of West China Hospital. Healthy male C57BL/6J mice (8–12 weeks old) were randomly assigned to two groups: (1) I/R group (100 μL saline administered daily for 7 days), and (2) I/R + **SKLB-11A** group (20 mg/kg **SKLB-11A** administered daily for 7 days). Myocardial ischemia-reperfusion (I/R) injury was induced via left intercostal thoracotomy and left anterior descending coronary artery (LAD) ligation. Briefly, mice were anesthetized with 3% isoflurane, orally intubated, and mechanically ventilated using a Harvard Apparatus Rodent Ventilator (Model 845) at a rate of 100–110 breaths/min and a tidal volume of 150–200 μL. During surgery, a mix of oxygen and 1.5–2% isoflurane was administered, and body temperature was maintained at 35–36 °C using a heating pad and monitored with a rectal probe thermometer. A thoracotomy was performed through the third and fourth intercostal spaces, and myocardial ischemia was induced by placing a 7–0 silk suture around the LAD with a PE-50 tubing. Ischemia was confirmed by regional cyanosis. After 30 min of LAD occlusion, reperfusion was initiated by removing the PE-10 tubing, verified by a hyperemic response. The thoracotomy site was closed with rib reapproximation, and reperfusion was maintained for 24 h or 28 days. Subsequent analyses were performed on myocardial I/R-injured mice.

### Echocardiography

Noninvasive transthoracic echocardiography was performed using a Vevo 3100 System with a 30-MHz transducer to assess cardiac function in mice. The procedure was conducted by an experienced technician who was blinded to the study groups. Mice were anesthetized with 1.5–2% isoflurane and underwent skin preparation. Two-dimensional guided M-mode echocardiography in the short axis view was used to measure left ventricular fractional shortening (FS) and left ventricular ejection fraction (EF).

### H&E Staining

H&E staining was used to evaluate pathological changes in key organs. Fixed tissues were dehydrated, embedded in paraffin, and sectioned into 4 μm slices. The sections were stained with hematoxylin for 10–15 min, washed with tap water for 2 min, differentiated for 30 s using hydrochloric acid and ethanol, and then washed with warm water for 5 min. After staining with eosin for 2 min, the sections were dehydrated and mounted with resin. All procedures followed the standard operating procedure (SOP) for pathological examination.

### Assessment of Myocardial Infarct Size

After assessing cardiac function, blood was collected, and the heart was quickly sectioned for 1% Evans blue dye (Sigma, Darmstadt, Germany).The heart was subsequently frozen at −20 °C for 30 min. The heart was then sectioned into 2 mm thick transverse slices and incubated in 1% 2,3,5-triphenyl tetrazolium chloride (TTC, Sigma-Aldrich, St. Louis, MO, USA) at 37 °C for 15 min. Digital images of the heart slices were captured and analyzed for infarct size using planimetry in ImageJ. Infarct size (%) was calculated as the ratio of myocardial infarct area to left ventricular area.

### Statistical Analysis

Data were subjected to statistical evaluation via GraphPad Prism software. For experimental outcomes encompassing multiple groups, one-way analysis of variance (ANOVA) was employed, whereas comparisons involving two groups were analyzed using two-tailed *t* tests. Experimental procedures were replicated three times, with a minimum of three parallel measurements for each condition. Results are presented as mean ± standard deviation (SD) or standard error of the mean (SEM), with p-values less than 0.05 denoting statistical significance.

## Supplementary Material



## Data Availability

All the data supporting the findings of this study are available from the corresponding author on reasonable request.

## Notes

Supporting figures, supporting tables, biochemical methods, chemistry and compounds characterization, as well as copies of ^1^H NMR, ^13^C NMR, HRMS and HPLC spectra, and uncropped blots (DOCX)
