# Supplementary material for: Unraveling Small Molecule-Mediated Sirtuin 3 Activation at a Distinct Binding Site for Cardioprotective Therapies
Source: ACS Cent Sci. 2025 Apr 14;11(5):704–18. doi: 10.1021/acscentsci.5c00023 (PMC12123458; doi:10.1021/acscentsci.5c00023)
Supplement: Supplementary file 1 [file oc5c00023_si_001.docx]

**Supporting information**

**Unraveling Small Molecule-Mediated Sirtuin 3 Activation at a Distinct Binding Site for Cardioprotective Therapies**

Dan Zhang^1,#^, Jifa Zhang^1,#^, Chengyong Wu^1,#^, Yao Xiao^1^, Liwei Ji^2^, Jiarui Hu^1^, Jianjun Ding^4^, Tao Li^2^*, Yiwen Zhang^1^*, Liang Ouyang^1,3^*

^1^ Department of Biotherapy, State Key Laboratory of Biotherapy and Cancer Center, Laboratory of Neuro-system and Multimorbidity, West China Hospital, Sichuan University, Chengdu, 610041, Sichuan, China.

^2^ Department of Anesthesiology, National Clinical Research Center for Geriatrics, West China Hospital, Sichuan University, Chengdu, 610041, Sichuan, China.

^3^ Frontiers Medical Center, Tianfu Jincheng Laboratory, Chengdu, 610212, Sichuan, China.

^4^ School of Food Science and Technology, Jiangnan University, Wuxi, 214122, Jiangsu, China.

^#^ These authors contributed equally to this work.

* Corresponding author.

E-mail address: scutaoli1981@scu.edu.cn(T. Li); yiwenzhang@scu.edu.cn(YW. Zhang); ouyangliang@scu.edu.cn(L. Ouyang).

**Table of Content**

**[1.](#_Toc193561658)****[Supporting figures](#_Toc193561658)** [S3](#_Toc193561658)

**[2.](#_Toc193561659)****[Supporting tables](#_Toc193561659)** [S9](#_Toc193561659)

**[3.](#_Toc193561660)****[Synthetic procedures](#_Toc193561660)** [S15](#_Toc193561660)

**[4.](#_Toc193561661)****[NMR, HRMS and HPLC spectra](#_Toc193561661)** [S31](#_Toc193561661)

**[5.](#_Toc193561662)****[Uncropped blots](#_Toc193561662)** [S82](#_Toc193561662)

1. **Supporting figures**


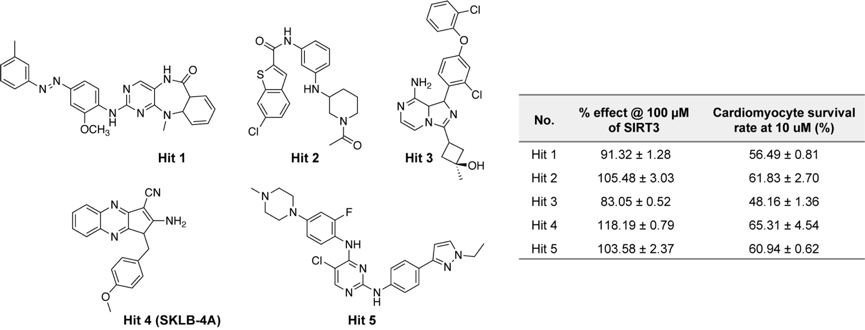


**Supplementary Figure S1.** The top 5 candidate compounds of SIRT3 were selected.


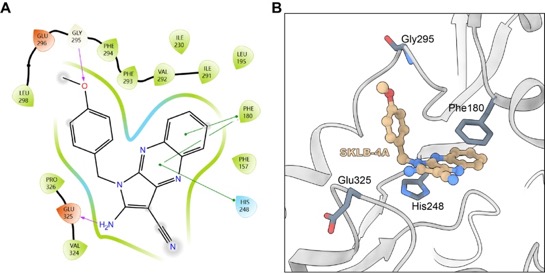


**Supplementary Figure S2.** (A) 2D interaction diagram of SIRT3 and **SKLB-4A**. (B) Molecular docking of **SKLB-4A** and SIRT3. The key residues involved in the interaction are labeled and shown as sticks.


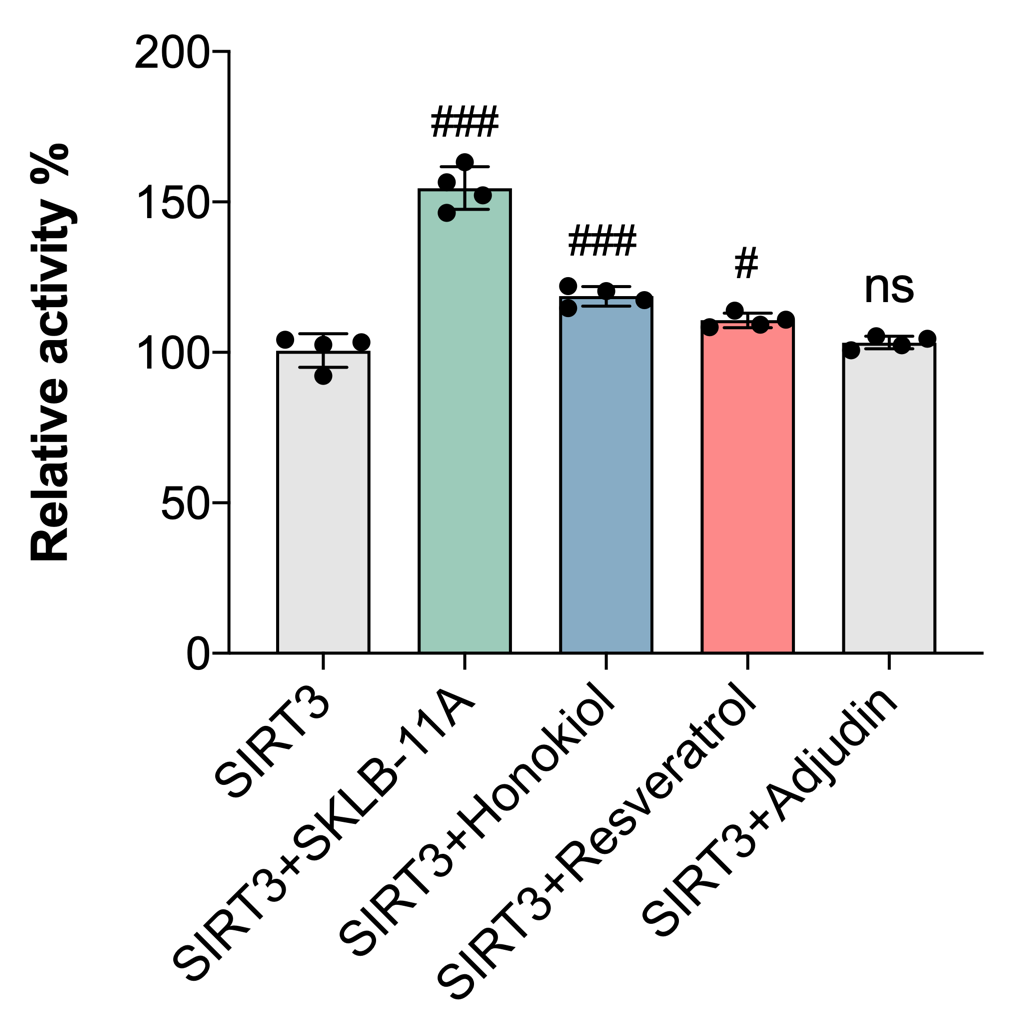


**Supplementary Figure S3.** Effects of **SKLB-11A**, honokiol, resveratrol, and adjudin on SIRT3-dependent peptide deacetylation by coupled enzymatic assays. ns, no significance; ^#^p < 0.05, ^##^p < 0.01, ^###^p < 0.001 vs control group.


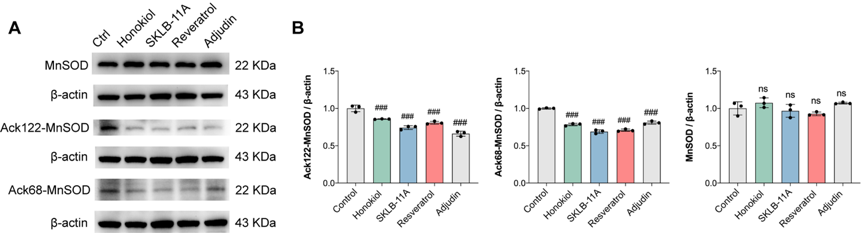


**Supplementary Figure S4.** Western blot analysis of MnSOD, SIRT3, and acetylated MnSOD (at K68 and K122) in H9c2 cells treated with honokiol, **SKLB-11A**, resveratrol, and adjudin for 24 h. β-actin, loading control. ns, no significance; ^#^p < 0.05, ^##^p < 0.01, ^###^p < 0.001 vs control group.

**
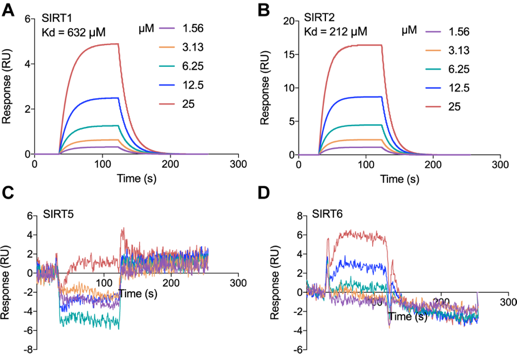
**

**Supplementary Figure S5.** Surface Plasmon Resonance (SPR) analysis of the binding of **SKLB-11A** with SIRT1, SIRT2, SIRT5, and SIRT6.


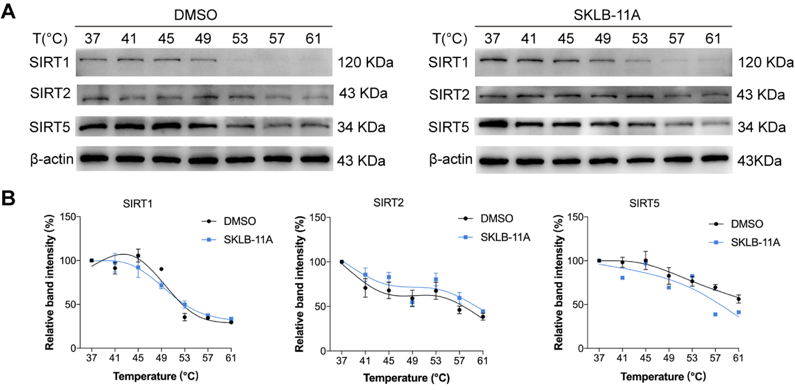


**Supplementary Figure S6.** CETSA assay detected the thermal stability of SIRT1, SIRT2, SIRT3, and SIRT5 in H9c2 cells treated with **SKLB-11A**, respectively.


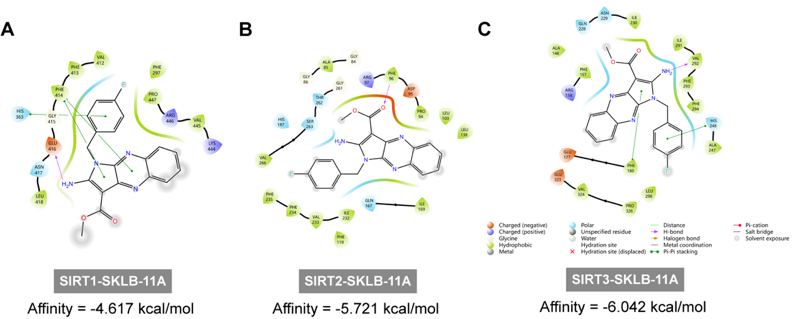


**Supplementary Figure S7.** Predicted two-dimensional interaction between **SKLB-11A** and SIRT1, SIRT2 and SIRT3. The PDB entries for SIRT1, SIRT2, SIRT3 are 4ZZJ, 5G4C, and 8V2N, respectively.


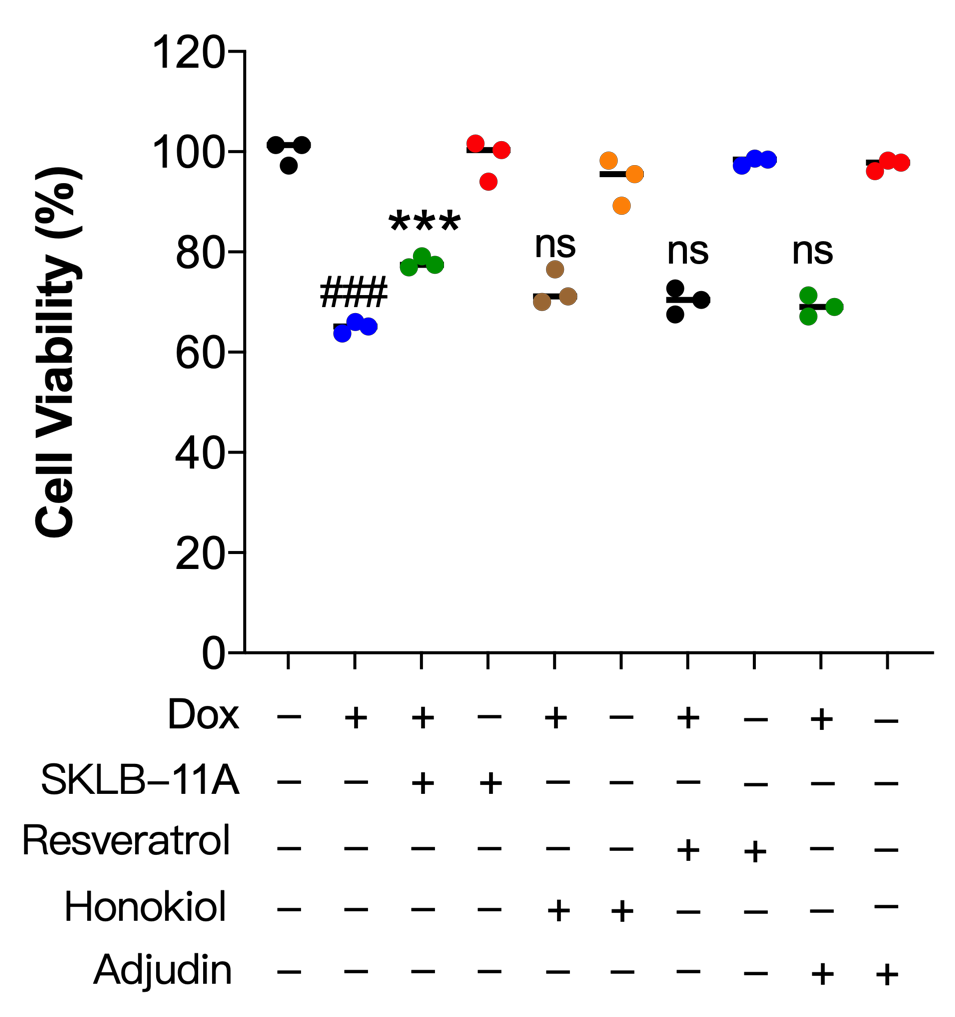


**Supplementary Figure S8.** H9c2 cells were treated with indicated concentration of compounds for 24 h, and then treated with Dox for 24 h, the cell viability was measured by the MTT assay. Data are present as mean ± SD, n = 3. *p<0.05, **p<0.01, and ***p<0.001, vs. Dox treatment group, ^#^p < 0.05, ^##^p < 0.01, ^###^p < 0.001 vs control group.


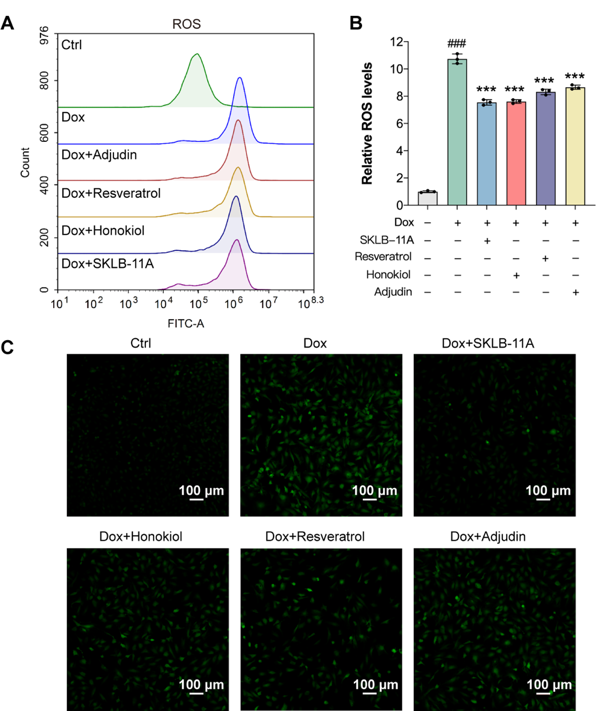


**Supplementary Figure S9.** H9c2 cells were treated with Dox in the presence or absence of 10 μM honokiol, **SKLB-11A**, resveratrol, and adjudin. Cells were stained with DCFH-DA. ROS levels were observed using flow cytometry and fluorescence microscopy. The data are presented as the mean ± SD. *p<0.05, **p<0.01, and ***p<0.001, vs. Dox treatment group, ^#^p < 0.05, ^##^p < 0.01, ^###^p < 0.001 vs control group.


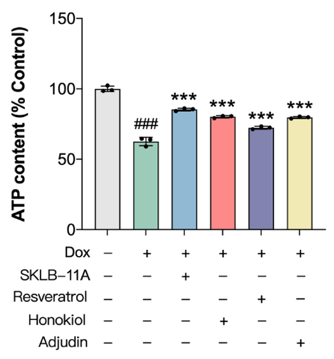


**Supplementary Figure S10.** H9c2 cells were treated with Dox in the presence or absence of 10 μM honokiol, **SKLB-11A**, resveratrol, and adjudin. The level of ATP was tested by ATP Assay Kit. The data are presented as the mean ± SD. *p<0.05, **p<0.01, and ***p<0.001, vs. Dox treatment group, ^#^p < 0.05, ^##^p < 0.01, ^###^p < 0.001 vs control group.

**
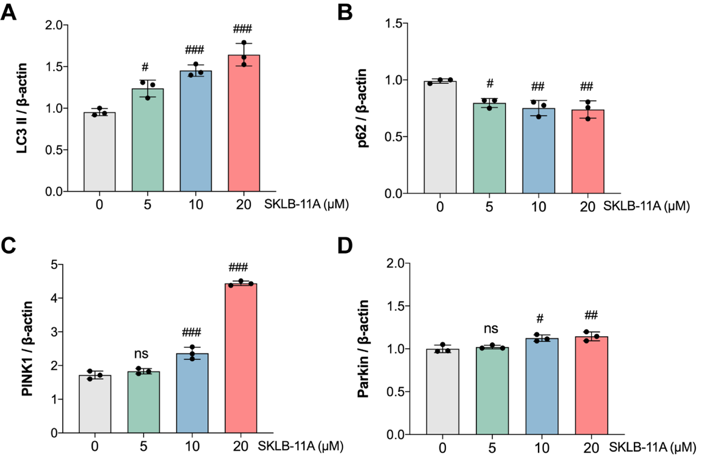
**

**Supplementary Figure S11.** Western blot analysis of LC3-II, p62, PINK1 and Parkin protein expression in H9c2 cells treated with **SKLB-11A** for 24 h. β-actin, loading control. ns, no significance; ^#^p < 0.05, ^##^p < 0.01, ^###^p < 0.001, vs control group.


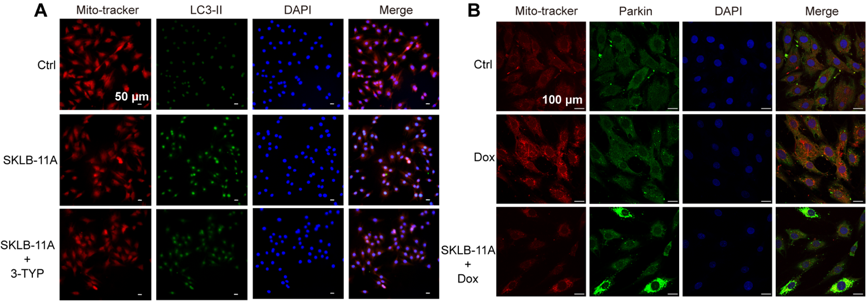


**Supplementary Figure S12.** (A) Differences in LC3-II expression among different treatment groups were detected via immunofluorescence. (B) Differences in Parkin fluorescent signal in mitochondria were assessed using confocal microscopy.


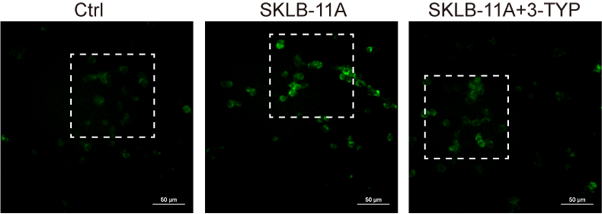


**Supplementary Figure S13.** After treating H9c2 cells with **SKLB-11A** (10 μM) for 24 h, the autophagic vacuoles were stained with MDC (green) and observed under fluorescence microscopy. Scale bar, 50 μm.


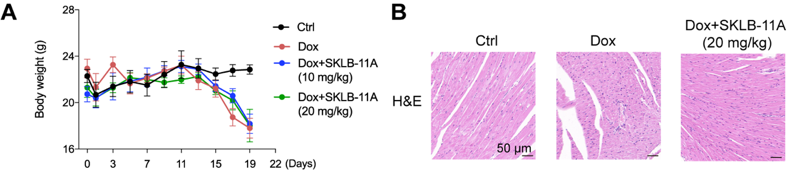


**Supplementary Figure S14.** (A) Changes in mouse body weight during administration. All data were expressed as mean ± SD, (n = 8). (B) Representative H&E-stained images of myocardial morphology and structural changes in different mice group.


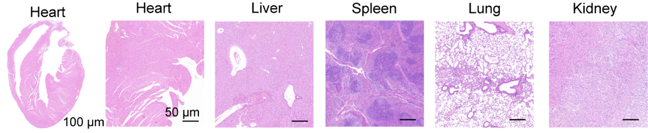


**Supplementary Figure S15.** H&E staining of various organs in mice treated exclusively with **SKLB-11A**.


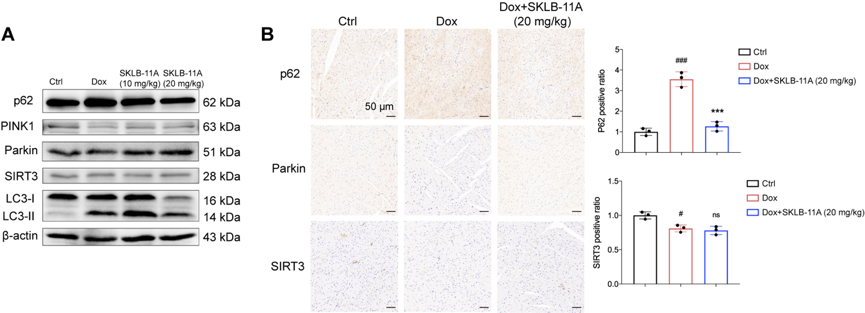


**Supplementary Figure S16.** (A) Expression levels of SQSTM1/p62, PINK1, Parkin, SIRT3 and LC3 were examined by western blotting, β-actin was used as a loading control. (B) Immunohistochemical analysis of p62, Parkin and SIRT3 in control, Dox, and **SKLB-11A** treated group.


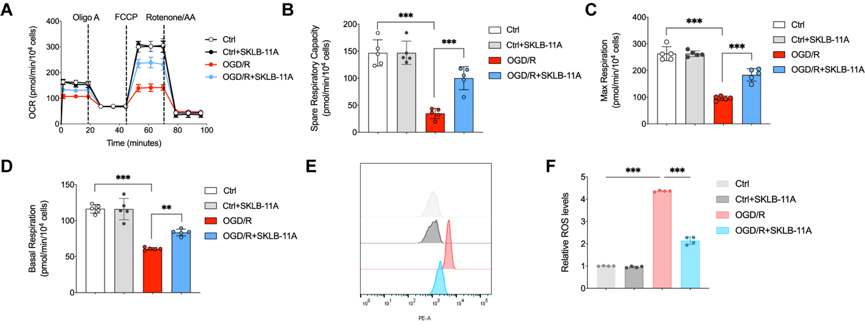


**Supplementary Figure S17.** (A-D) Mitochondrial respiration was measured including maximal respiration, spare respiratory capacity, and basal respiration by agilent seahorse analyzer under normoxia or HR conditions. n = 5 in each group. (E-F) ROS content of H9c2 cells under normoxia or HR condition. Data were expressed as mean ± SD; **p*<0.05, ***p*<0.01, and ****p*<0.001.

1. **Supporting tables**

**Supplementary Table S1.** The score and proportion of different prediction sites.

| **Title** | **Dscore** | **Sitescore** | **Volume** |
| --- | --- | --- | --- |
| S1 | **1.092** | 1.055 | 230.153 |
| S2 | **0.948** | 0.913 | 228.095 |
| S3 | **0.886** | 0.829 | 122.451 |
| S4 | **0.57** | 0.615 | 68.6 |

**Supplementary Table S2.** Biochemical and Biological Evaluations of Candidate Compounds **SKLB-4A-F**, **5A**, **7A**, **11A-Q**.

| **Entry** | **Compound** | **% effect @ 100 µM of**  **SIRT3 ^[a]^** | **Cardiomyocyte survival rate**  **at 10 uM (%)** |
| --- | --- | --- | --- |
| 1 |   **SKLB-4A** | 118.19 ± 0.79 | 65.31 ± 4.54 |
| 2 |   **SKLB-4B** | 130.57 ± 0.36 | 70.78 ± 3.03 |
| 3 |   **SKLB-4C** | 126.37 ± 0.81 | 68.25 ± 0.51 |
| 4 |   **SKLB-4D** | 103.12 ± 1.02 | 64.43 ± 3.28 |
| 5 |   **SKLB-4E** | 110.39 ± 0.95 | 65.89 ± 2.41 |
| 6 |   **SKLB-4F** | 105.92 ± 1.96 | 65.17 ± 0.38 |
| 7 |   **SKLB-5A** | 154.01 ± 2.69 | 75.47 ± 2.69 |
| 8 |   **SKLB-7A** | 109.30 ± 0.71 | 66.14 ± 0.80 |
| 9 |   **SKLB-11A** | 188.14 ± 1.58 | 78.88 ± 0.65 |
| 10 |   **SKLB-11B** | 167.39 ± 1.13 | 76.97 ± 1.96 |
| 11 |   **SKLB-11C** | 124.20 ± 0.84 | 70.23 ± 1.89 |
| 12 |   **SKLB-11D** | 147.65 ± 3.07 | 74.35 ± 1.70 |
| 13 |   **SKLB-11E** | 130.64 ± 1.27 | 71.26 ± 4.34 |
| 14 |   **SKLB-11F** | 122.73 ± 0.59 | 69.00 ± 1.22 |
| 15 |   **SKLB-11G** | 107.19 ± 1.42 | 63.93 ± 0.62 |
| 16 |   **SKLB-11H** | 125.67 ± 0.91 | 68.70 ± 4.61 |
| 17 |   **SKLB-11I** | 134.80 ± 2.43 | 70.83 ± 3.76 |
| 18 |   **SKLB-11J** | 150.16 ± 1.75 | 72.42 ± 2.21 |
| 19 |   **SKLB-11K** | 144.27 ± 0.82 | 71.46 ± 2.09 |
| 20 |   **SKLB-11L** | 123.62 ± 0.95 | 67.11 ± 1.60 |
| 21 |   **SKLB-11M** | 104.18 ± 1.75 | 64.45 ± 0.87 |
| 22 |   **SKLB-11N** | 119.07 ± 3.30 | 66.26 ± 4.52 |
| 23 |   **SKLB-11O** | 126.46 ± 1.32 | 69.15 ± 1.35 |
| 24 |   **SKLB-11P** | 116.21 ± 0.57 | 65.75 ± 4.18 |
| 25 |   **SKLB-11Q** | 118.02 ± 0.63 | 66.48 ± 1.19 |
| 26 | **Model (Dox)** | / | 61.93 ± 0.51 |

^[a]^ The experiments were repeated three times, and the results are expressed as the mean ± SD.

**Supplementary Table S3.** ﻿Sirtuin substrate peptides

| Sirtuin | Peptide name | Peptide sequence | Protein of origin |
| --- | --- | --- | --- |
| SIRT1 | p53-acK381 | RHK-acK-LMFK | p53 |
| SIRT2 | αTub-acK25 | MPSD-acK-TIG | α-tubulin |
| SIRT3 | MnSOD-acK122 | GELLEAIK-ac | MnSOD2 |
| SIRT5 | CPS1-succK537 | RGVL-succK-EYGV | carbamoylphosphate synthetase 1 |

**Supplementary Table S4.** ﻿Data collection and refinement statistics.

| **SIRT3-SKLB-11A**  **(PDB code: 9KTK)** | |
| --- | --- |
| **Data collection** | |
| Space Group | P212121 |
| Unit Cell (Å) | 71.58 120.36 134.97 |
| Unit Cell (°) | 90.0, 90, 90 |
| Wavelength (Å) | 0.97853 |
| Resolution (Å) | 67.49-2.49 |
| R_merge_ | 0.085 (0.838) |
| CC_1/2_ | 99.9 (82.6) |
| I/sigma | 18.7 (2.6) |
| Completeness (%) | 99.9 (99.7) |
| Redundancy | 12.6 (10.4) |
| **Structure refinement** | |
| Number of measured reflections | 524760 |
| Number of unique reflections | 41592 |
| R_work_ / R_free_ (%) | 26.17/ 34.02 |
| No. atoms | 8634 |
| Protein | 8523 |
| Others | 111 |
| Average B value (Å^2^) | 64.71 |
| Protein | 64.86 |
| Others | 53.68 |
| **R.m.s. deviations** | |
| Bonds (Å) | 0.011 |
| Angle (°) | 1.324 |
| **Ramachandran plot statistics (%)** | |
| Most favorable | 90.08 |
| allowed | 7.32 |
| Disallowed | 2.59 |

**Supplementary Table S5.** Pharmacokinetic properties of **SKLB-11A** in SD

| **parameter** | **SD rats^a^** | |
| --- | --- | --- |
|  | **i.v. (1 mg/kg)** | **p.o. (10 mg/kg)** |
| *T*_1/2z_ (h) | 0.60 ± 0.06 | 5.44 ± 2.60 |
| *T_max_* (h) | 0.08 ± 0.00 | 0.25 ± 0.00 |
| *C_max_* (µg/L) | 254.96 ± 3.42 | 67.86 ± 13.02 |
| MRT_0–∞_ (h) | 0.72 ± 0.06 | 4.44 ± 1.78 |
| AUC_0–t_ (µg·h/L) | 185.22 ± 19.86 | 123.09 ± 23.65 |
| AUC_0–∞_ (µg·h/L) | 185.40 ± 19.96 | 127.60 ± 29.20 |
| V_Z_ (L/kg) | 4.66 ± 0.20 | 39.99 ± 12.09 |
| CL_Z_ (L/h·kg) | 5.44 ± 0.62 | 5.39 ± 1.16 |

^a^ SD rats were treated with 1 mg/kg or 10 mg/kg of **SKLB-11A**. Values are the average of three experiments. *T*_1/2_, half-life; *T*_max_, time to peak, *C*_max_, maximum concentration.

1. **Synthetic procedures**

**3.1** **Materials and measurements**

^1^H-NMR spectra were recorded at 400 MHz. The chemical shifts were recorded in ppm relative totetramethylsilane and with the solvent resonance as the internal standard. Data were reported as follows: chemical shift, multiplicity (s = singlet, d = doublet, t = triplet, dd = double of doublet, q = quartet, m = multiplet), coupling constants (Hz), integration. ^13^C-NMR data were collected at 100 MHz with complete proton decoupling. Chemical shifts were reported in ppm from the tetramethylsilane with the solvent resonance as internal standard. ESI-HRMS spectra were recorded on a commercial apparatus and methanol was used to dissolve the sample. All chemicals were obtained from commercial sources and used without further purification. Column chromatography was carried out on silica gel (300-400 mesh, Qingdao Marine Chemical Ltd, Qingdao, China). Thin layer chromatography (TLC) was performed on TLC silica gel 60 F254 plates. The purity of compounds was determined to be over 95% (> 95%) by reverse-phase high performance liquid chromatography (HPLC) analysis. HPLC instrument: SHIMADZU HPLC (Column: Diamonsil C18-WR, 5.0 μm, 4.6 x 250 mm (WondaSil); Detector: SPD-20A Photodiode Array; Injector: SIL-20A Autoinjector; Pump: LC-20AT). Elution: MeOH in water; Flow rate: 1.0 mL/min.

- 1. **Chemistry Methods.**

**
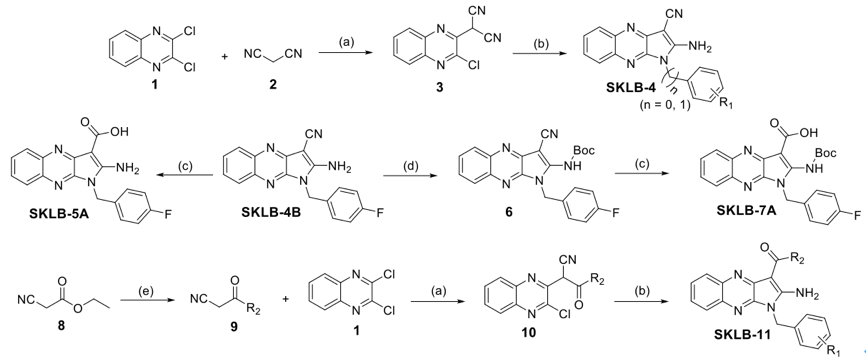
**

**Scheme S1.** Reagents and conditions: (a) NaH, DME, reflux; (b) ArNH_2_, NMP, 130℃; (c) KOH, 75% EtOH, reflux for 19h; (d) Boc_2_O, DCM, rt; (e) Amines or alcohols, Et_3_N, THF, rt.

**3.3 General synthesis procedure for Compounds SKLB-4A**-**SKLB-4F**

******

Malononitrile (**2**) (2.03 equiv.) was carefully added in portions to a 100 mL DME suspension containing sodium hydride (2.07 equiv.) and stirred at room temperature for 30 minutes. After the addition of compound **1** (1.0 equiv.), stirring was continued at room temperature for an additional 10 minutes, followed by heating to reflux for 1 hour. Subsequently, the mixture was evaporated under reduced pressure, and cold 1 M hydrochloric acid was added to obtain a yellow crude product. The product was filtered and washed with water and ethanol, yielding intermediate **3** as a yellow solid with a 62% yield.

To a solution of **3** (1.0 equiv.) in 1 : 1 EtOH – toluene solution (0.09 M) the primary amine (4.0 equiv.) was added. The reaction mixture was heated to 130-160 °C for 4-12 h and cooled down. The formed solid was filtered off and washed with Et_2_O and EtOH to obtain the desired series compounds **SKLB-4**. Compounds **SKLB-4A**-**SKLB-4F** were purified by flash column chromatography (petroleum ether/EtOAc, 1:1).

*2-amino-1-(4-methoxybenzyl)-1H-pyrrolo[2,3-b]quinoxaline-3-carbonitrile* ***(SKLB-4A)***

**SKLB-4A**

Light yellow solid, mp 271.7-278.0, yield 61%. ^1^H-NMR (400 MHz, DMSO*-d*_6_), *δ* (ppm): 8.64 (s, 2H), 7.94 (2H, m, *J* = 7 Hz), 7.88 (d, *J* = 7 Hz, 2H), 7.23 (d, *J* = 7 Hz, 2H), 6.87 (d, *J* = 7 Hz, 2H), 5.38 (s, 2H), 3.69 (s, 3H); ^13^C-NMR (100 MHz, DMSO*-d*_6_), *δ* (ppm): 160.0, 159.2, 144.0, 142.2, 140.8, 137.6, 128.9 (3), 128.3 (2), 128.1, 127.6, 127.3, 126.2, 115.7, 114.5, 60.8, 43.0. HRMS(ESI)^+^ Calculated for C_19_H_15_N_5_O, Exact Mass: 329.1277, [M+H]^+^: *m/z* 330.1349, found *m/z* 330.1347 . Purity: 97.46% (HPLC, *t*_R_ = 4.54 min).

*2-amino-1-(4-fluorobenzyl)-1H-pyrrolo[2,3-b]quinoxaline-3-carbonitrile* ***(SKLB-4B)***

**SKLB-4B**

Light yellow solid, mp 198.9-203.1, yield 56%. ^1^H-NMR (400 MHz, DMSO*-d*_6_), *δ* (ppm): 7.78 (s, 2H), 7.46 (m, 1H), 7.40 (s, 2H), 7.36 (m, 1H), 7.26 (m, 2H), 7.13 (m, 2H), 5.42 (s, 2H), 5.40 (s, 1H); ^13^C-NMR (100 MHz, DMSO*-d*_6_), *δ* (ppm): 160.7, 160.1, 144.1, 142.2, 140.9, 137.6, 132.6 (2), 129.6 (2), 129.5, 128.0, 127.7, 127.3, 126.2, 116.0, 60.9, 42.8. HRMS(ESI)^+^ Calculated for C_18_H_12_FN_5_, Exact Mass: 317.1077, [M+H]^+^: *m/z* 318.1150, found *m/z* 318.1149. Purity: 95.45% (HPLC, *t*_R_ = 6.04 min).

*2-amino-1-(4-bromobenzyl)-1H-pyrrolo[2,3-b]quinoxaline-3-carbonitrile* ***(SKLB-4C)***

**SKLB-4C**

Light yellow solid, mp 271.8-276.2, yield 30%. ^1^H-NMR (400 MHz, DMSO*-d*_6_), *δ* (ppm): 8.69 (s, 2H), 7.95 (d, *J* = 7.8 Hz, 1H), 7.88 (d, *J* = 7.8 Hz, 1H), 7.60 (m, 1H), 7.55 (m, 2H), 7.49 (m, 1H), 7.28 (m, 1H), 7.17 (m, 1H), 5.46 (s, 2H). ^13^C-NMR (100 MHz, DMSO*-d*_6_), *δ* (ppm): 160.1, 144.1, 142.2, 140.9, 139.1, 137.6, 131.8, 131.4, 130.9, 130.3, 128.0, 127.7, 127.3, 126.3, 122.2, 115.7, 61.0, 43.0. HRMS(ESI)^+^ Calculated for C_18_H_12_BrN_5_, Exact Mass: 377.0276, [M+H]^+^: *m/z* 378.0349, found *m/z* 378.0349. Purity: 99.80% (HPLC, *t*_R_ = 4.42 min).

*2-amino-1-(3-methoxyphenyl)-1H-pyrrolo[2,3-b]quinoxaline-3-carbonitrile* ***(SKLB-4D)***

**SKLB-4D**

Light yellow solid, mp 235.7-239.1, yield 51%. ^1^H-NMR (400 MHz, DMSO*-d*_6_), *δ* (ppm): *δ* 8.32 (s, 2H), 7.94 (d, *J* = 6.9 Hz, 1H), 7.78 (d, *J* = 6.7 Hz, 1H), 7.63-7.45 (m, 3H), 7.21-7.14 (m, 2H), 7.14 (d, *J* = 7.3 Hz, 1H), 3.83 (s, 3H). ^13^C-NMR (100 MHz, DMSO*-d*_6_), *δ* (ppm): 160.7, 160.2, 144.1, 143.2, 140.8, 137.7, 133.3, 131.1, 128.1, 127.5, 127.3, 126.1, 121.4, 115.9, 115.6, 115.0, 61.0, 55.9. HRMS(ESI)^+^ Calculated for C_18_H_13_N_5_O, Exact Mass: 315.1120, [M+H]^+^: *m/z* 316.1193, found *m/z* 316.1200. Purity: 95.10% (HPLC, *t*_R_ = 11.07 min).

*2-amino-1-(4-methoxyphenyl)-1H-pyrrolo[2,3-b]quinoxaline-3-carbonitrile* ***(******SKLB-4E)***

**SKLB-4E**

Light yellow solid, mp 234.7-237.3, yield 51%. ^1^H-NMR (400 MHz, DMSO*-d*_6_), *δ* (ppm): 8.25 (s, 2H), 7.93 (d, *J* = 6.8 Hz, 1H), 7.75 (d, *J* = 6.7 Hz, 1H), 7.62-7.53 (m, 1H), 7.52-7.43 (m, 3H), 7.18 (d, *J* = 8.9 Hz, 2H), 3.87 (s, 3H). ^13^C-NMR (100 MHz, DMSO*-d*_6_), *δ* (ppm): 160.6, 160.5, 144.2, 143.6, 140.8, 137.7, 130.8 (2), 128.0, 127.5, 127.2, 126.1, 124.7, 115.7 (2), 115.6, 60.8, 56.0. HRMS(ESI)^+^ Calculated for C_18_H_13_N_5_O, Exact Mass: 315.1120, [M+H]^+^: *m/z* 316.1193, found *m/z* 316.1196. Purity: 99.56% (HPLC, *t*_R_ = 5.13 min).

*2-amino-1-(2-fluorophenyl)-1H-pyrrolo[2,3-b]quinoxaline-3-carbonitrile* ***(SKLB-4F)***

**SKLB-4F**

Light yellow solid, mp 263.7-267.2, yield 46%. ^1^H-NMR (400 MHz, DMSO*-d*_6_), *δ* (ppm): 11.92 (s, 1H), 8.53 (s, 2H), 7.96 (m, 1H), 7.77 (m, 1H), 7.72 (m, 2H), 7.60 (m, 2H), 7.49 (m, 2H), 7.11 (m, 2H). ^13^C-NMR (100 MHz, DMSO*-d*_6_), *δ* (ppm): 157.7, 144.1, 142.9, 141.0, 137.6, 132.9, 128.1, 127.6, 126.4, 126.3, 126.2, 126.1, 123.5, 119.9, 117.7, 115.6, 61.3. HRMS(ESI)^+^ Calculated for C_17_H_10_FN_5_, Exact Mass: 303.0920, [M+H]^+^: *m/z* 304.0998, found *m/z* 304.0995. Purity: 98.17% (HPLC, *t*_R_ = 9.78 min).

**3.4 The synthesis of compound SKLB-5A**

******

To a solution of KOH in EtOH (75%) the **SKLB**-**4B** derivative was added. The reaction mixture was heated to 100 °C for 19 h, evaporate the mixture in a rotary evaporator under vacuum to obtain compounds **SKLB-5A**. Compound **SKLB-5A** were purified by flash column chromatography (DCM/MeOH, 10:1).

*2-amino-1-(4-fluorobenzyl)-1H-pyrrolo[2,3-b]quinoxaline-3-carboxylic acid* ***(SKLB-5A)***

**SKLB-5A**

Dark yellow solid, mp 251.7-253.3, yield 36%. ^1^H-NMR (400 MHz, DMSO*-d*_6_), *δ* (ppm): 11.90 (s, 1H), 8.81 (s, 2H), 7.94 (d, *J* = 7.7 Hz, 1H), 7.89 (d, *J* = 7.7 Hz, 1H), 7.62 (dd, *J* = 7.7, 1.3 Hz, 1H), 7.55 (dd, *J* = 7.7, 1.3 Hz, 1H), 7.33 (m, 2H), 7.16 (m, 2H), 5.45 (s, 2H); ^13^C-NMR (100 MHz, DMSO*-d*_6_), *δ* (ppm): 160.8 (*J_CF_* = 243.3 Hz), 160.6, 155.6, 143.5, 137.1, 132.3, 129.7 (2), 128.2, 128.1, 126.6, 126.1, 125.3, 123.4, 116.0 (2), 115.8, 114.9, 61.7. HRMS(ESI)^+^ Calculated for C_18_H_13_FN_4_O_2_, Exact Mass: 336.1023, [M+Na]^+^: *m/z* 359.0920, found *m/z* 359.0920. Purity: 99.67% (HPLC, *t*_R_ = 5.78 min).

**3.5 The synthesis of compound SKLB-7A**

******

To a solution of Boc anhydride (1.1 equiv.) in DCM (0.10 M) the compounds **SKLB-4B** (1.0 equiv.) was added. The reaction mixture was stirred at room temperature for 1 h, evaporate the mixture in a rotary evaporator under vacuum to obtain compound **6**. To a solution of KOH in EtOH (75%) the **6** derivative was added. The reaction mixture was heated to 100 °C for 19 h, evaporate the mixture in a rotary evaporator under vacuum to obtain compounds **SKLB-7A**. Compound **SKLB-7A** were purified by flash column chromatography (DCM/MeOH, 10:1).

*2-((tert-butoxycarbonyl)amino)-1-(4-fluorobenzyl)-1H-pyrrolo[2,3-b]quinoxaline-3-carboxylic acid* ***(SKLB-7A)***

**SKLB-7A**

Light yellow solid, mp 231.7-233.3, yield 34%. ^1^H-NMR (400 MHz, DMSO*-d*_6_), *δ*(ppm): 11.02(s, 1H), 8.17 (d, *J* = 7 Hz, 1H), 8.10 (d, *J* = 7 Hz, 1H), 7.78 (m, 2H), 7.19 (m, 2H), 7.14 (m, 2H), 5.66 (s, 2H), 1.46(s, 9H); ^13^C-NMR (100 MHz, DMSO*-d*_6_), *δ* (ppm): 160.0, 159.2, 144.1, 142.2, 140.8, 137.6, 129.0, 128.3, 128.1, 127.6, 127.2, 126.2, 115.8, 114.5, 60.8, 56.5, 55.5, 55.5, 43.0, 19.0. HRMS(ESI)^+^ Calculated for C_23_H_21_N_4_O_4_, Exact Mass: 436.1547, [M+H]^+^: *m/z* 437.3721, found *m/z* 437.3738. Purity: 97.35% (HPLC, *t*_R_ = 12.0 min).

**3.6 General procedure for the synthesis of Compounds SKLB-11A-SKLB-11Q**

******

Different types of ammonia and alcohol derivatives and catalytic amounts of triethylamine were added to a THF solution containing ethyl cyanoacetate (**8**). The reaction mixture continues to be stirred at room temperature for 4 h, evaporate the mixture in a rotary evaporator under vacuum to obtain intermediates **9**. Intermediates **9** were purified by lash column chromatography (PE/EA, 15:1).

Different intermediates **9** (2.03 equiv.) was carefully added in portions to a 10 mL DME suspension containing sodium hydride (2.07 equiv.) and stirred at room temperature for 30 minutes. After the addition of compound **1** (1.0 equiv.), stirring was continued at room temperature for an additional 10 minutes, followed by heating to reflux for 1 h. Subsequently, the mixture was evaporated under reduced pressure, and cold 1 M hydrochloric acid was added to obtain a yellow crude product. The product was filtered and washed with water and ethanol, yielding different intermediates **10** .

To a solution of different intermediates **10** (1.0 equiv.) in 1 : 1 EtOH – toluene solution (0.09 M) different primary amines (4.0 equiv.) was added. The reaction mixture was heated to 130-160 °C for 4-12 h and cooled down. The formed solid was filtered off and washed with Et_2_O and EtOH to obtain the desired series compounds **SKLB-11**. Compounds **SKLB-11A**-**SKLB-11Q** were purified by flash column chromatography (petroleum ether/EtOAc, 1:1).

*Methyl 2-amino-1-(4-fluorobenzyl)-1H-pyrrolo[2,3-b]quinoxaline-3-carboxylate* ***(******SKLB-11A)***

**SKLB-11A**

Light yellow solid, mp 204.2-208.3, yield 56%. ^1^H-NMR (400 MHz, DMSO*-d*_6_), *δ* (ppm): 8.41 (s, 2H), 7.96 (dd, *J* = 8.3, 1.3 Hz, 1H), 7.86 (dd, *J* = 8.3, 1.3 Hz, 1H), 7.58 (m, 1H), 7.51 (m, 1H), 7.32 (m, 2H), 7.15 (t, *J* = 8.9 Hz, 2H), 5.49 (s, 2H), 3.83 (s, 3H); ^13^C-NMR (100 MHz, DMSO*-d*_6_), *δ* (ppm): 165.6, 160.7, 160.1, 142.4, 141.9, 141.1, 137.2, 132.8, 129.5 (2), 129.4, 128.0, 127.9, 126.9, 116.0 (2), 115.8, 81.2, 50.8. HRMS(ESI)^+^ Calculated for C_19_H_15_FN_4_O_2_, Exact Mass: 350.1179, [M+H]^+^: *m/z* 351.1252, found *m/z* 351.1249. Purity: 95.28% (HPLC, *t*_R_ = 12.05 min).

*Ethyl 2-amino-1-(4-fluorobenzyl)-1H-pyrrolo[2,3-b]quinoxaline-3-carboxylate* ***(SKLB-11B )***

**SKLB-11B**

Light yellow solid, mp 214.5-217.3, yield 52%. ^1^H-NMR (400 MHz, DMSO*-d*_6_), *δ* (ppm): 8.40 (s, 2H), 7.95 (dd, *J* = 8.3, 1.3 Hz, 1H), 7.85 (dd, *J* = 8.3, 1.3 Hz, 1H), 7.57 (m, 1H), 7.51 (m, 1H), 7.30 (m, 2H), 7.15 (m, 2H), 5.49 (s, 2H), 4.34 (q, *J* = 7.0 Hz, 2H), 1.36 (t, *J* = 7.0 Hz, 3H); ^13^C-NMR (100 MHz, DMSO*-d*_6_), *δ* (ppm): 165.2, 160.7, 160.1, 142.4, 142.0, 141.1, 137.2, 132.9, 129.5 (2), 129.4, 128.1, 127.9, 126.8, 126.1, 116.0 (2), 81.4, 59.2, 42.4, 15.3. HRMS(ESI)^+^ Calculated for C_20_H_17_FN_4_O_2_, Exact Mass: 364.1336, [M+H]^+^: *m/z* 365.1408, found *m/z* 365.1409. Purity: 99.11% (HPLC, *t*_R_ = 14.35 min).

*Isopropyl 2-amino-1-(4-fluorobenzyl)-1H-pyrrolo[2,3-b]quinoxaline-3-carboxylate* ***(SKLB-11C)***

**SKLB-11C**

Light yellow solid, mp 235.5-239.3, yield 51%. ^1^H-NMR (400 MHz, DMSO*-d*_6_), *δ* (ppm): 8.35 (s, 2H), 7.92 (dd, *J* = 8.0, 0.8 Hz, 1H), 7.86 (dd, *J* = 8.0, 0.8 Hz, 1H), 7.57 (m, 1H), 7.51 (m, 1H), 7.30 (m, 2H), 7.15 (t, *J* = 8.9 Hz, 2H), 5.49 (s, 2H), 5.17 (m, 1H), 1.37 (d, *J* = 6.3 Hz, 6H); ^13^C-NMR (100 MHz, DMSO*-d*_6_), *δ* (ppm): 164.9, 160.0, 142.4, 142.1, 141.1, 137.2, 132.9, 129.5 (2), 129.4, 128.3, 127.8, 126.8, 126.0, 116.0 (2), 81.6, 66.5, 42.4, 22.6 (2). HRMS(ESI)^+^ Calculated for C_21_H_19_FN_4_O_2_, Exact Mass: 378.1492, [M+H]^+^: *m/z* 379.1565, found *m/z* 379.1562. Purity: 97.63% (HPLC, *t*_R_ = 13.81 min).

*Tert-butyl 2-amino-1-(4-fluorobenzyl)-1H-pyrrolo[2,3-b]quinoxaline-3-carboxylate* ***(SKLB-11D)***

**SKLB-11D**

Dark yellow solid, mp 241.6-245.2, yield 46%. ^1^H-NMR (400 MHz, DMSO*-d*_6_), *δ* (ppm): 8.32 (s, 2H), 7.87 (m, 2H), 7.57 (m, 2H), 7.50 (m, 2H), 7.30 (m, 2H), 7.15 (m, 2H), 5.47 (s, 2H), 1.61 (s, 9H); ^13^C-NMR (100 MHz, DMSO*-d*_6_), *δ* (ppm): 165.0, 160.7, 159.9, 142.3, 142.2, 141.2, 137.2, 132.9, 129.5, 129.4, 128.3, 127.8, 126.7, 125.9, 116.0 (2), 82.5, 79.4, 42.3, 28.9 (3). HRMS(ESI)^+^ Calculated for C_22_H_21_FN_4_O_2_, Exact Mass: 392.1649, [M+H]^+^: *m/z* 393.1721, found *m/z* 393.1725. Purity: 94.67% (HPLC, *t*_R_ = 14.35 min).

*Phenyl 2-amino-1-(4-fluorobenzyl)-1H-pyrrolo[2,3-b]quinoxaline-3-carboxylate* ***(SKLB-11E)***

**SKLB-11E**

Light yellow solid, mp 221.0-223.3, yield 44%. ^1^H-NMR (400 MHz, DMSO*-d*_6_), *δ* (ppm): 8.63 (s, 2H), 8.10 (d, *J* = 7.8, 1H), 7.91 (d, *J* = 7.8, 1H), 7.81 (d, *J* = 7.8, 2H), 7.63 (m, 1H), 7.54 (m, 1H), 7, 40 (d, *J* = 7.8, 2H), 7.34 (m, 2H), 7.16 (m, 2H), 7.08 (m, 1H), 5.51 (s, 2H). ^13^C-NMR (100 MHz, DMSO*-d*_6_), *δ* (ppm): 163.5, 163.2, 160.7, 159.4, 143.2, 141.6, 139.8, 137.3, 132.9, 129.6 (3), 129.5, 128.1, 127.5, 127.2, 126.0, 123.2, 119.3 (2), 116.0, 115.8, 83.2, 42.4. HRMS(ESI)^+^ Calculated for C_24_H_17_FN_4_O_2_, Exact Mass: 412.1336, [M+H]^+^: *m/z* 413.1573, found *m/z* 413.1589. Purity: 99.85% (HPLC, *t*_R_ = 4.94 min).

*Tert-butyl 2-amino-1-benzyl-1H-pyrrolo[2,3-b]quinoxaline-3-carboxylate* ***(SKLB-11F)***

**SKLB-11F**

Bright yellow solid, mp 241.2-243.8, yield 36%. ^1^H-NMR (400 MHz, DMSO*-d*_6_), *δ* (ppm): 8.28 (s, 2H), 7.89 (d, *J* = 7.4 Hz, 1H), 7.83 (d, *J* = 7.4 Hz, 1H), 7.56 (m, 1H), 7.48 (m, 1H), 7.31 (m, 2H), 7.26 (m, 1H), 7.21 (m, 2H), 5.49 (s, 2H), 1.61 (s, 9H); ^13^C-NMR (100 MHz, DMSO*-d*_6_), *δ* (ppm): 165.0, 160.0, 142.4, 142.2, 141.2, 137.2, 136.7, 129.1 (2), 128.3, 127.8 (2), 127.2 (2), 126.7, 125.8, 82.4, 79.4, 43.0, 28.9 (3). HRMS(ESI)^+^ Calculated for C_22_H_22_N_4_O_2_, Exact Mass: 374.1743, [M+H]^+^: *m/z* 375.1816, found *m/z* 375.1819. Purity: 95.50% (HPLC, *t*_R_ = 13.01 min).

*Tert-butyl 2-amino-1-(4-methylbenzyl)-1H-pyrrolo[2,3-b]quinoxaline-3-carboxylate* ***(SKLB-11G)***

**SKLB-11G**

Light yellow solid, mp 250.7-253.8, yield 28%. ^1^H-NMR (400 MHz, DMSO*-d*_6_), *δ* (ppm): 8.29 (s, 2H), 7.90 (d, *J* = 7 Hz, 1H), 7. 78(d, *J* = 7 Hz, 1H), 7.53 (m, 1H), 7.46 (m, 1H), 7.26(d, *J* = 7 Hz, 1H), 7.15(m, 1H), 7.02(m, 1H), 6.31(d, *J* = 7 Hz, 1H), 5.42 (s, 2H), 2.47 (s, 3H), 1.63 (s, 9H). ^13^C-NMR (100 MHz, DMSO*-d*_6_), *δ* (ppm): 165.1, 160.3, 142.3, 141.2, 137.2, 135.8, 134.5, 130.6, 128.3, 127.7, 127.4, 126.7, 126.5, 125.8, 123.9, 82.3, 79.4 (2), 41.6, 29.0 (3), 19.2. HRMS(ESI)^+^ Calculated for C_23_H_24_N_4_O2, Exact Mass: 388.1899, [M+H]^+^: *m/z* 389.1972, found *m/z* 389.1977 . Purity: 99.04% (HPLC, *t*_R_ = 12.27 min).

*Tert-butyl 2-amino-1-(4-methoxybenzyl)-1H-pyrrolo[2,3-b]quinoxaline-3-carboxylate* ***(SKLB-11H)***

**SKLB-11H**

Light yellow solid, mp 261.2-266.9, yield 46%. ^1^H-NMR (400 MHz, DMSO*-d*_6_), *δ* (ppm): 8.27 (s, 2H), 7.87 (td, *J* = 7.8, 1.2 Hz, 2H), 7.56 (m, 1H), 7.50 (m, 1H), 7.22 (d, *J* = 8.7 Hz, 2H), 6.87(d, *J* = 8.7 Hz, 2H), 5.42 (s, 2H), 3.68 (s, 3H), 1.60 (s, 9H); ^13^C-NMR (100 MHz, DMSO*-d*_6_), *δ* (ppm): 165.0, 159.9, 159.1, 142.3, 142.2, 141.1, 137.2, 128.9 (2), 128.6, 128.3, 127.8, 126.7, 125.8, 114.4 (2), 82.4, 79.4, 55.5, 42.5, 28.9 (3). HRMS(ESI)^+^ Calculated for C_23_H_20_N_4_O_3_, Exact Mass: 404.1848, [M+H]^+^: *m/z* 405.1921, found *m/z* 405.1919. Purity: 96.40% (HPLC, *t*_R_ = 16.14 min).

*2-amino-1-(4-fluorobenzyl)-1H-pyrrolo[2,3-b]quinoxaline-3-carboxamide* ***(SKLB-11I)***

**SKLB-11I**

Bright yellow solid, mp 215.7-219.1, yield 26%. ^1^H-NMR (400 MHz, DMSO*-d*_6_), *δ* (ppm): 8.65 (s, 2H), 7.95 (dd, *J* = 7.7, 0.8 Hz, 1H), 7.88 (dd, *J* = 7.7, 0.8 Hz, 1H), 7.60 (m, 2H), 7.53 (m, 2H), 7.32 (m, 2H), 7.15 (t, *J* = 8.9 Hz, 2H), 5.43 (s, 2H); ^13^C-NMR (100 MHz, DMSO*-d*_6_), *δ* (ppm): 160.7, 160.1, 144.1, 142.2, 140.9, 137.6, 132.6, 129.6 (2), 129.5, 128.0, 127.7, 127.3, 126.2, 116.0, 115.7, 60.9, 42.8. HRMS(ESI)^+^ Calculated for C_18_H_14_FN_5_O, Exact Mass: 335.1182, [M+H]^+^: *m/z* 336.3261, found *m/z* 336.3262. Purity: 97.56% (HPLC, *t*_R_ = 14.22 min).

*2-amino-1-(4-fluorobenzyl)-N-methyl-1H-pyrrolo[2,3-b]quinoxaline-3-carboxamide* ***(******SKLB-11J)***

**SKLB-11J**

Bright yellow solid, mp 227.1-229.3, yield 52%. ^1^H-NMR (400 MHz, DMSO*-d*_6_), *δ* (ppm): 8.63 (s, 2H), 8.02 (q, *J* = 4.6 Hz, 1H), 7.93 (dd, *J* = 8.2, 1.3 Hz, 1H), 7.87 (dd, *J* = 8.2, 1.3 Hz, 1H), 7.57 (dd, *J* = 7.6, 1.3 Hz, 1H), 7.49 (dd, *J* = 7.6, 1.3 Hz, 1H), 7.34 (m, 2H), 7.14 (m, 2H), 5.47 (s, 2H), 2.94 (d, *J* = 4.6 Hz, 2H); ^13^C-NMR (100 MHz, DMSO*-d*_6_), *δ* (ppm): 165.9, 160.7, 159.1, 143.0, 141.9, 140.1, 137.1, 133.2, 129.7 (2), 128.1, 127.2, 127.0, 126.1, 115.9 (2), 83.0, 42.4, 25.5. HRMS(ESI)^+^ Calculated for C_19_H_16_FN_5_O. Exact Mass: 349.1339, [M+H]^+^: *m/z* 350.1417, found *m/z* 350.1411. Purity: 99.84% (HPLC, *t*_R_ =16.44 min).

*2-amino-N-ethyl-1-(4-fluorobenzyl)-1H-pyrrolo[2,3-b]quinoxaline-3-carboxamide* ***(SKLB-11K)***

**SKLB-11K**

Bright yellow solid, mp 231.5-236.7, yield 74%. ^1^H-NMR (400 MHz, DMSO*-d*_6_), *δ* (ppm): 8.52 (s, 2H), 8.13 (t, *J* = 5.8 Hz, 1H), 7.94 (dd, *J* = 8.2, 1.1 Hz, 1H), 7.88 (dd, *J* = 8.2, 1.3 Hz, 1H), 7.58 (m, 1H), 7.50 (m, 1H), 7.33 (m, 2H), 7.15 (m, 2H), 5.48 (s, 2H), 3.43 (m, 2H), 1.22 (t, *J* = 7.2 Hz, 1H); ^13^C-NMR (100 MHz, DMSO*-d*_6_), *δ* (ppm): 165.3, 160.7, 159.1, 142.9, 141.8, 140.1, 137.1, 133.0, 129.5 (2), 128.1, 127.3, 127.1, 125.6, 116.0 (2), 83.0, 41.6, 33.3, 16.0. HRMS(ESI)^+^ Calculated for C_20_H_18_FN_5_O, Exact Mass: 363.1495, [M+H]^+^: *m/z* 364.1574, found *m/z* 364.1569. Purity: 99.42% (HPLC, *t*_R_ = 5.60 min).

*2-amino-N-butyl-1-(4-fluorobenzyl)-1H-pyrrolo[2,3-b]quinoxaline-3-carboxamide* ***(SKLB-11L)***

**SKLB-11L**

Bright yellow solid, mp 258.8-262.3, yield 51%. ^1^H-NMR (400 MHz, DMSO*-d*_6_), *δ* (ppm): 8.50 (s, 2H), 8.22-8.14 (m, 1H), 7.91-7.82 (m, 2H), 7.60-7.51 (m, 1H), 7.51-7.43 (m, 1H), 7.35-7.27 (m, 2H), 7.17-7.08 (m, 2H), 5.46 (s, 2H), 3.43-3.34 (m, 2H), 1.61-1.50 (m, 2H), 1.48-1.34 (m, 2H), 0.97-0.89 (m, 3H). ^13^C-NMR (100 MHz, DMSO*-d*_6_), *δ* (ppm): 165.3, 159.2, 143.0, 141.9, 140.1, 137.1, 136.9, 129.1 (2), 128.1, 127.8, 127.2, 127.0 (2), 125.5, 83.0, 42.97, 38.20, 29.86, 29.26, 22.34, 14.46. HRMS(ESI)^+^ Calculated for C_22_H_22_FN_5_O, Exact Mass: 391.1808, [M+Na]^+^: *m/z* 414.1706, found *m/z* 414.1709. Purity: 97.35% (HPLC, *t*_R_ = 8.57 min).

*2-amino-N-cyclopropyl-1-(4-fluorobenzyl)-1H-pyrrolo[2,3-b]quinoxaline-3-carboxamide* ***(SKLB-11M)***

**SKLB-11M**

White gray solid, mp 248.3-251.6, yield 32%. ^1^H-NMR (4 00 MHz, DMSO*-d*_6_), *δ* (ppm): 8.19 (d, *J* = 3.1 Hz, 1H), 7.90 (s, 2H), 7.58 (t, *J* = 7.3 Hz, 1H), 7.49 (t, *J* = 7.3 Hz, 1H), 7.30 (m, 2H), 7.15 (m, 2H), 5.45 (s, 2H), 2.85(m, 1H), 0.78 (m, 2H), 0.60 (m, 2H); ^13^C-NMR (100 MHz, DMSO*-d*_6_), *δ* (ppm): 166.4, 159.0, 142.9, 141.8, 140.0, 137.1, 133.0 129.5 (2), 129.4, 128.1, 127.4, 127.0, 125.6, 116.0 (2), 82.9, 42.3, 22.3, 7.0 (2). HRMS(ESI)^+^ Calculated for C_21_H_18_FN_5_O, Exact Mass: 375.1495, [M+H]^+^: *m/z* 376.1574, found *m/z* 376.1564. Purity: 95.44% (HPLC, *t*_R_ = 10.82 min).

*2-amino-1-benzyl-N-pentyl-1H-pyrrolo[2,3-b]quinoxaline-3-carboxamide* ***(SKLB-11N)***

**SKLB-11N**

Light yellow solid, mp 261.3-266.7, yield 51%. ^1^H-NMR (400 MHz, DMSO*-d*_6_), *δ* (ppm): 8.52 (s, 2H), 8.25-8.18 (m, 1H), 7.90 (d, *J* = 8.3 Hz, 1H), 7.87 (d, *J* = 8.2 Hz, 1H), 7.62-7.53 (m, 1H), 7.53-7.45 (m, 1H), 7.36-7.29 (m, 2H), 7.29-7.21 (m, 3H), 5.50 (s, 2H), 3.40 (q, *J* = 6.6 Hz, 2H), 1.66-1.54 (m, 2H), 1.46-1.30 (m, 4H), 0.95-0.87 (m, 3H).^13^C-NMR (100 MHz, DMSO*-d*_6_), *δ* (ppm): 165.2, 159.1, 143.0, 141.8, 140.1, 137.1, 136.8, 129.0 (2), 128.0, 127.8, 127.1, 126.9 (3), 125.2, 83.0, 42.9, 38.2, 29.8, 29.2, 22.3, 14.4. HRMS(ESI)^+^ Calculated for C_23_H_25_N_5_O, Exact Mass: 387.2059, [M+H]^+^: *m/z* 388.2137, found *m/z* 388.2153. Purity: 96.44% (HPLC, *t*_R_ = 9.85 min).

*2-amino-1-(4-bromobenzyl)-N-pentyl-1H-pyrrolo[2,3-b]quinoxaline-3-carboxamide* ***(SKLB-11O)***

**SKLB-11O**

Light yellow solid, mp 312.4-315.8, yield 48%. ^1^H-NMR (400 MHz, DMSO*-d*_6_), *δ* (ppm): 8.49 (s, 2H), 8.25-8.17 (m, 1H), 7.94-7.83 (m, 2H), 7.63-7.55 (m, 1H), 7.53 (d, *J* = 1.9 Hz, 1H), 7.51 (s, 1H), 7.54-7.45 (m, 1H), 7.20 (d, *J* = 8.3 Hz, 2H), 5.46 (s, 2H), 3.44-3.35 (m, 2H), 1.66-1.54 (m, 2H), 1.46-1.31 (m, 4H), 0.95-0.87 (m, 3H). ^13^C-NMR (100 MHz, DMSO*-d*_6_), *δ* (ppm): 165.2, 159.0, 142.9, 141.9, 140.1, 137.1, 136.3, 131.9 (3), 129.5 (2), 128.1, 127.2, 127.1, 125.6, 120.9, 83.1, 38.2, 29.9, 29.3, 22.3, 14.5. HRMS(ESI)^+^ Calculated for C_23_H_24_BrN_5_O, Exact Mass: 465.1164, [M+H]^+^: *m/z* 466.1242, found *m/z* 466.1228. Purity: 99.37% (HPLC, *t*_R_ = 5.49 min).

*2-amino-1-(3-bromobenzyl)-N-pentyl-1H-pyrrolo[2,3-b]quinoxaline-3-carboxamide* ***(SKLB-11P)***

**SKLB-11P**

Light yellow solid, mp 315.5-319.2, yield 42%. ^1^H-NMR (400 MHz, DMSO*-d*_6_), *δ* (ppm): 8.51 (s, 2H), 8.21 (m, 1H), 7.91 (d, *J* = 7.4 Hz, 1H), 7.87 (d, *J* = 7.2 Hz, 1H), 7.61-7.46 (m, 4H), 7.28 (m, 1H), 7.18 (d, *J* = 7.8 Hz, 1H), 5.49 (s, 2H), 3.42-3.37 (m, 2H), 1.61-1.56 (m, 2H), 1.41-1.34 (m, 4H), 0.92 (m, 3H). ^13^C-NMR (100 MHz, DMSO*-d*_6_), *δ* (ppm): 165.2, 159.0, 142.9, 141.9, 140.1, 139.6, 137.1, 131.3, 130.8, 130.3, 128.1, 127.2, 127.1, 126.2, 125.6, 122.2, 83.1, 42.45, 38.2, 29.9, 29.3, 22.3, 14.5. HRMS(ESI)^+^ Calculated for C_23_H_24_BrN_5_O, Exact Mass: 465.1164, [M+Na]+: m/z 488.1062, found *m/z* 488.1066. Purity: 99.79% (HPLC, *t*_R_ = 5.10 min).

*2-amino-1-(3-chlorobenzyl)-N-pentyl-1H-pyrrolo[2,3-b]quinoxaline-3-carboxamide* ***(******SKLB-11Q)***

**SKLB-11Q**

Light yellow solid, mp 258.7-261.3, yield 51%. ^1^H-NMR (400 MHz, DMSO*-d*_6_), *δ* (ppm): 8.49 (s, 2H), 8.24-8.17 (m, 1H), 7.91 (dd, *J* = 8.3, 1.5 Hz, 1H), 7.87 (dd, *J* = 8.2, 1.5 Hz, 1H), 7.63-7.54 (m, 1H), 7.54-7.46 (m, 1H), 7.39-7.30 (m, 3H), 7.18-7.10 (m, 1H), 5.48 (s, 2H), 3.45-3.35 (m, 2H), 1.66-1.55 (m, 2H), 1.46-1.31 (m, 4H), 0.96-0.88 (m, 3H). ^13^C-NMR (100 MHz, DMSO*-d*_6_), *δ* (ppm): 165.2, 159.0, 142.9, 141.9, 140.1, 139.4, 137.1, 133.6, 131.0, 128.0, 127.9, 127.3, 127.2, 127.0, 125.8, 125.6, 83.0, 42.5, 38.2, 29.8, 29.2, 22.3, 14.4. HRMS(ESI)^+^ Calculated for C_23_H_24_ClN_5_O, Exact Mass: 421.1669, [M+H]^+^: *m/z* 422.1748, found *m/z* 422.1738. Purity: 96.57% (HPLC, *t*_R_ = 9.41 min).

1. **NMR, HRMS and HPLC spectra**


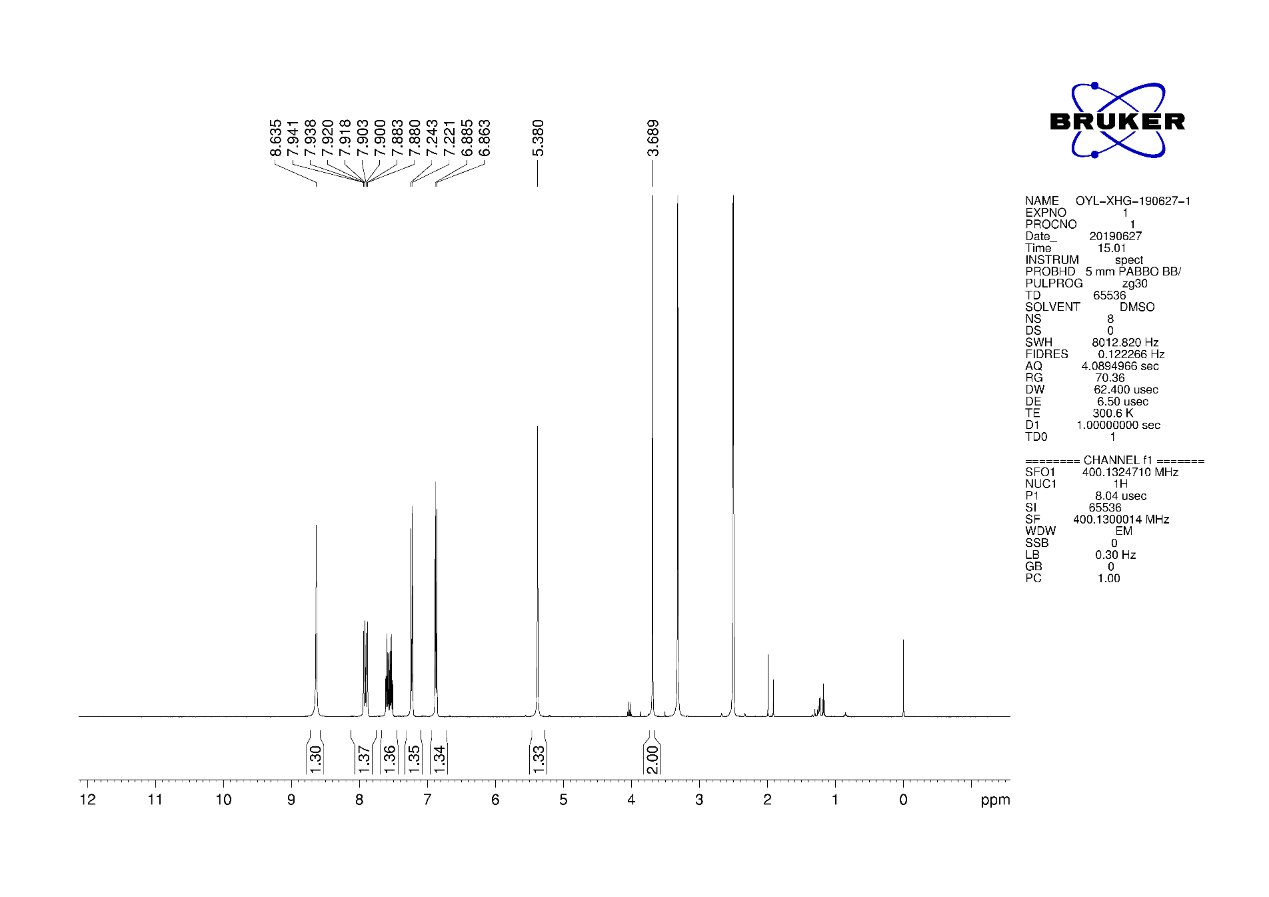

^1^H NMR Spectrum of **SKLB-4A**


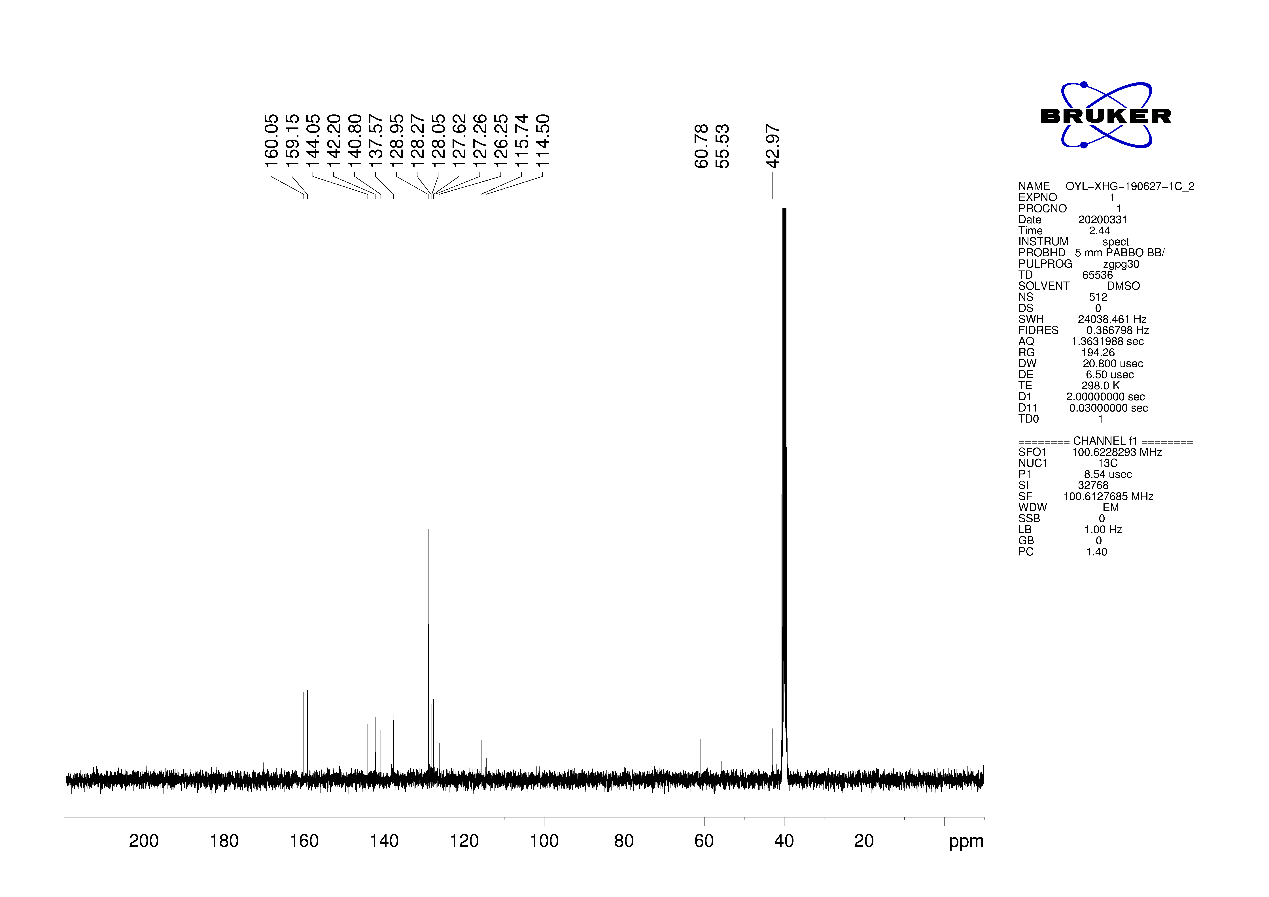

^13^C NMR Spectrum of **SKLB-4A**





^1^H NMR Spectrum of **SKLB-4B**





^13^C NMR Spectrum of **SKLB-4B**




^1^H NMR Spectrum of **SKLB-4C**




^13^C NMR Spectrum of **SKLB-4C**




^1^H NMR Spectrum of **SKLB-4D**




^13^C NMR Spectrum of **SKLB-4D**




^1^H NMR Spectrum of **SKLB-4E**




^13^C NMR Spectrum of **SKLB-4E**


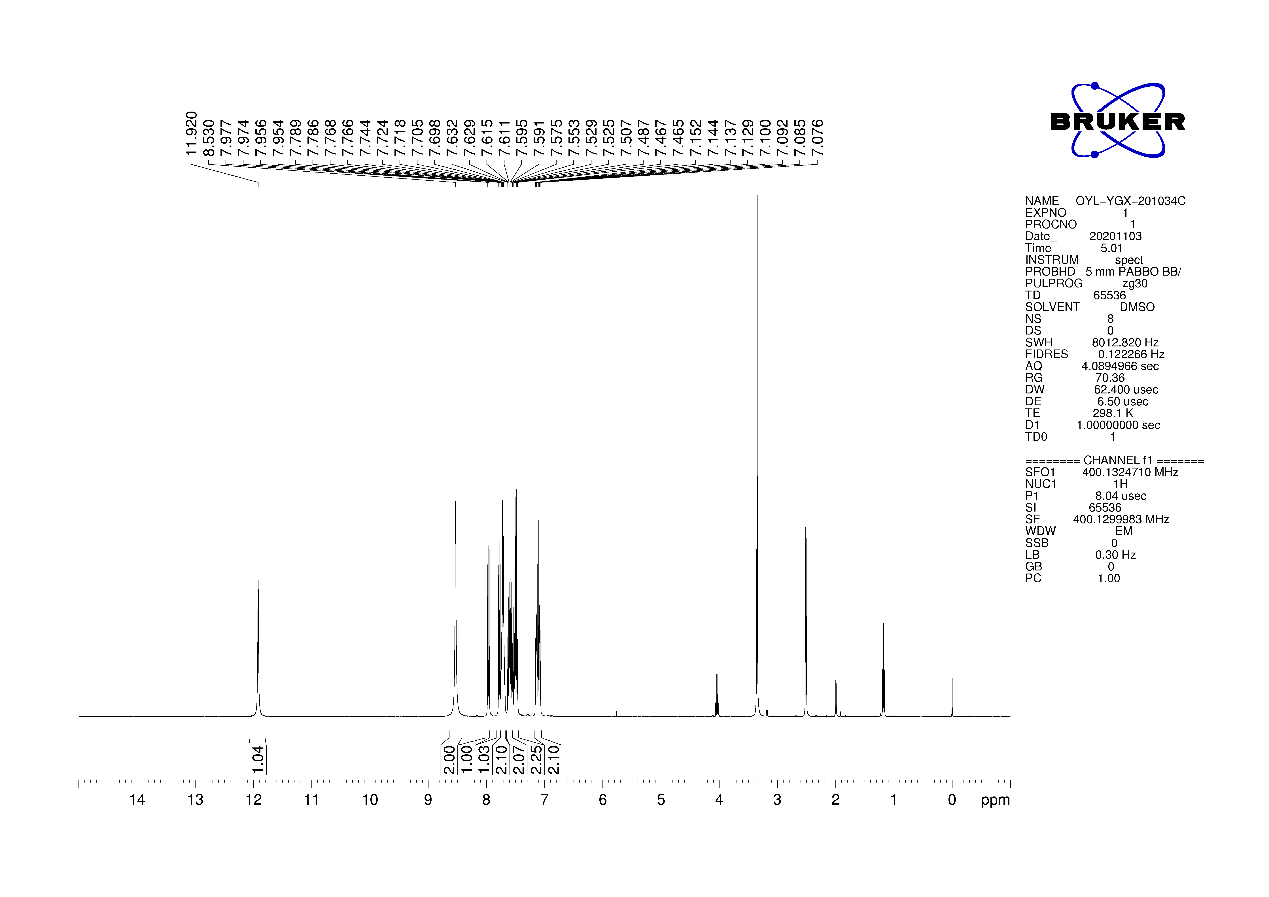

^1^H NMR Spectrum of **SKLB-4F**


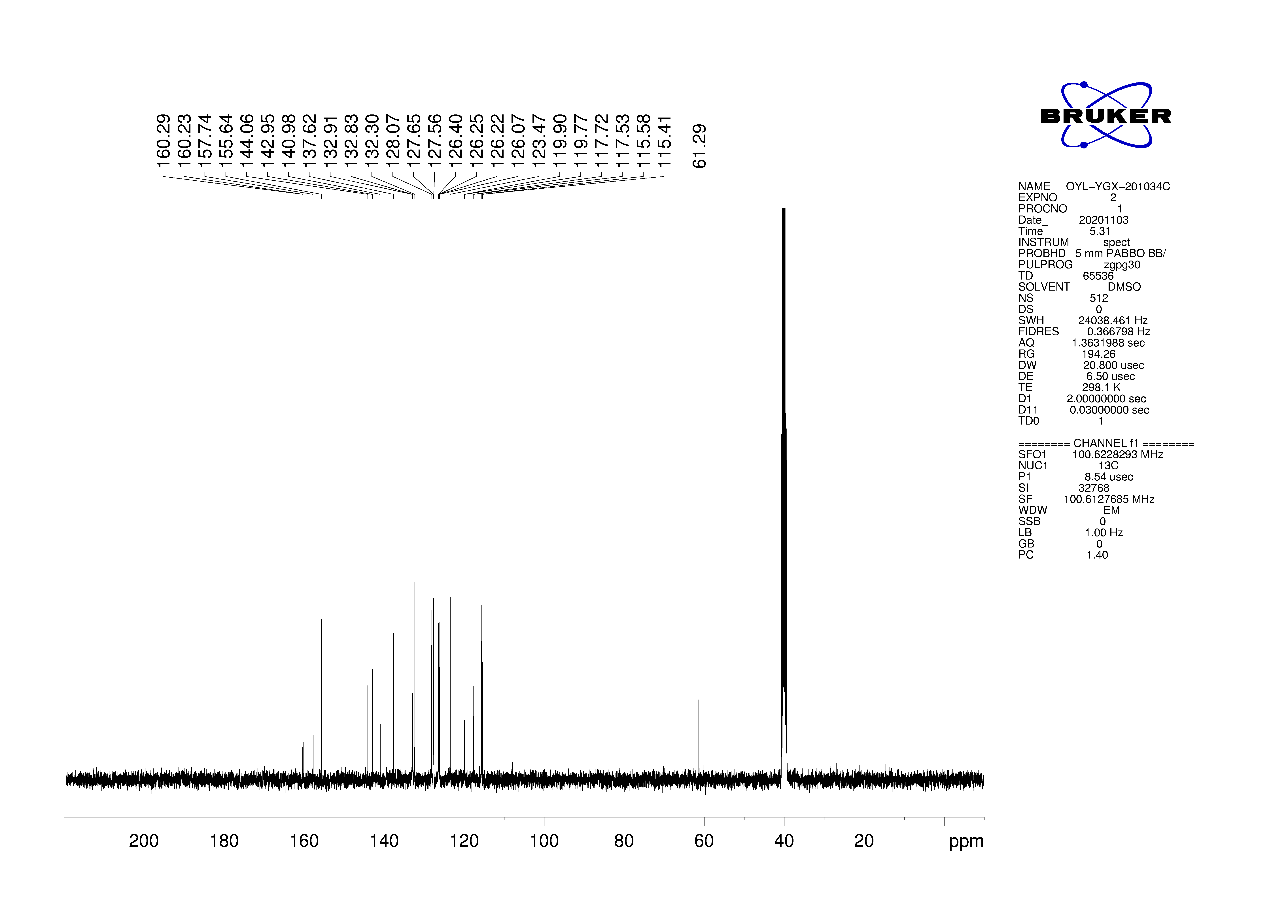

^13^C NMR Spectrum of **SKLB-4F**





^1^H NMR Spectrum of **SKLB-5A**



^13^C NMR Spectrum of **SKLB-5A**


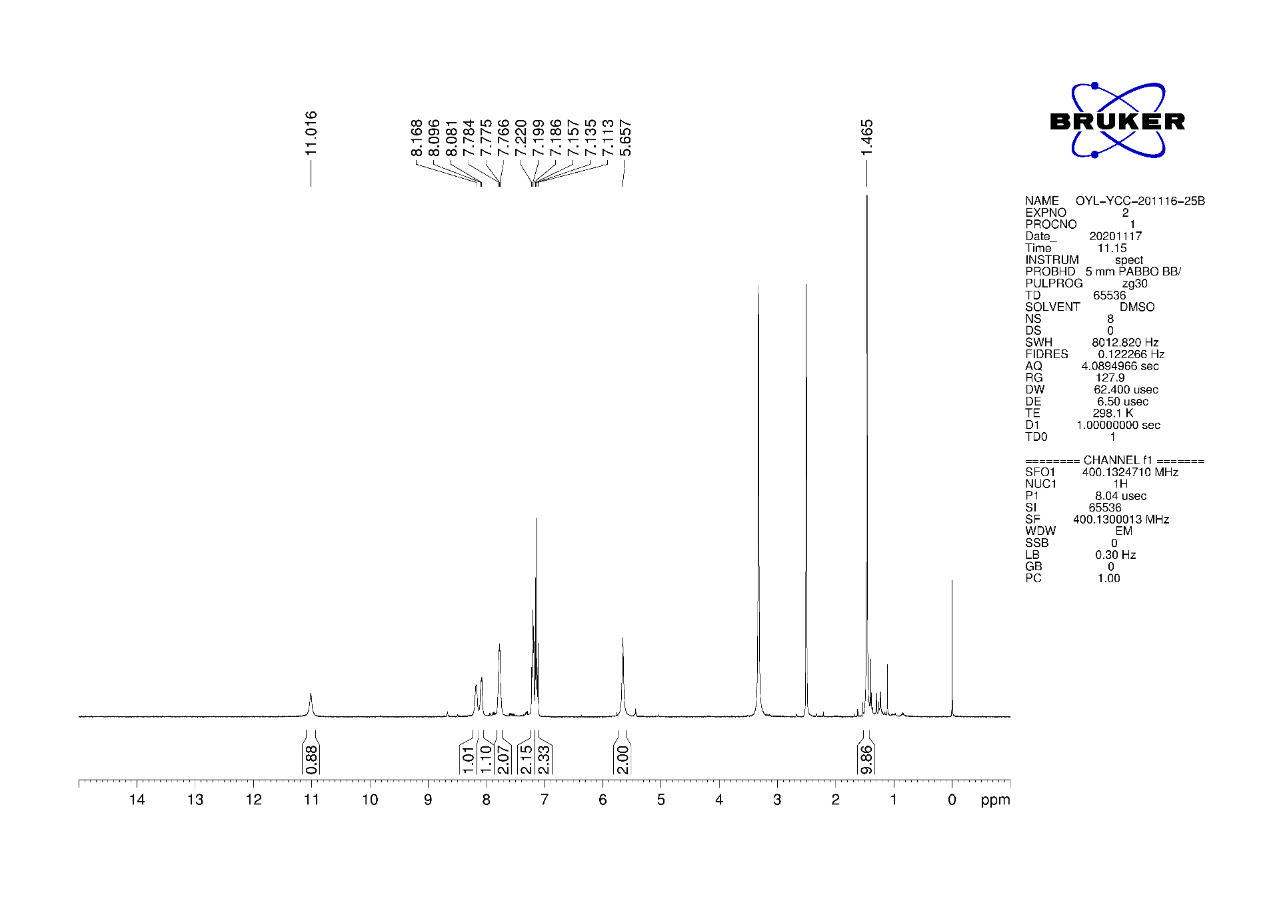

^1^H NMR Spectrum of **SKLB-7A**


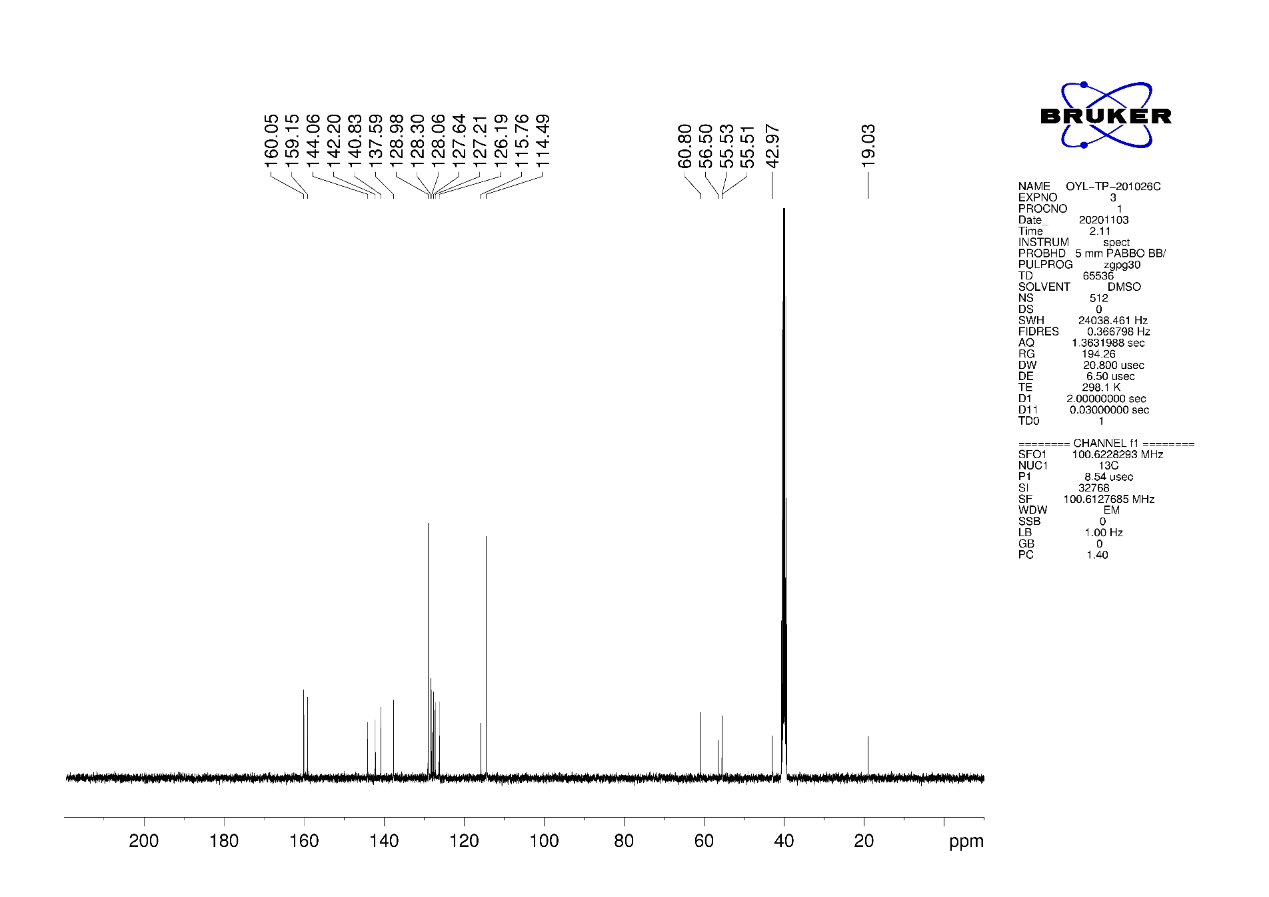

^13^C NMR Spectrum of **SKLB-7A**


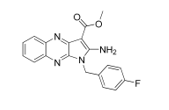

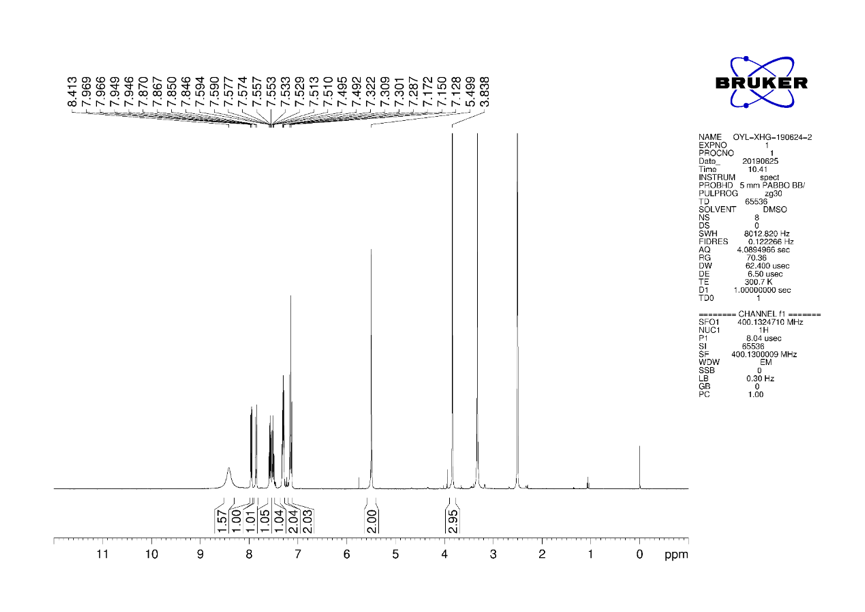


^1^H NMR Spectrum of **SKLB-11A**





^13^C NMR Spectrum of **SKLB-11A**


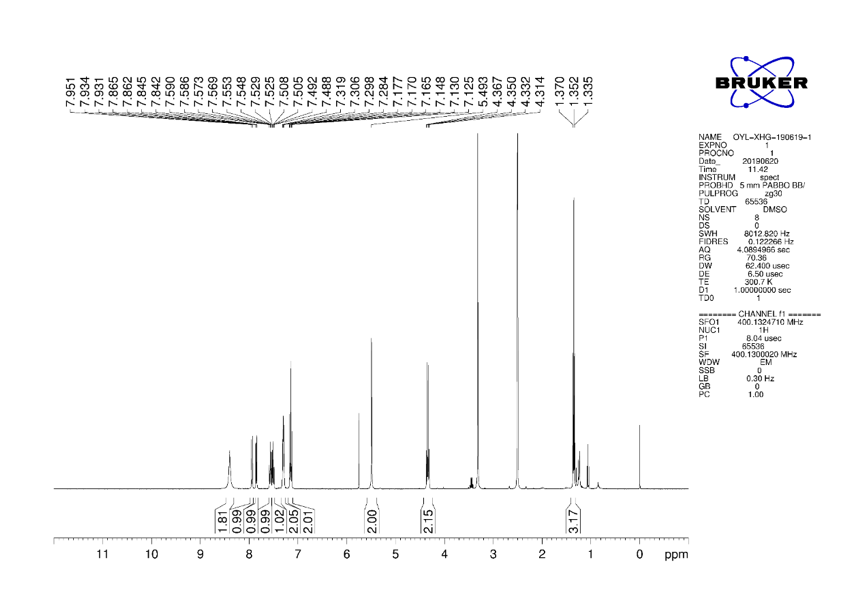

^1^H NMR Spectrum of **SKLB-11B**





^13^C NMR Spectrum of **SKLB-11B**





^1^H NMR Spectrum of **SKLB-11C**





^13^C NMR Spectrum of **SKLB-11C**





^1^H NMR Spectrum of **SKLB-11D**





^13^C NMR Spectrum of **SKLB-11D**




^1^H NMR Spectrum of **SKLB-11E**




^13^C NMR Spectrum of **SKLB-11E**





^1^H NMR Spectrum of **SKLB-11F**





^13^C NMR Spectrum of **SKLB-11F**





^1^H NMR Spectrum of **SKLB-11G**





^13^C NMR Spectrum of **SKLB-11G**





^1^H NMR Spectrum of **SKLB-11H**





^13^C NMR Spectrum of **SKLB-11H**





^1^H NMR Spectrum of **SKLB-11I**





^13^C NMR Spectrum of **SKLB-11I**


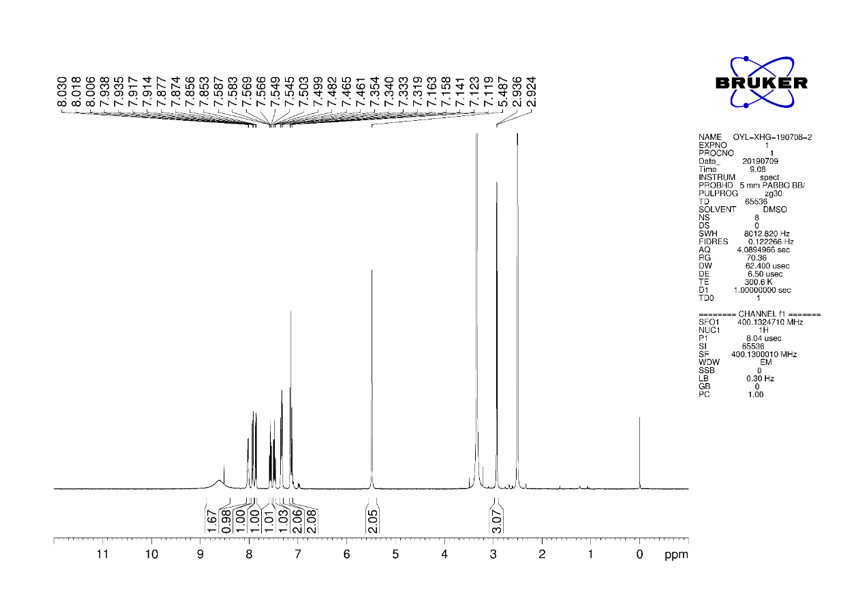

^1^H NMR Spectrum of **SKLB-11J**





^13^C NMR Spectrum of **SKLB-11J**


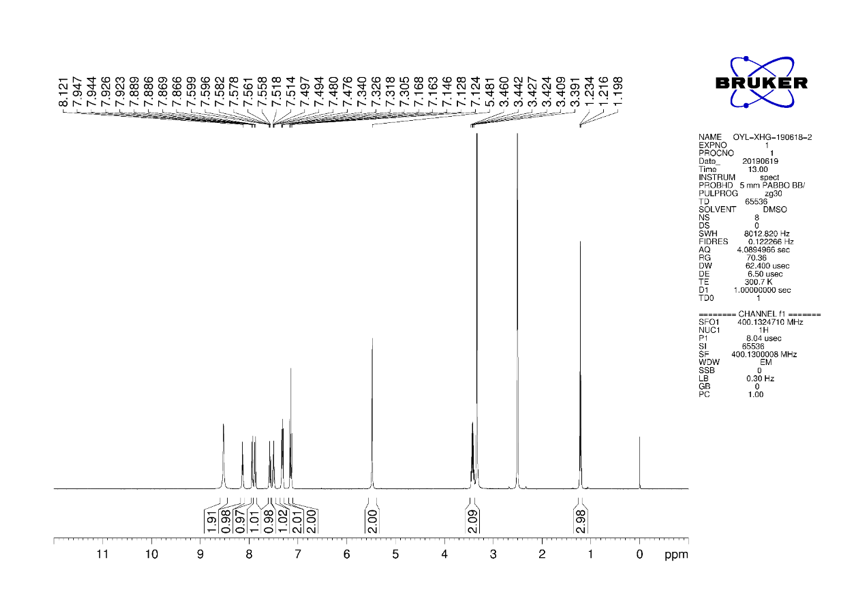

^1^H NMR Spectrum of **SKLB-11K**





^13^C NMR Spectrum of **SKLB-11K**


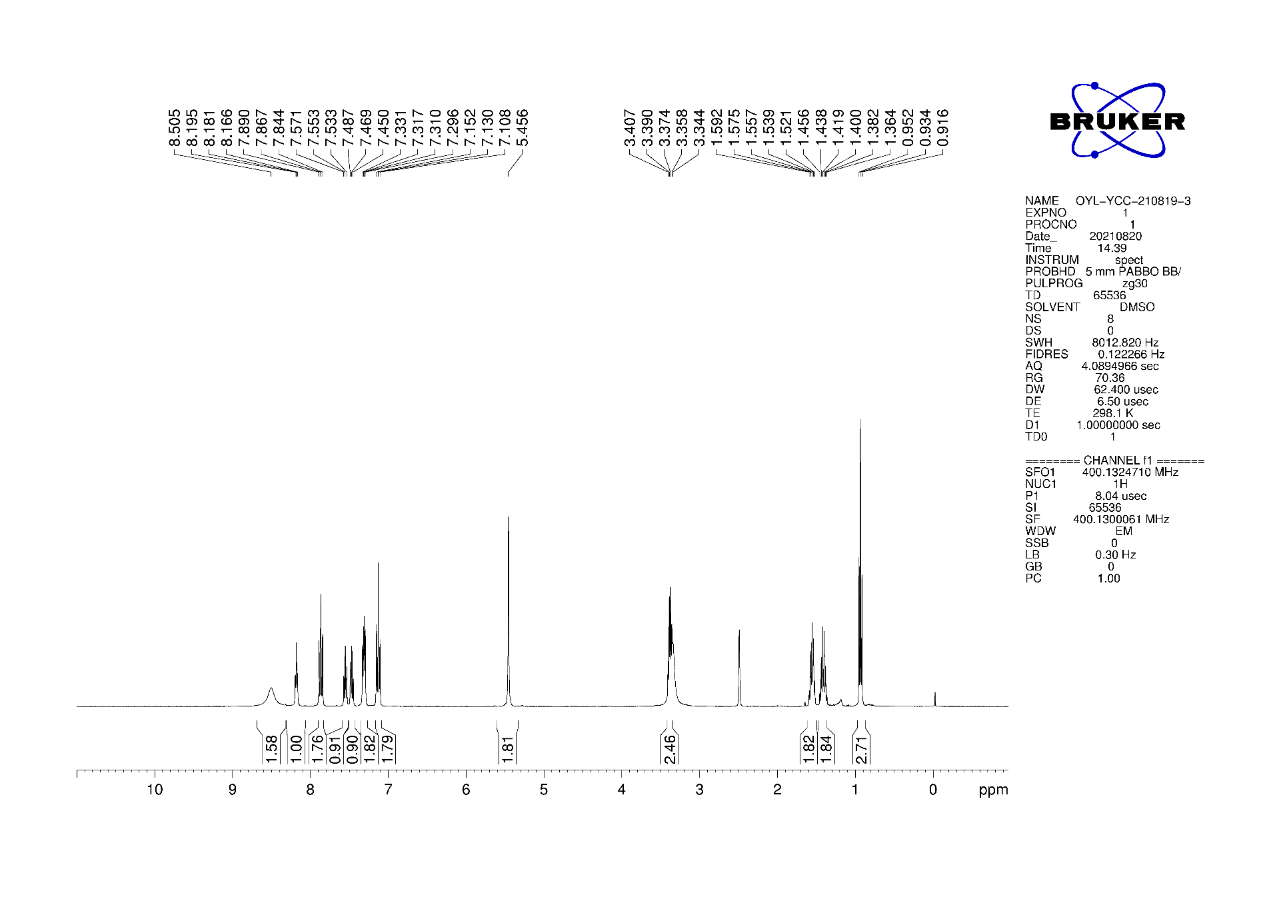

^1^H NMR Spectrum of **SKLB-11L**


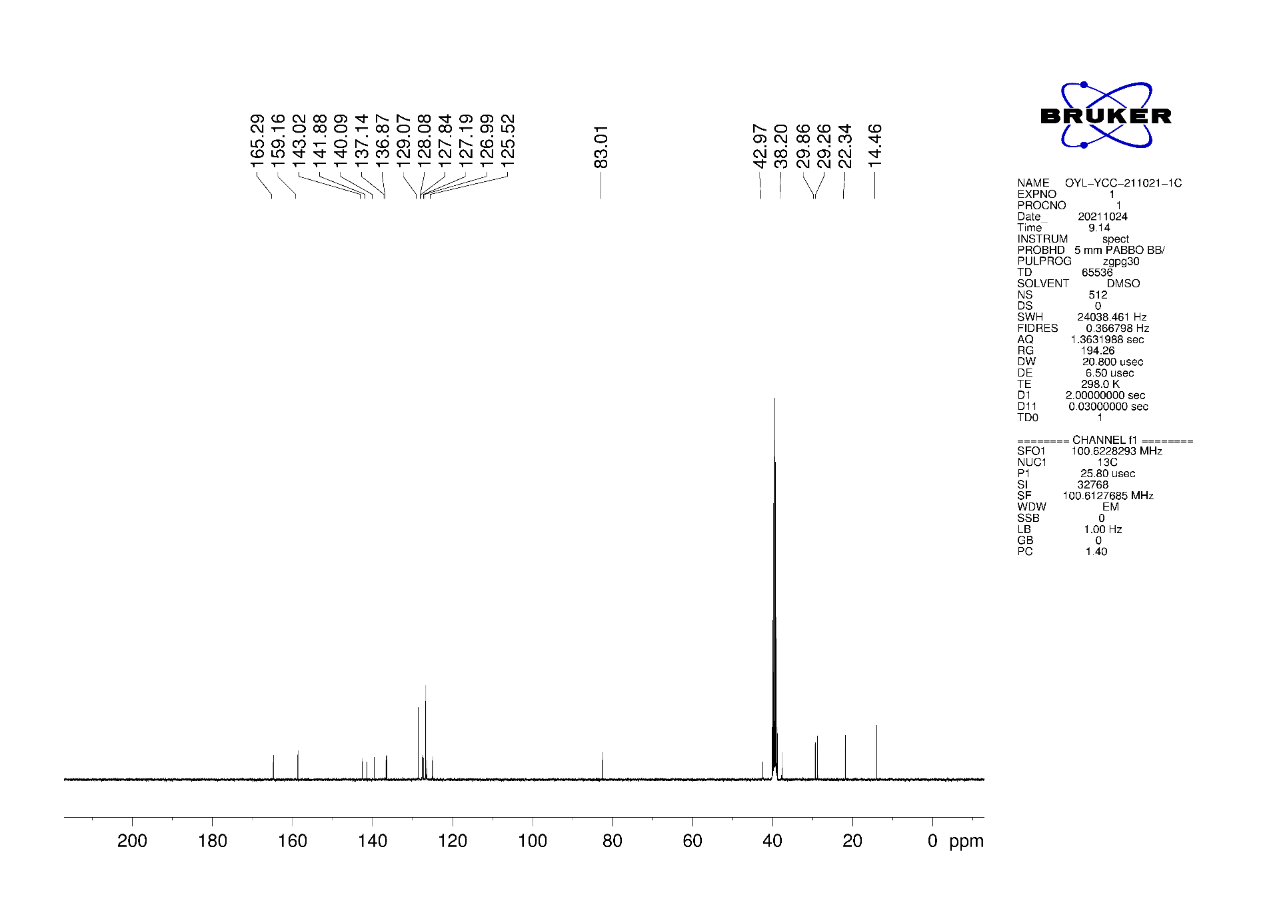

^13^C NMR Spectrum of **SKLB-11L**





^1^H NMR Spectrum of **SKLB-11M**





^13^C NMR Spectrum of **SKLB-11M**




^1^H NMR Spectrum of **SKLB-11N**




^13^C NMR Spectrum of **SKLB-11N**


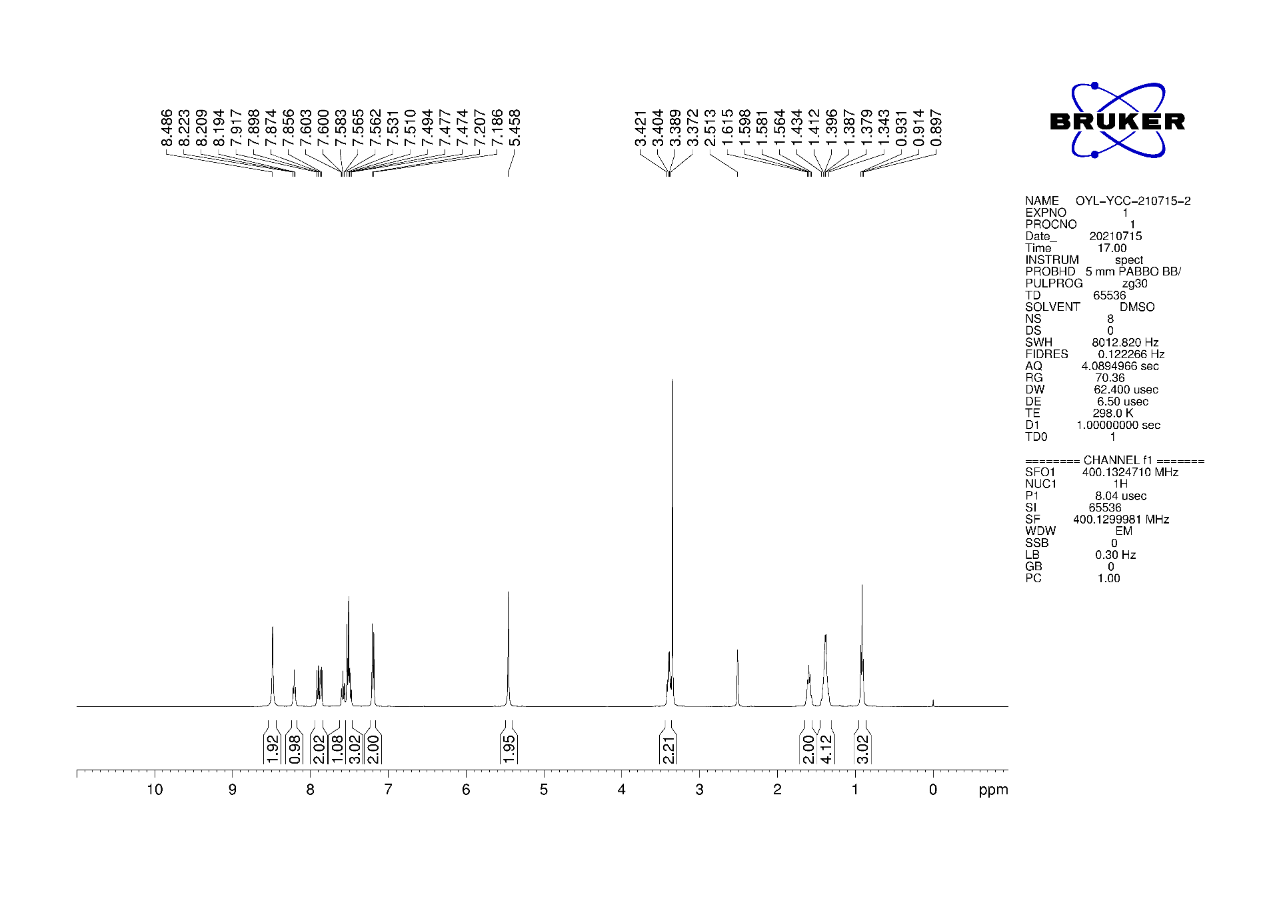

^1^H NMR Spectrum of **SKLB-11O**


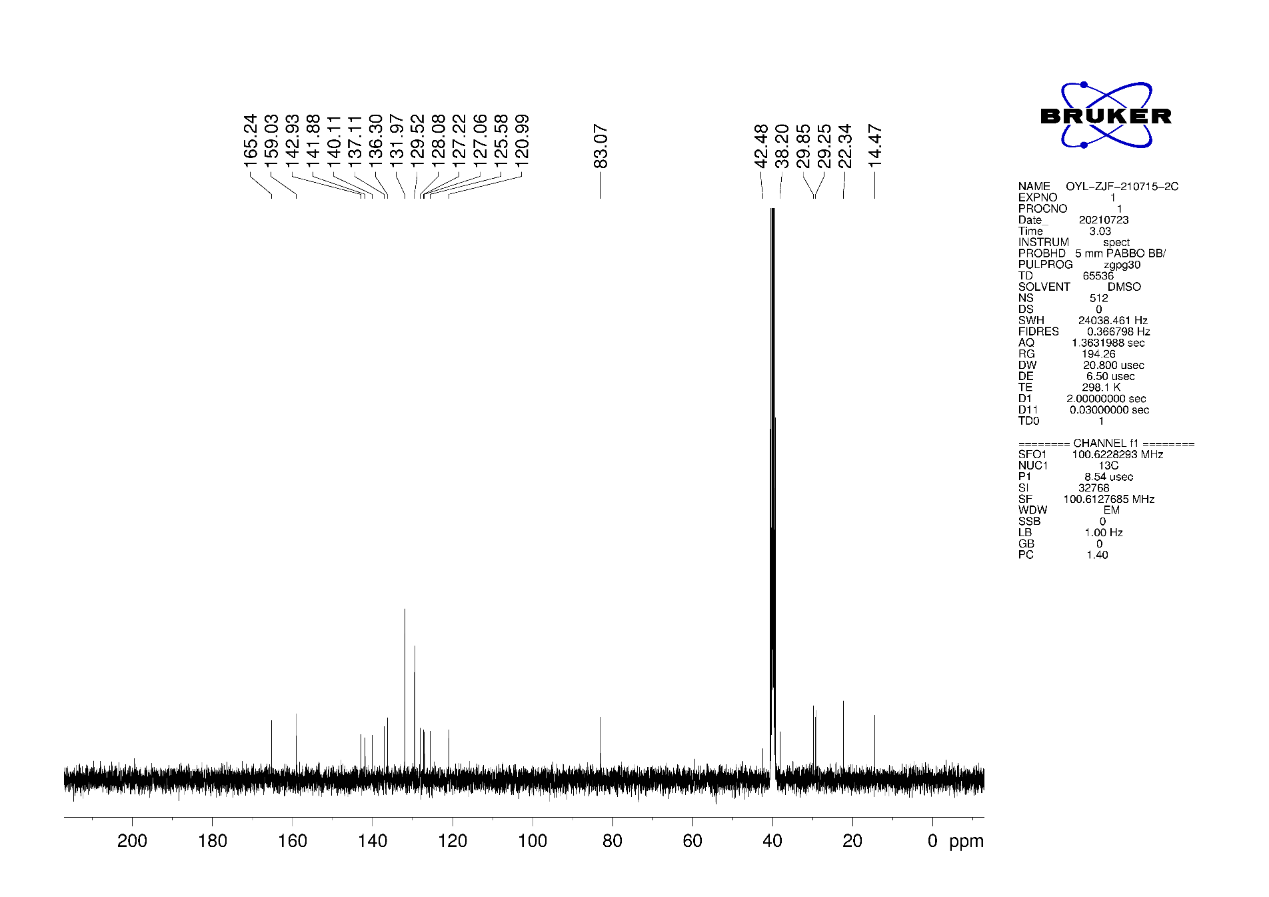

^13^C NMR Spectrum of **SKLB-11O**


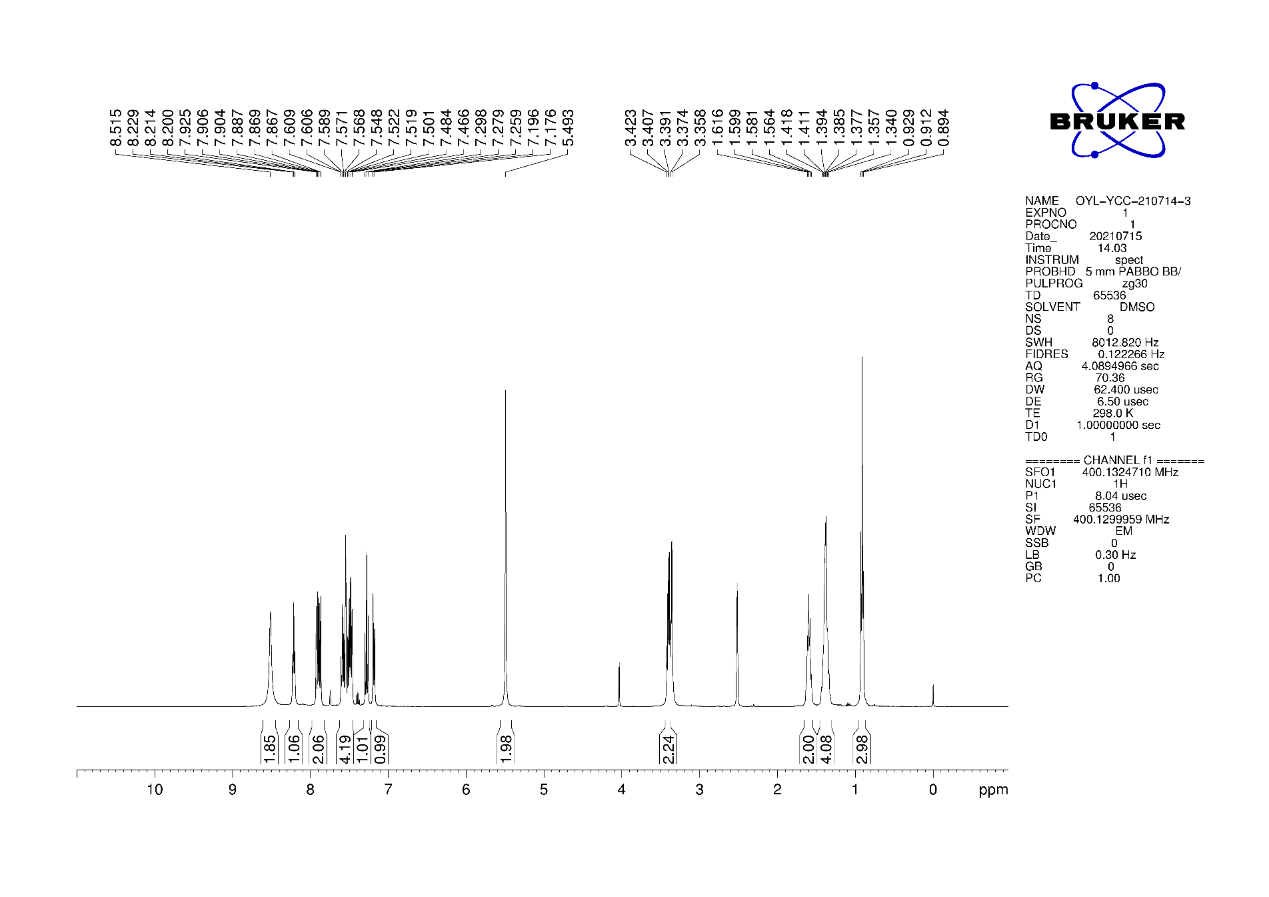

^1^H NMR Spectrum of **SKLB-11P**


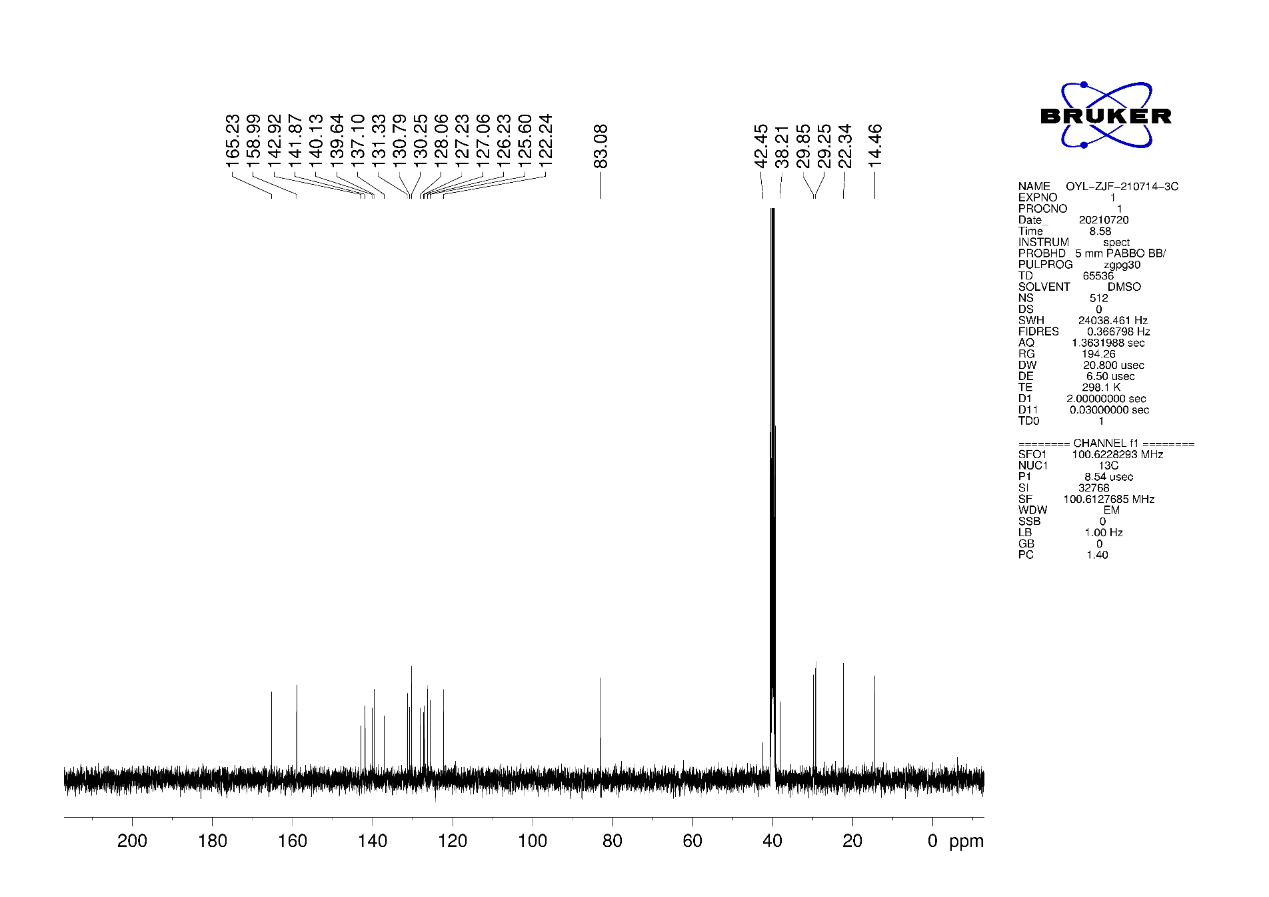

^13^C NMR Spectrum of **SKLB-11P**




^1^H NMR Spectrum of **SKLB-11Q**




^13^C NMR Spectrum of **SKLB-11Q**

**HR-MS(ESI) Spectrum**

HR-MS(ESI) Spectrum of **SKLB-4A**

HR-MS(ESI) Spectrum of **SKLB-4B**

HR-MS(ESI) Spectrum of **SKLB-4C**


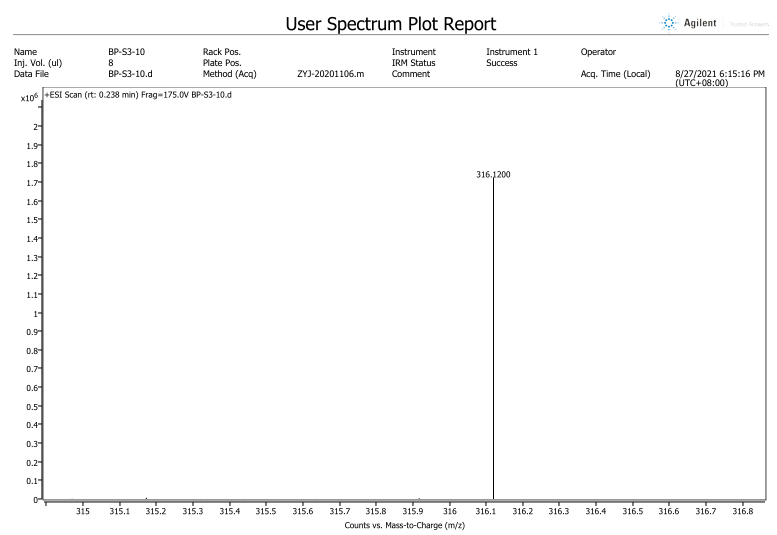


HR-MS(ESI) Spectrum of **SKLB-4D**


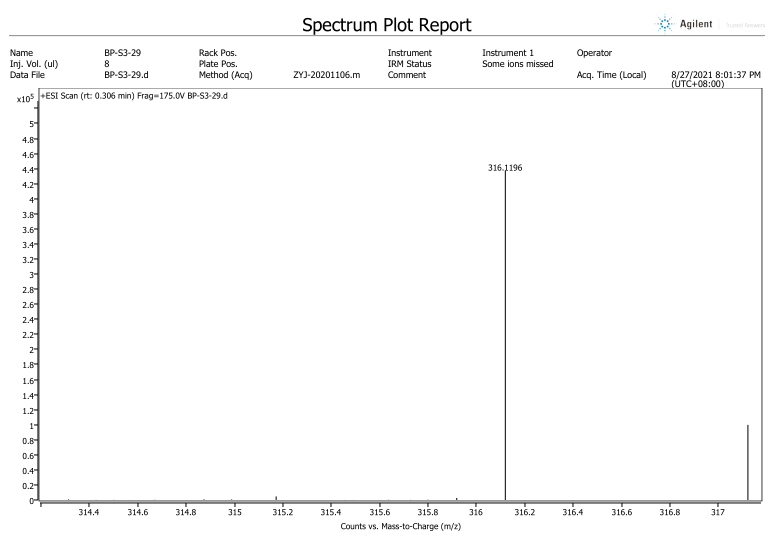


HR-MS(ESI) Spectrum of **SKLB-4E**

HR-MS(ESI) Spectrum of **SKLB-4F**


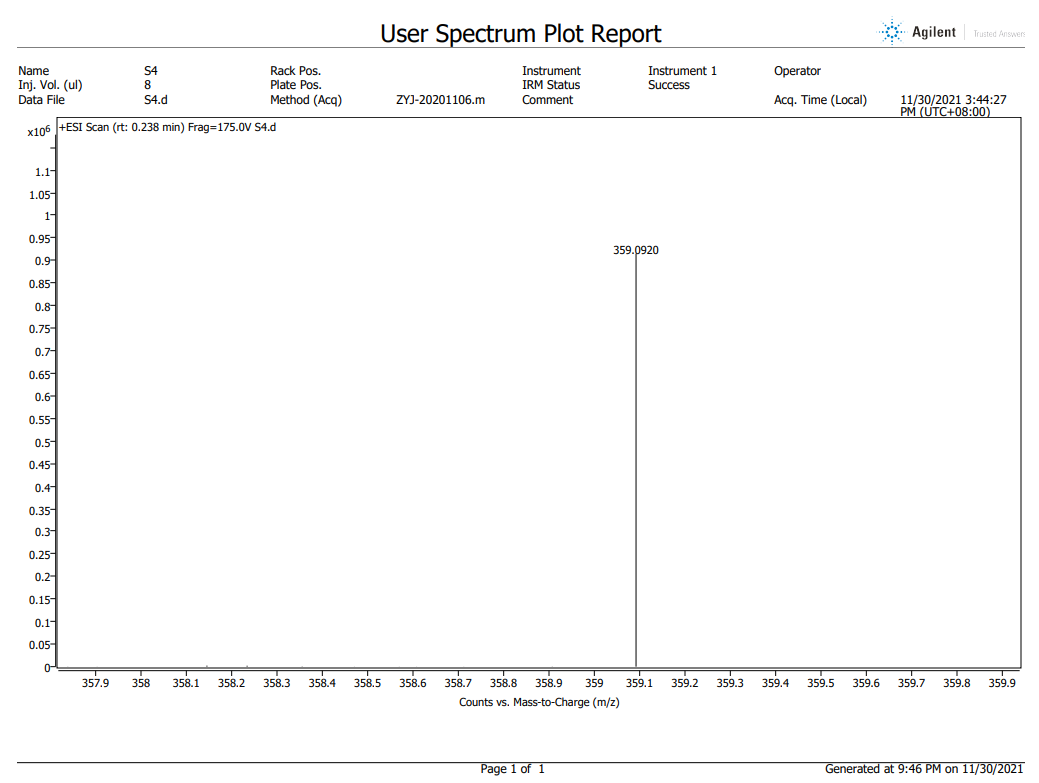


HR-MS(ESI) Spectrum of **SKLB-5A**


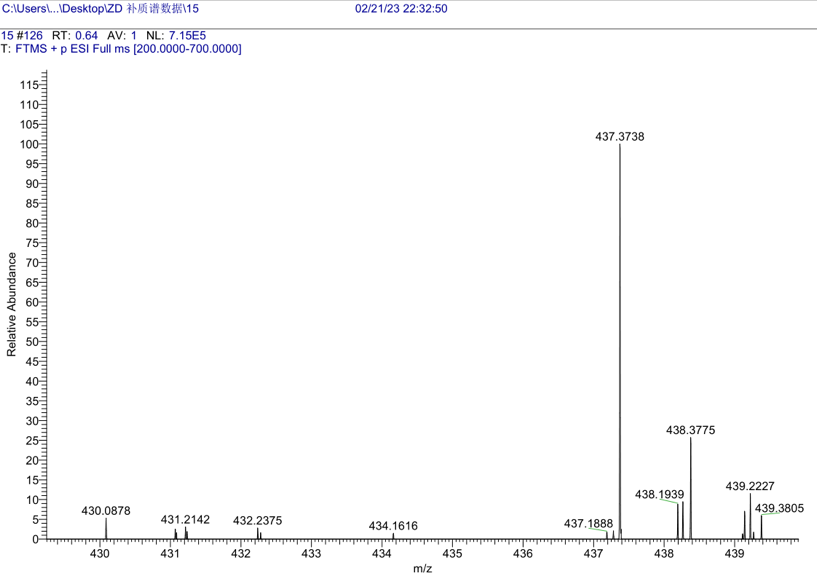


HR-MS(ESI) Spectrum of **SKLB-7A**

HR-MS(ESI) Spectrum of **SKLB-11A**

HR-MS(ESI) Spectrum of **SKLB-11B**

HR-MS(ESI) Spectrum of **SKLB-11C**

HR-MS(ESI) Spectrum of **SKLB-11D**

HR-MS(ESI) Spectrum of **SKLB-11E**

HR-MS(ESI) Spectrum of **SKLB-11F**


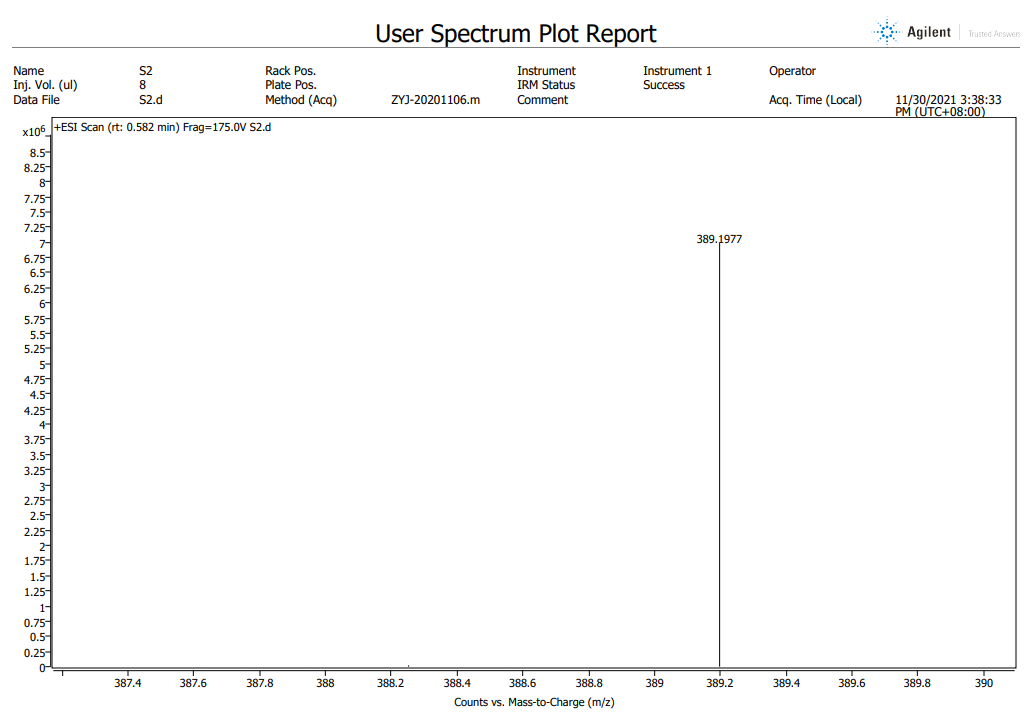


HR-MS(ESI) Spectrum of **SKLB-11G**

HR-MS(ESI) Spectrum of **SKLB-11H**


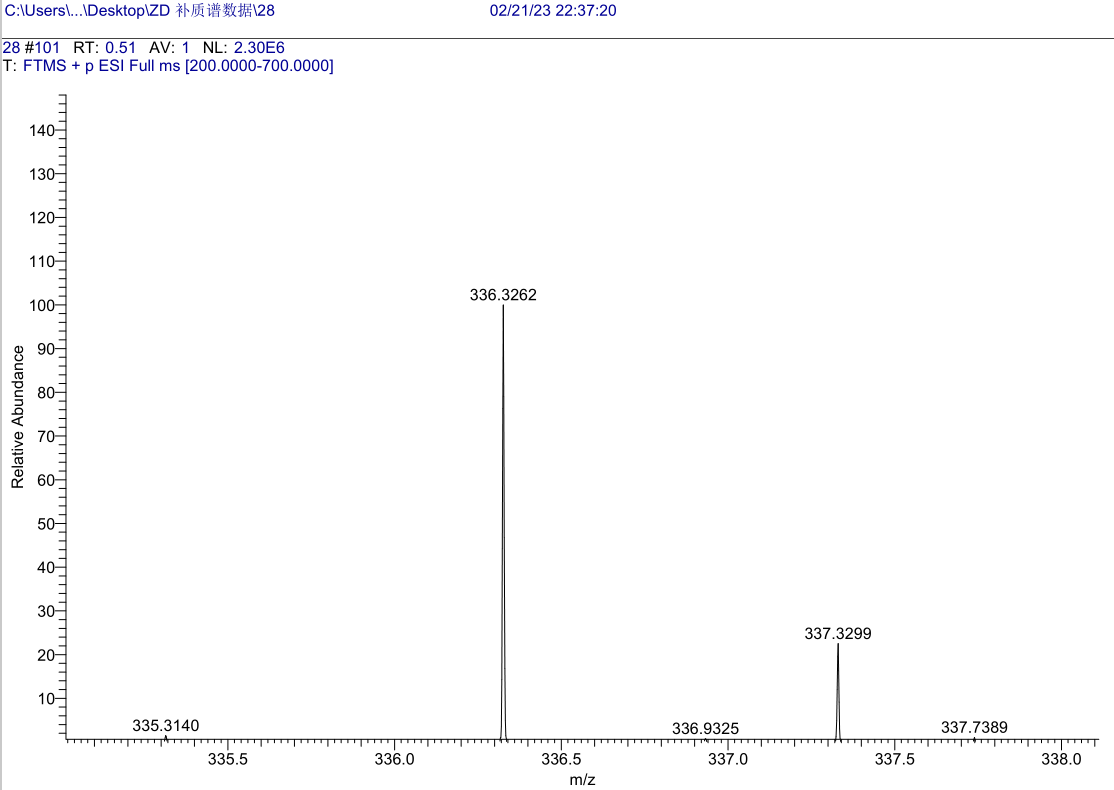


HR-MS(ESI) Spectrum of **SKLB-11I**

HR-MS(ESI) Spectrum of **SKLB-11J**

HR-MS(ESI) Spectrum of **SKLB-11K**


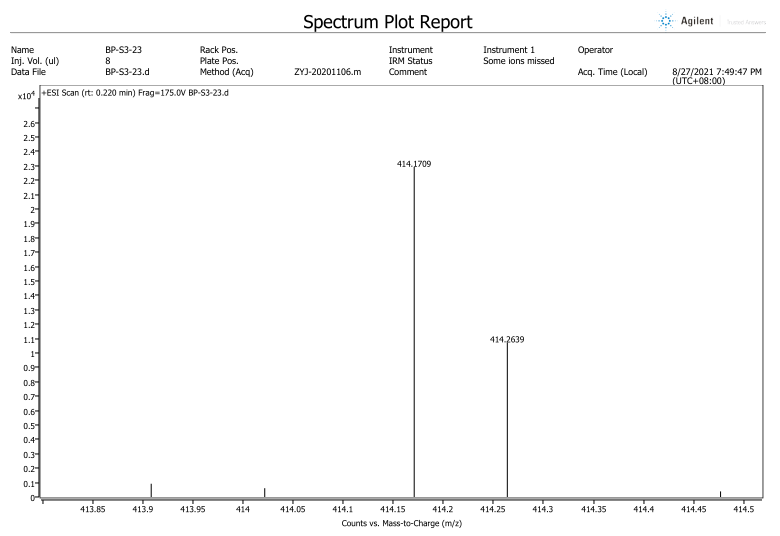


HR-MS(ESI) Spectrum of **SKLB-11L**

HR-MS(ESI) Spectrum of **SKLB-11M**


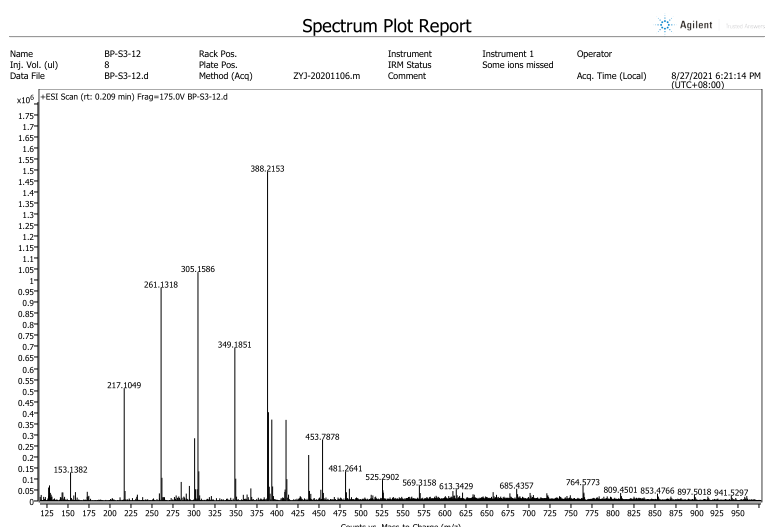


HR-MS(ESI) Spectrum of **SKLB-11N**


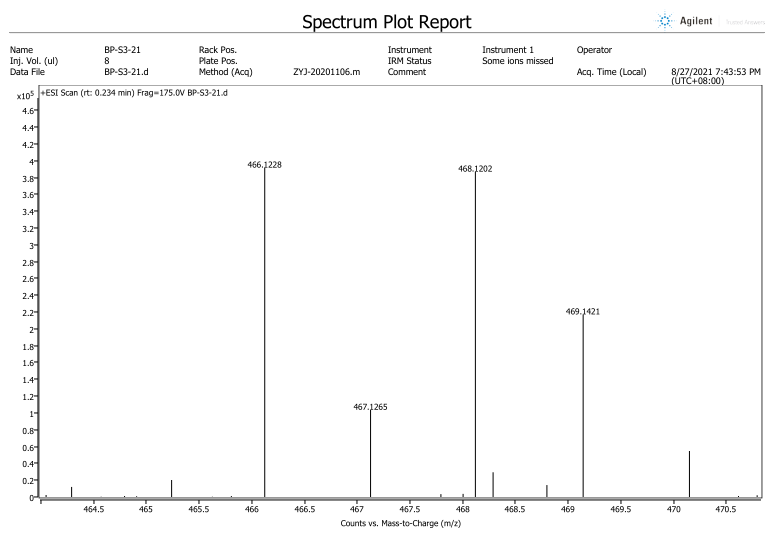


HR-MS(ESI) Spectrum of **SKLB-11O**


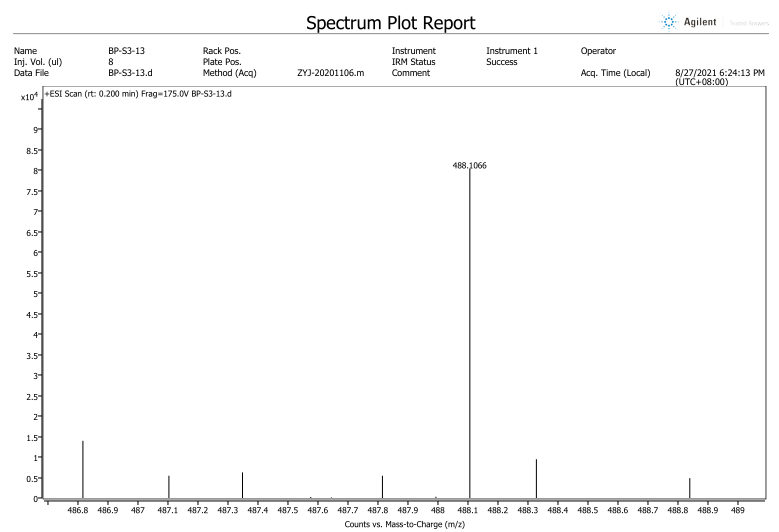


HR-MS(ESI) Spectrum of **SKLB-11P**


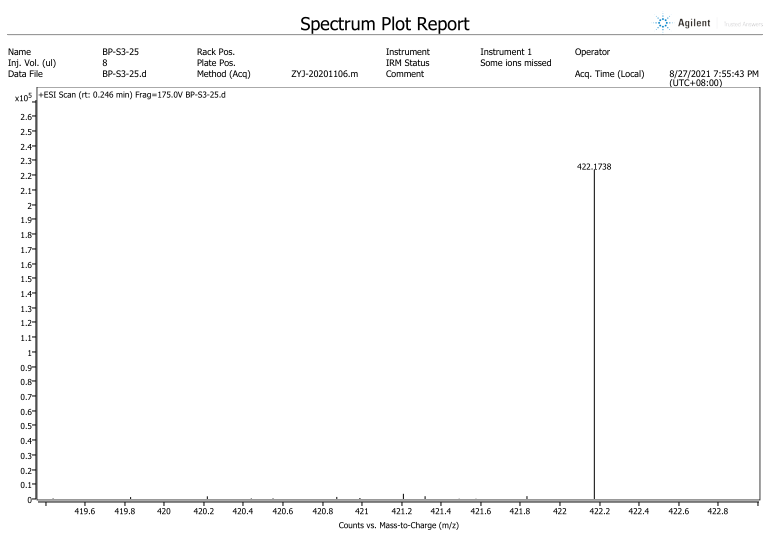


HR-MS(ESI) Spectrum of **SKLB-11Q**

**HPLC chromatogram**


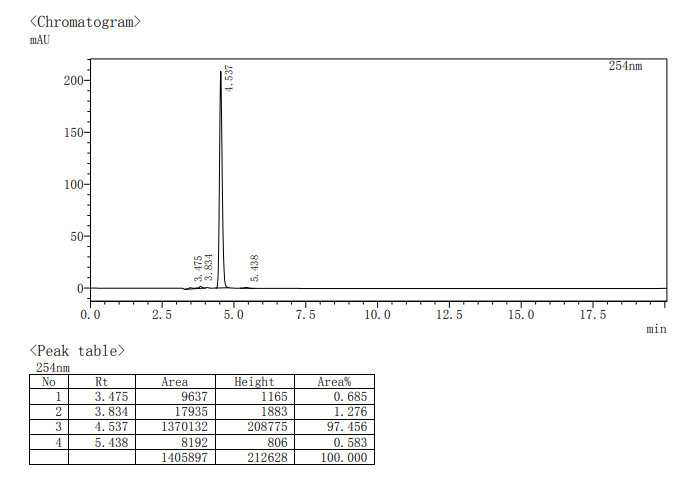


HPLC chromatogram of **SKLB-4A**


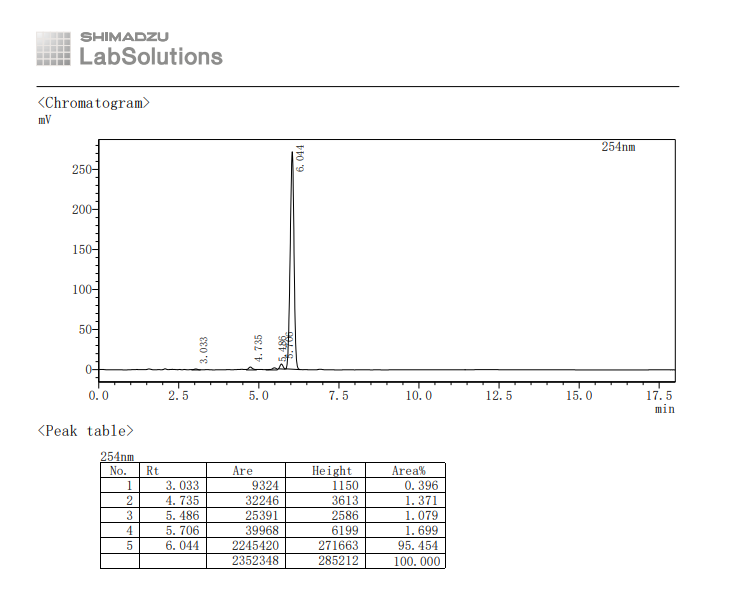


HPLC chromatogram of **SKLB-4B**


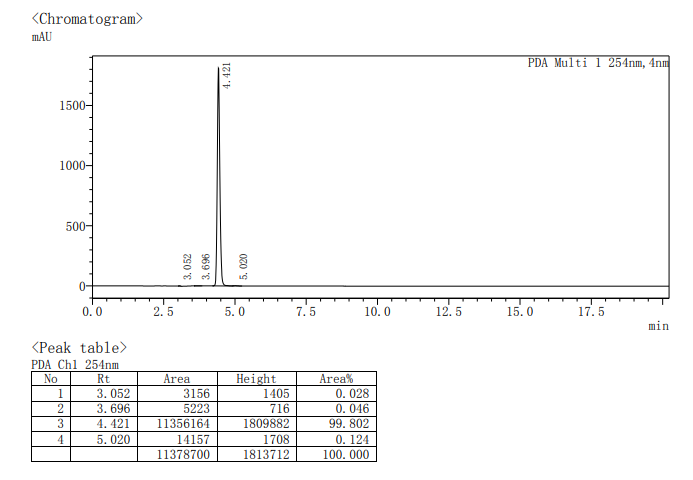


HPLC chromatogram of **SKLB-4C**


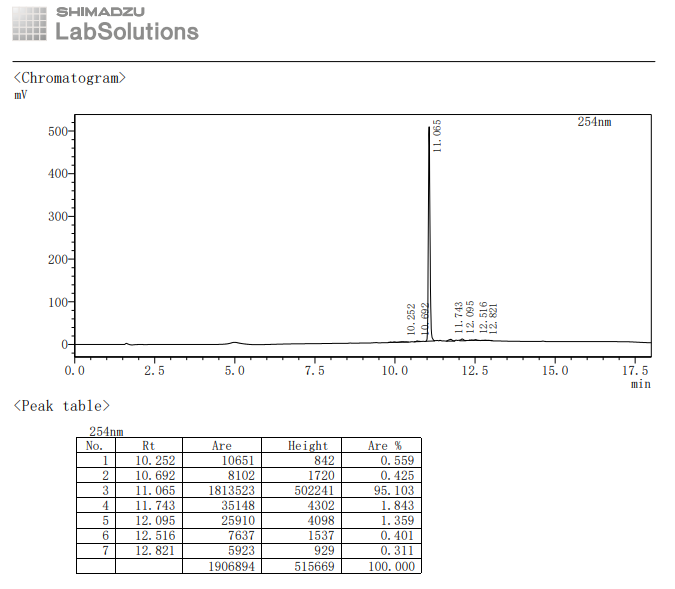


HPLC chromatogram of **SKLB-4D**


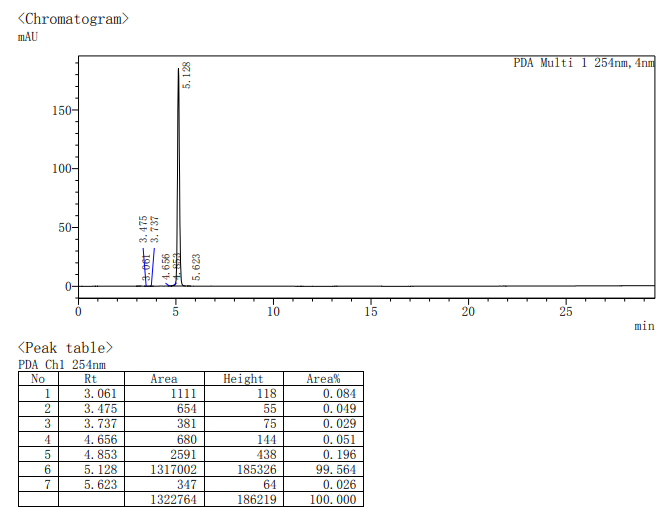


HPLC chromatogram of **SKLB-4E**


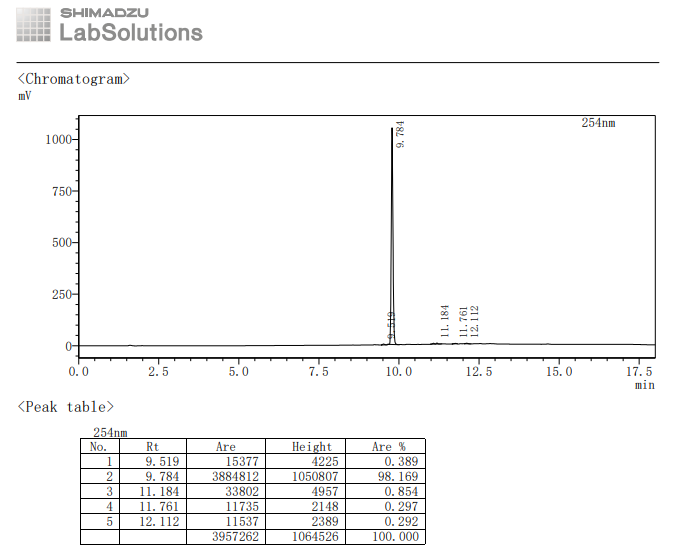


HPLC chromatogram of **SKLB-4F**


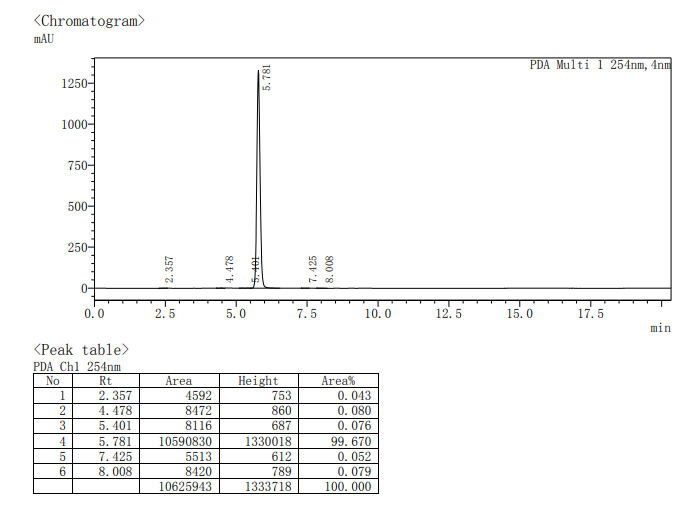


HPLC chromatogram of **SKLB-5A**


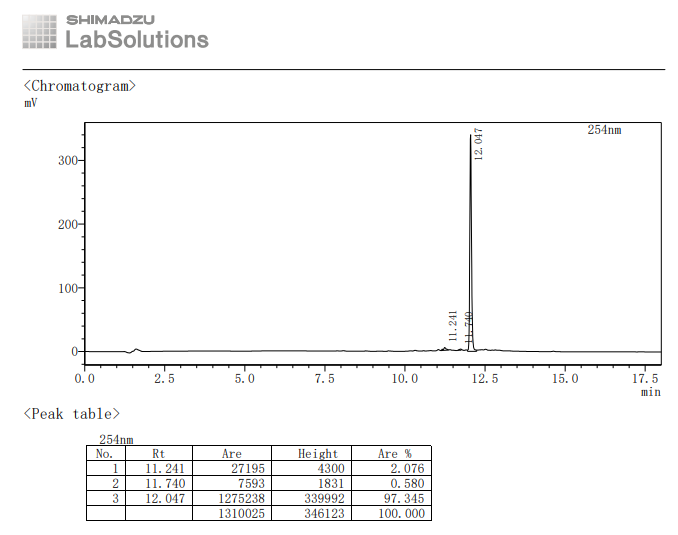


HPLC chromatogram of **SKLB-7A**


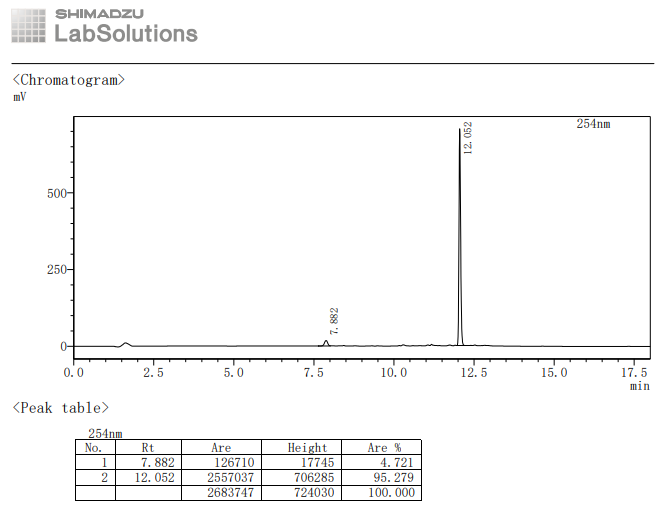


HPLC chromatogram of **SKLB-11A**


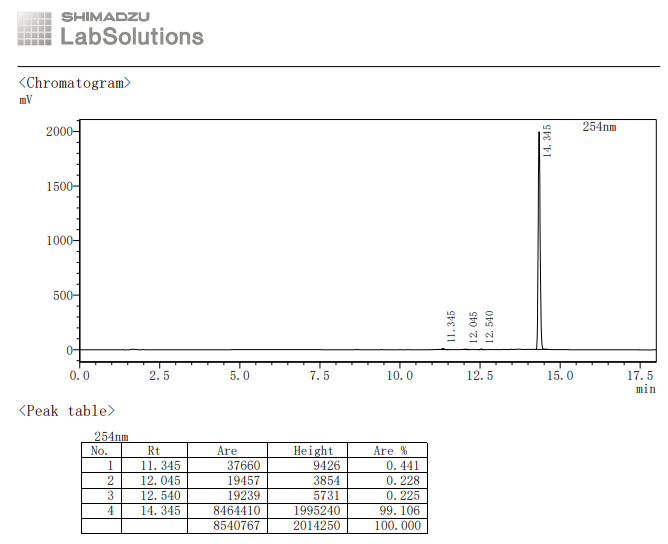


HPLC chromatogram of **SKLB-11B**


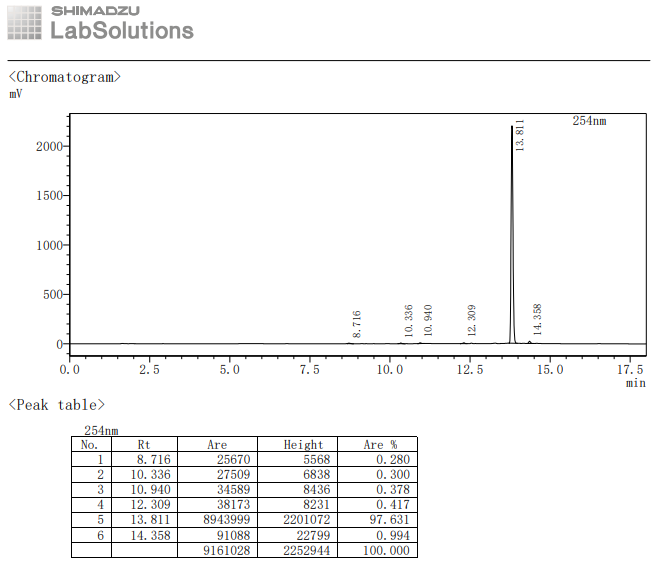


HPLC chromatogram of **SKLB-11C**


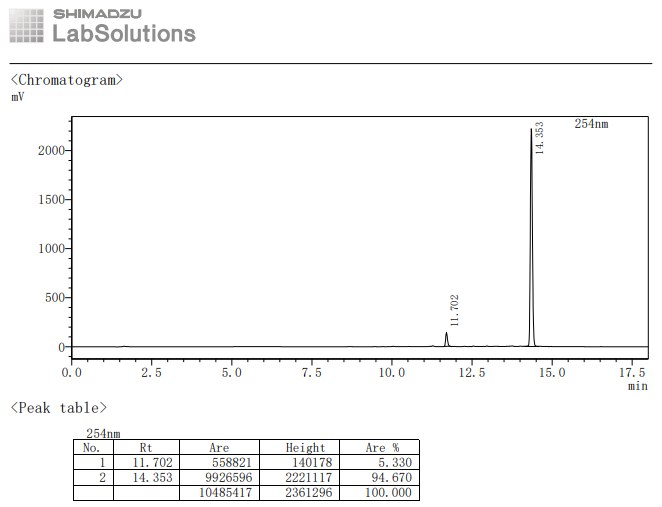


HPLC chromatogram of **SKLB-11D**


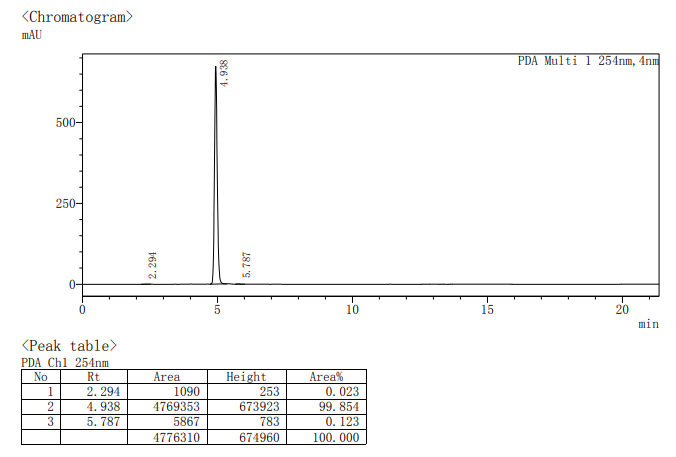


HPLC chromatogram of **SKLB-11E**


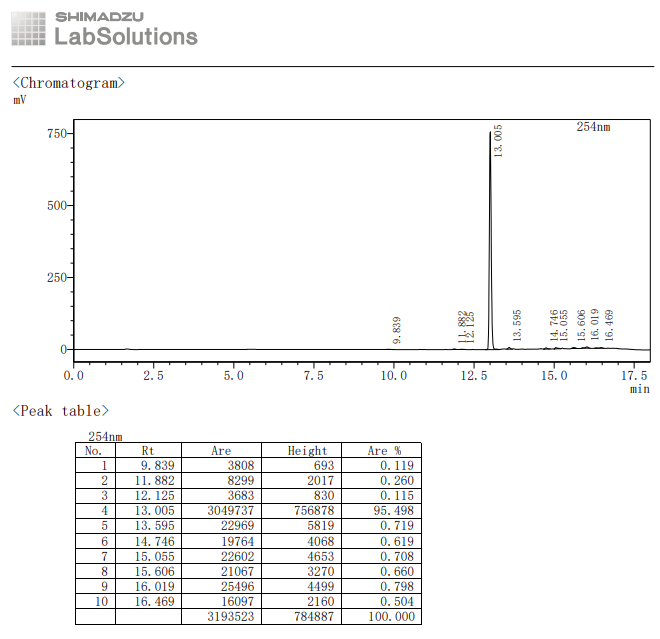


HPLC chromatogram of **SKLB-11F**


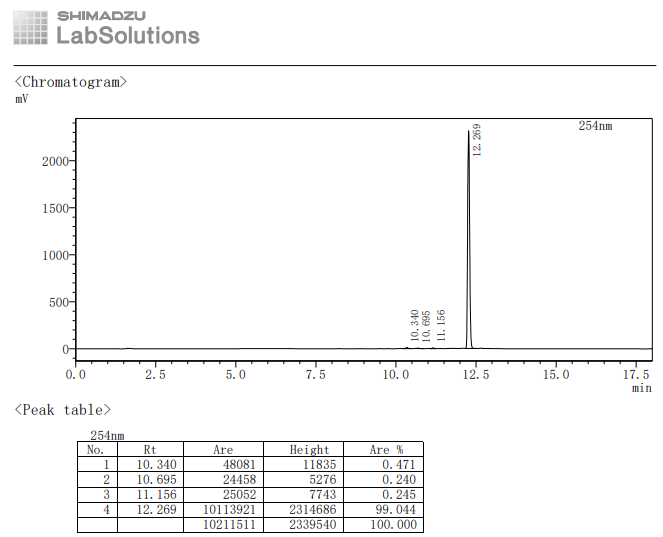


HPLC chromatogram of **SKLB-11G**


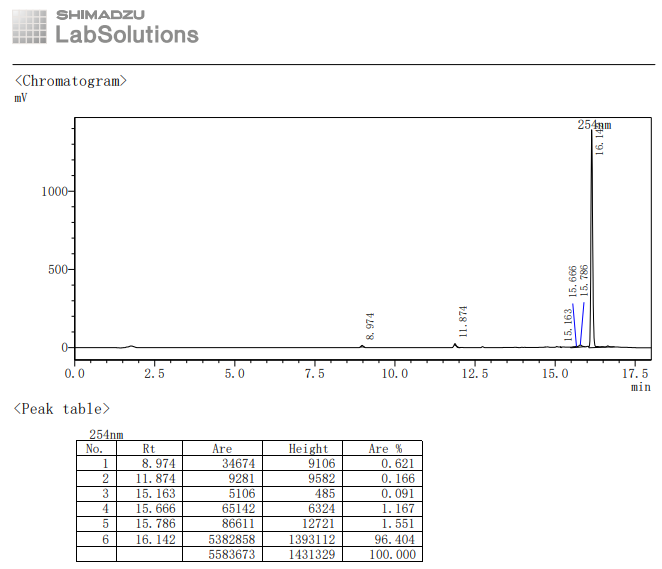


HPLC chromatogram of **SKLB-11H**


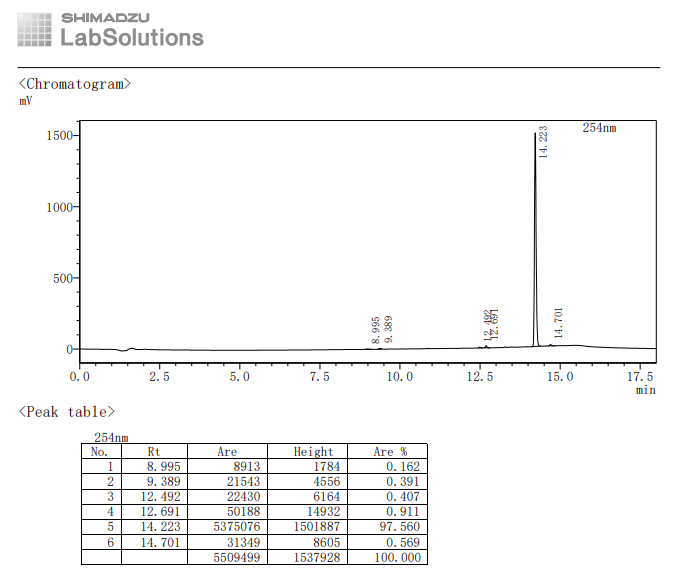


HPLC chromatogram of **SKLB-11I**


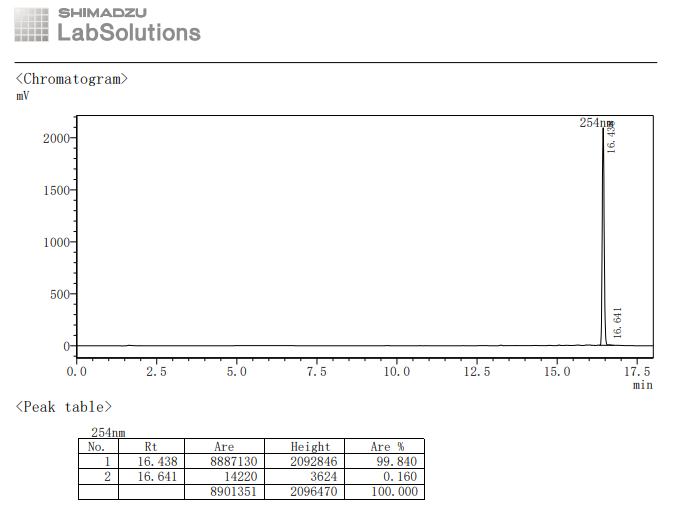


HPLC chromatogram of **SKLB-11J**


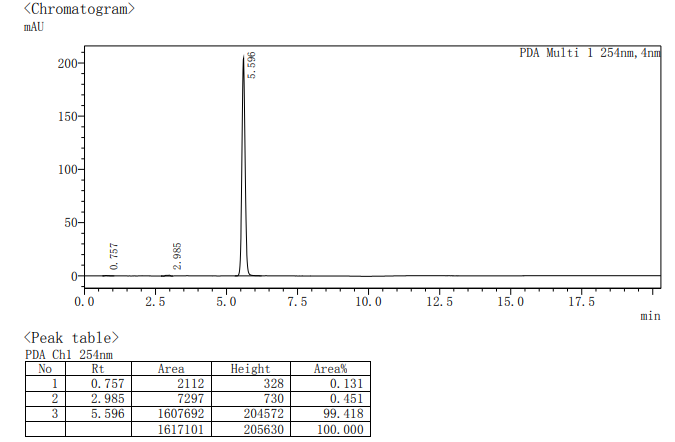


HPLC chromatogram of **SKLB-11K**


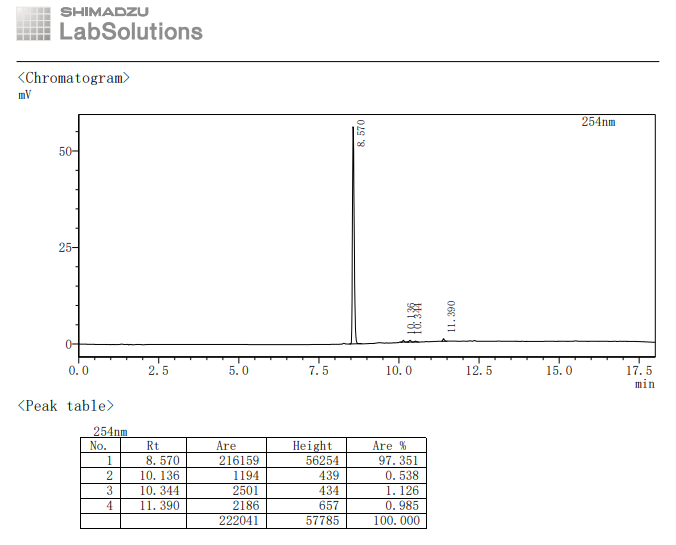


HPLC chromatogram of **SKLB-11L**

HPLC chromatogram of **SKLB-11M**

HPLC chromatogram of **SKLB-11N**

HPLC chromatogram of **SKLB-11O**

HPLC chromatogram of **SKLB-11P**

HPLC chromatogram of **SKLB-11Q**

1. **Uncropped blots**

**6.1 Figure 2D**

**6.2 Figure 2F**

**6.3 Figure 4A**

**6.4 Figure 4G**

**6.5 Figure 4H**

**6.6 Supplementary Figure 4A**

**6.7 Supplementary Figure 6A**

**6.8 Supplementary Figure 16A**
